# Genetic Engineering of the Rock Inhabitant *Knufia petricola* Provides Insight Into the Biology of Extremotolerant Black Fungi

**DOI:** 10.3389/ffunb.2022.862429

**Published:** 2022-04-08

**Authors:** Eileen A. Erdmann, Sarah Nitsche, Anna A. Gorbushina, Julia Schumacher

**Affiliations:** ^1^Department of Materials and the Environment, Bundesanstalt für Materialforschung und -prüfung (BAM), Berlin, Germany; ^2^Department of Biology Chemistry Pharmacy, Freie Universität Berlin, Berlin, Germany; ^3^Department of Earth Sciences, Freie Universität Berlin, Berlin, Germany

**Keywords:** Chaetothyriales, microcolonial fungi, DHN melanin, carotenoids, resistance cassettes, cloning vectors, bimolecular fluorescence complementation, White Collar Complex

## Abstract

Black microcolonial fungi (Ascomycetes from Arthonio-, Dothideo-, and Eurotiomycetes) are stress-tolerant and persistent dwellers of natural and anthropogenic extreme habitats. They exhibit slow yeast-like or meristematic growth, do not form specialized reproduction structures and accumulate the black pigment 1,8-dihydroxynaphthalene (DHN) melanin in the multilayered cell walls. To understand how black fungi live, survive, colonize mineral substrates, and interact with phototrophs genetic methods are needed to test these functions and interactions. We chose the rock inhabitant *Knufia petricola* of the Chaetothyriales as a model for developing methods for genetic manipulation. Here, we report on the expansion of the genetic toolkit by more efficient multiplex CRISPR/Cas9 using a plasmid-based system for expression of Cas9 and multiple sgRNAs and the implementation of the three resistance selection markers genR (geneticin/*nptII*), baR (glufosinate/*bar*), and suR (chlorimuron ethyl/*sur*). The targeted integration of expression constructs by replacement of essential genes for pigment synthesis allows for an additional color screening of the transformants. The black-pink screening due to the elimination of *pks1* (melanin) was applied for promoter studies using GFP fluorescence as reporter. The black-white screening due to the concurrent elimination of *pks1* and *phs1* (carotenoids) allows to identify transformants that contain the two expression constructs for co-localization or bimolecular fluorescence complementation (BiFC) studies. The co-localization and interaction of the two *K. petricola* White Collar orthologs were demonstrated. Two intergenic regions (*igr1, igr2*) were identified in which expression constructs can be inserted without causing obvious phenotypes. Plasmids of the pNXR-XXX series and new compatible entry plasmids were used for fast and easy generation of expression constructs and are suitable for a broad implementation in other fungi. This variety of genetic tools is opening a completely new perspective for mechanistic and very detailed study of expression, functioning and regulation of the genes/proteins encoded by the genomes of black fungi.

## Introduction

The melanized fungus *Knufia petricola* (syn. *Sarcinomyces petricola*) (Eurotiomycetes, Chaetothyriales) is known for colonizing and decomposing ancient marble in the Mediterranean (Wollenzien et al., 1997; Sert et al., 2007; Marvasi et al., 2012; Isola et al., 2015). Strain A95 was isolated from a marble surface in Greece and exhibits the typical characteristics of extremotolerant black fungi, such as slow yeast-like growth, absence of specialized reproduction structures, the production of protecting metabolites including black 1,8-dihydroxynaphthalene (DHN) melanin, orange to red carotenoids, mycosporines, and extracellular polysaccharides (EPS) (Volkmann et al., 2003; Gorbushina et al., 2008; Nai et al., 2013; Breitenbach et al., 2018; Flieger et al., 2018; Knabe and Gorbushina, 2018). Microcolonial black fungi—also called meristematic fungi or black yeasts—belong to the classes of Eurotiomycetes, Dothideomycetes and Arthoniomycetes, are ubiquitous and colonize unusual niches such as salterns, rock surfaces in Antarctic dry valleys, hot deserts, as well as monuments, buildings and solar panels (Ruibal et al., 2009; Cantrell et al., 2017; Martin-Sanchez et al., 2018; Selbmann et al., 2020; Prenafeta-Boldú et al., 2021). Their extreme tolerance, particularly their ability to survive in habitats with high temperatures and UV radiation, water and nutrient scarcity, is intriguing and makes their study interesting in the context of climate change and its consequences for biodiversity. In contrast to fast-growing molds, little is known about microcolonial black fungi as genetic studies are hampered by the lack of sexual cycles and considerable difficulties to transform these fungi.

**Figure 1:**
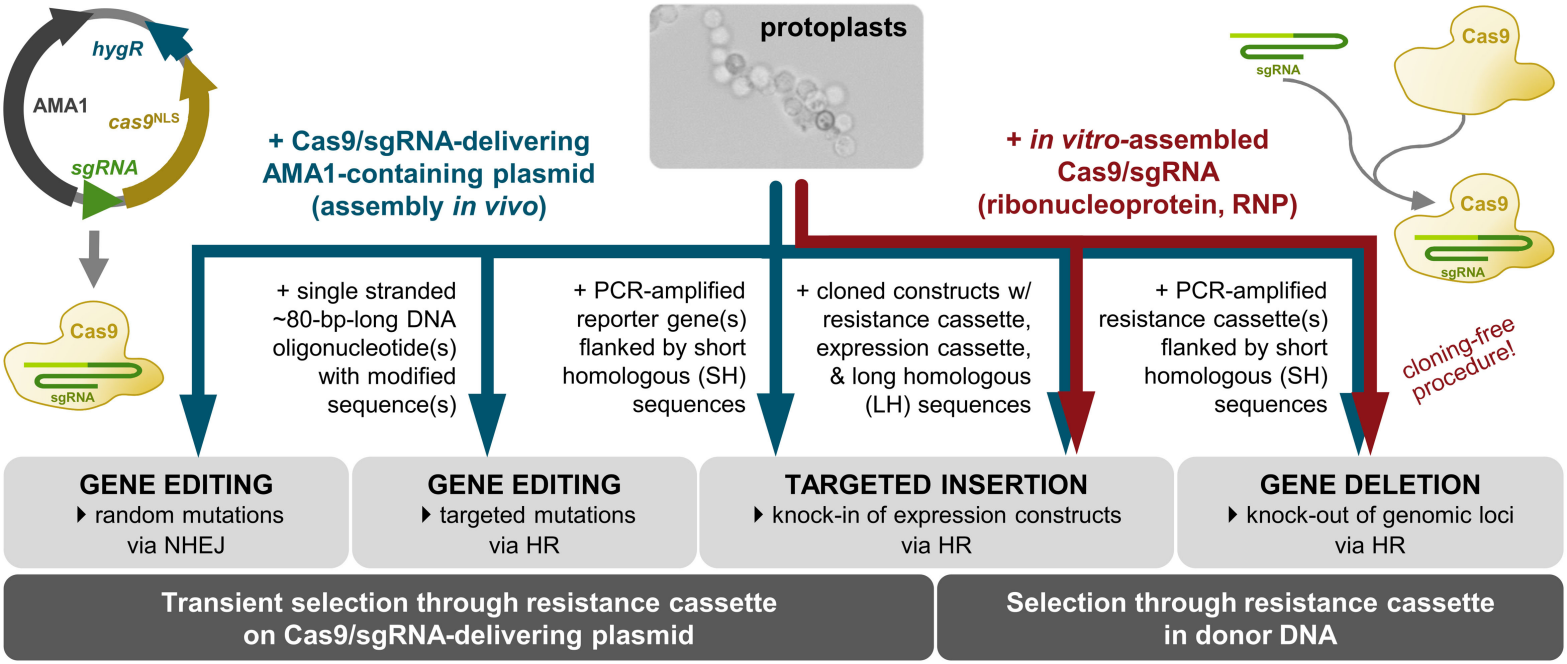
CRISPR/Cas9 applications for targeted modification of the *K. petricola* genome. The use of AMA1-containing plasmids for expression of Cas9 and target-specific sgRNA (Nødvig et al., 2015, 2018) enables the generation of marker-free mutants when transient selection is applied on the plasmid-encoded hygromycin (HYG) resistance. The insertion of double strand breaks (DSBs) in specific DNA sequences results in random mutations *via* non-homologous end-joining (NHEJ) or in targeted mutations when modified single stranded oligonucleotide(s) are provided as repair templates for homologous recombination (HR). By co-transformation of Cas9/sgRNA-delivering plasmids with PCR-generated or cloned constructs (donor DNA) consisting of resistance (and expression) cassettes flanked by homologous sequences, sequences in the genome can be replaced (knock-out) or sequences can be integrated at defined genomic loci (knock-in). The co-transformation of protoplasts with pre-assembled ribonucleoproteins (RNPs) of *in vitro*-synthesized target-specific sgRNA and purified Cas9 functions together with resistance cassette-containing donor DNA. Combinations of RNPs with PCR-amplified donor DNA (resistance cassette with primer-generated 75-bp-long homologous sequences) enable the cloning-free generation of gene replacement (KO) mutants (Voigt et al., 2020) (this study).

The development of protocols for generation, transformation and regeneration of protoplasts (Noack-Schönmann et al., 2014a) paved the way for genetic engineering of the *K. petricola* genome for studying cell biology and gene functions. The expression of ectopically integrated genes for fluorescent proteins demonstrated that fluorescence microscopy applications in black fungi are feasible though the highly melanized cell walls (Voigt et al., 2020). Like in other fungi the rates of homologous recombination (HR) in conventional gene replacement approaches are rather low. To overcome this difficulty, the CRISPR (clustered regularly interspaced short palindromic repeats)/Cas9 system (Doudna and Charpentier, 2014) was adopted. The targeted insertion of a double strand break (DSB) in the DNA sequence of interest by the RNA-guided nuclease Cas9 increases rates of HR when a repair template (stretch of homologous sequence to the targeted DNA) is provided and generates random mutations at the inserted DSB due to non-homologous end-joining (NHEJ) repair in the absence of repair templates. Two strategies for delivering Cas9 and target-specific single guide RNA (sgRNA) to the nuclei of *K. petricola* are available and shown to be equally efficient for generating gene replacement mutants using long homologous (LH) sequences (cloned constructs in which a resistance cassette is flanked by ~1-kb-long noncoding regions of the gene of interest) and short homologous (SH) sequences (PCR-generated constructs in which a resistance cassette is flanked by ~75-bp-long noncoding regions) (Voigt et al., 2020). For ribonucleoprotein (RNP)-based CRISPR/Cas9, the target-specific sgRNA is synthesized *in vitro*, assembled with purified Cas9 and the RNPs are added together with the donor DNA to the protoplasts. For a plasmid-based approach, i.e., the transient expression of both components from a plasmid for *in-vivo* assembly of the RNP, the protocols and plasmids developed by Mortensen et al. have been adopted. These plasmids (pAMA/ribozyme) contain a hygR cassette for selection in fungi, a cassette for expression of *cas9*, a cassette for expression of a sgRNA flanked by two ribozymes as mRNA, and the self-replicating sequence AMA1 from *Aspergillus nidulans* (Nødvig et al., 2015) (Supplementary Figure 1). The AMA1-bearing plasmids have a moderate half-life in *K. petricola* enabling different applications. When they are combined with donor DNA and selection is applied for the stable integration of the contained resistance cassettes, the CRISPR plasmids are usually gone when transformants are transferred for the first time to non-selective medium. Besides, the contained hygR cassette allows for transient selection on the CRISPR plasmid during transformation without selectable donor DNA, enabling gene editing approaches (Figure 1).

To evaluate the different strategies for their suitability in *K. petricola*, essential genes for pigment synthesis were targeted. The mutation of *pks1* encoding the key enzyme of DHN melanin synthesis (polyketide synthase 1) results in pink mutants. The effect of the mutation of *phs1* encoding the key enzyme of carotenoid synthesis (phytoene synthase 1) is not apparent in the wild-type background but the mutation of *phs1* results in pigment-deficient (white) mutants when combined with the *pks1* mutation (Voigt et al., 2020). Therefore, the study by Voigt, Knabe et al. provided first *K. petricola* mutants that have been used in other studies for evaluating the role of DHN melanin and carotenoids in dissolution of olivine, penetration of marble, and the composition of the EPS (Gerrits et al., 2020, 2021; Tonon et al., 2020; Breitenbach et al., submitted).

To open new perspectives on the challenging functional analysis of black fungi, we aimed at widening, modernizing and at the same time streamlining the available genetic tools. This study describes the expansion of the toolkit for the modification of the *K. petricola* genome by more efficient multiplexed CRISPR/Cas9, three additional selection marker systems and color-based approaches for identifying transformants with successful integration of one or two expression constructs. Inventing the new black-pink screening method was combined with the objectives to quantify the activities of different promoters commonly used in other fungi and to identify an inducible one in *K. petricola*. The black-white screening was used for co-localization and bimolecular fluorescence complementation (BiFC) studies, revealing the formation of the White Collar Complex in *K. petricola*. Furthermore, neutral genomic regions for the targeted insertion of expression constructs were identified. These neutral insertion sites enabled the study of integrated *gfp* constructs—leaving all genes intact—by fluorescence microscopy in a melanin-containing wild-type-like background and can be used for the complementation of deletion mutants.

## Materials and Methods

### Cultivation of *K. petricola*

*Knufia petricola* A95 (CBS 123872) was used as recipient (WT) for genetic manipulation. *K. petricola* genes analyzed in this study have been described previously (Voigt et al., 2020) or were identified in the genome sequence of A95 (~28 Mb, ~340×, 12 contigs) (Heeger et al., unpublished) using the tblastN tool of Geneious Prime 2021 (Biomatters Ltd., Auckland, NZL). For the sequences see GenBank accessions OM802156-OM802160 or Supplementary Sequences 1–5. A95 and derivatives (Supplementary Table 1) were cultivated in malt extract broth/agar (MEB/MEA) at 25 °C in darkness (Nai et al., 2013). Basal synthetic medium was SDNG (synthetic-defined–nitrate–glucose) [0.17% Difco™ Yeast Nitrogen Base without Amino Acids and Ammonium Sulfate (BD Biosciences, Franklin Lakes, NJ, USA), 0.3% NaNO_3_, 2% glucose, 2% agar]. SDYG contained 0.1% yeast extract instead of NaNO_3_. For inoculation of growth assays, cells were taken from surface-grown colonies, resuspended in phosphate-buffered saline (PBS), and dispersed using glass beads (3–5 mm) and a Ribolyser (Hybaid) (20 s at 40 Hz). Cell titers of 1:10 dilutions were determined using a Thoma cell counting chamber and adjusted with PBS to 1 × 10^6^ cells ml^−1^ for droplet assays and 2.5 × 10^6^ cells ml^−1^ for growth assays. Dilution series down to 1 × 10^3^ cells ml^−1^ were prepared and used for inoculation of solidified media with 10-μl droplets. For growth assays, 200 μl of the cell suspensions (2.5 × 10^6^ cells ml^−1^) were evenly distributed with 10 glass beads (3–5 mm) on agar. Stock solutions of selective agents were hygromycin B (HYG; AppliChem, Darmstadt, GER) (41 mg ml^−1^ H_2_O), nourseothricin (NTC; Werner BioAgents GmbH, Jena, GER) (100 mg ml^−1^ H_2_O), geneticin (G418; Sigma-Aldrich, St. Louis, MO, USA) (50 mg ml^−1^ H_2_O), glufosinate ammonium (GFS; ChemPur, Karlsruhe, GER) (100 mg ml^−1^ H_2_O), and chlorimuron ethyl (CME; Alfa Aesar, Heysham, UK) (100 mg ml^−1^ DMF).

### Standard Molecular Methods

Genomic DNA from *K. petricola* was prepared as described previously (Voigt et al., 2020). DNA was mixed with Midori Green Direct (Biozym Scientific GmbH, Oldendorf, GER) and separated in 1% agarose gels using the MassRuler™ DNA Ladder Mix (Thermo Scientific, Waltham, MS, USA) as size standard. Total RNA from *K. petricola* was extracted using the TRI Reagent RNA Isolation Reagent (Sigma-Aldrich, St. Louis, MO, USA) and purified using the Monarch RNA Cleanup Kit (New England Biolabs, NEB, Ipswich, MA, USA). 1 μg of total RNA was submitted to reverse-transcription (RT) using the iScript gDNA Clear cDNA Synthesis Kit (Bio-Rad Laboratories Inc., Hercules, CA, USA). Standard PCR reactions were performed using desalted primers from Eurofins Genomics Germany GmbH (Ebersberg, GER) listed in Supplementary Table 2, the Q5® High-Fidelity DNA Polymerase (NEB) for cloning and sequencing purposes and the Taq DNA Polymerase (NEB) for diagnostic applications. PCR products were purified with the Monarch™ PCR & DNA Cleanup Kit (NEB) and sequenced. Plasmids listed in Supplementary Table 3 were assembled by homologous recombination in *Saccharomyces cerevisiae* FY843 (Oldenburg et al., 1997; Schumacher, 2012) or using the NEBuilder® HiFi DNA Assembly Cloning Kit (NEB). Plasmid DNA from *Escherichia coli* and *S. cerevisiae* was extracted with the Monarch® Plasmid Miniprep Kit (NEB). For extraction of larger amounts of plasmid DNA from *E. coli* the NucleoBond® Xtra Midi Kit (Macherey-Nagel, Düren, GER) was used. Sequencing of PCR fragments and plasmids was accomplished with the Mix2Seq Kit at Eurofins Genomics. Cloning procedures were supported by using SnapGene® 4.0.8 (GSL Biotech LLC, Chicago, IL, USA).

### Transformation of *K. petricola*

**Figure 2:**
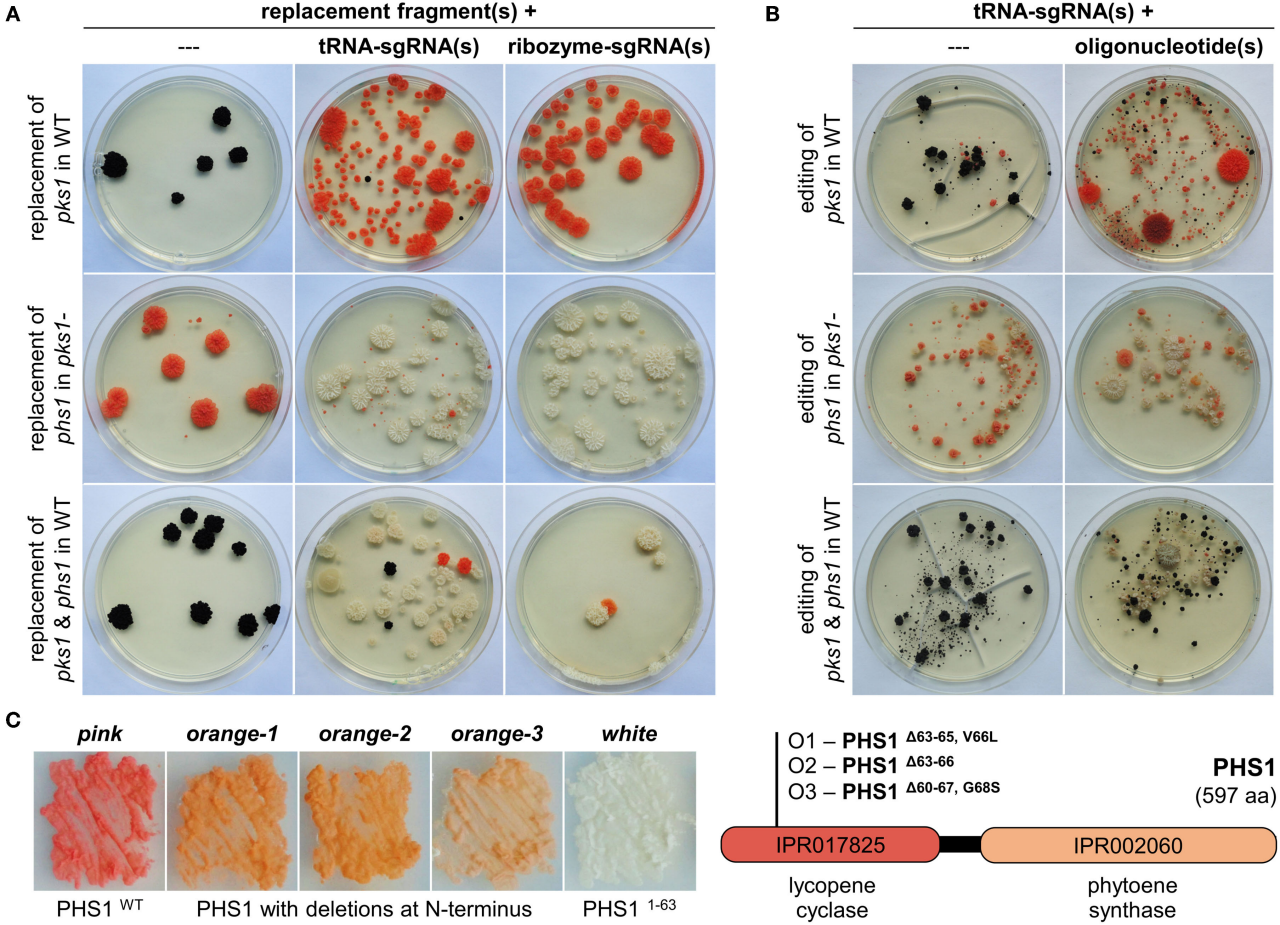
CRISPR/Cas9-assisted mutations using sgRNAs liberated from tRNA splicing cassettes in *K. petricola*. **(A)** The expression of two sgRNAs from a tRNA splicing cassette increases the rate of double replacement mutants. Essential genes for pigment synthesis were targeted to compare efficiencies of sgRNA(s) released from tRNA splicing cassettes (this study, Supplementary Table 3) and ribozyme cassettes (Voigt et al., 2020) of AMA1-bearing CRISPR/Cas9 plasmids: replacement of *pks1* in WT for pink mutants, replacement of *phs1* in melanin-deficient *pks1-* for white mutants, and double replacement of *pks1* and *phs1* in WT for white mutants. Short-homology (SH) replacement fragments for *pks1* with natR cassette and *phs1* with hygR cassette were amplified by PCR. Protoplasts were transformed with donor DNA alone (negative controls; 1D, 2D, 3D) or in combination with AMA1-containing plasmids expressing Cas9 and respective sgRNAs from tRNA splicing cassettes (tRNA-sgRNA; 1C, 2C, 3C) or ribozyme cassettes (ribozyme-sgRNA; RC1, RC2, RC3) as specified in Supplementary Table 4. Regenerated protoplasts were overlaid with agar supplemented with NTC (Δ*pks1*), HYG (Δ*phs1*) or both (Δ*pks1*/Δ*phs1*). Representative transformation plates after 5 weeks of incubation are shown. For summary of the results of three independent experiments see Supplementary Figure 3A. **(B)** sgRNAs from tRNA splicing cassettes mediate efficient double gene editing when combined with oligonucleotides. WT and *pks1*- protoplasts were transformed with pAMA/tRNA-*pks*1^PS2^, pAMA/tRNA-*phs*1^PS1^, or pAMA/tRNA-*pks*1^PS2^-*phs*1^PS1^ (Supplementary Table 3) without donor DNA (random editing *via* NHEJ; 1A, 2A, 3A) or with the 80-bp-long single-stranded oligonucleotides *pks1*-oligo2.7 and *phs1*-oligo1 containing specific mutations (Voigt et al., 2020) (targeted editing *via* HR; 1B, 2B, 3B) as summarized in Supplementary Table 4. Regenerated protoplasts were overlaid with HYG-containing medium for transient selection of Cas9/tRNA-sgRNA-expressing cells. Representative transformation plates after 9 weeks of incubation are shown. For summary of the results of three independent experiments see Supplementary Figure 3B. **(C)** Isolation of partial loss-of-functions mutants of PHS1 from editing approaches. Editing of *phs1* with and without *phs1*-oligo1 in the *pks1-* background resulted in several colonies with a lighter orange pigmentation. Three of them, O1-O3 were isolated and studied. Melanin-deficient strains on the left were cultivated for seven days on SDNG (pink: *pks1-*, white: *pks1-/phs1*-). Sequencing of the *phs*1^PS1^-spanning region revealed deletions of 9 bp in O1, 12 bp in O2, and 23 bp in O3 (Supplementary Figure 4), resulting in PHS1 enzymes lacking three, four or eight amino acids in the N-terminal lycopene cyclase domain.

Short-homology (SH) fragments for replacement of gene of interests (*goi*) by resistance cassettes were amplified by PCR using pNDH-OGG (hygR), pNDN-OGG (natR) (Schumacher, 2012), pNDG-OGG (genR), pNDP-OGG (baR), or pNDS-OGG (suR) (this study, Figure 2) as template and primers *goi*-SH5F (binding in T*niaD*) and *goi*-SH3R (binding in P*oliC*) containing ~75-bp-long 5′ overhangs homologous to target genomic regions. For replacement of genes by a resistance cassette fused to an expression cassette, the constructs were amplified by PCR with primers T*xxx*-*goi*-SH5F (binding in T*niaD*/T*niiA* of the resistance cassette) and T*xxx*-*goi*-SH3R (binding in T*trpC/*T*gluc/*T*tub1* of the expression cassette) containing ~75-bp-long homologous sequences as 5′ overhangs. Per transformation approach 10–15 μl of the PCR were used. Constructs with long-homology (LH) fragments were isolated from cloned plasmids *via* digestion (2 μg DNA per transformation approach). Protospacers (PSs) for CRISPR/Cas9 were identified with the CRISPR site finder of Geneious Prime® 2021 (Biomatters Ltd.). AMA1-bearing vectors for mediating the extrachromosomal expression of Cas9 and target-specific sgRNAs (liberation from transcripts by attached ribozymes or endogenous RNases) were cloned as previously described (Nødvig et al., 2015, 2018; Wenderoth et al., 2017) (Supplementary Figure 2) and transformed in the circular form (2 μg of each plasmid per transformation approach). Transformations carried out with different combinations of CRISPR/Cas9 plasmids and donor DNA are summarized in Supplementary Table 4. PEG-mediated transformation of protoplasts was performed as described previously (Noack-Schönmann et al., 2014a; Voigt et al., 2020), but an osmotically stabilized synthetic-defined medium lacking amino acids [KTM, *Knufia* transformation medium: SDNG supplemented with 0.49 M (16.8%) sucrose] was used for regeneration of protoplasts. Distributed protoplasts (5 × 10^5^ protoplasts on 20 ml of KTM per Petri dish) were overlaid after 24 h with 5 ml of warm KTM supplemented with 0.4% (w/v) agar and 250 μg ml^−1^ HYG (final conc. 50 μg ml^−1^), 25 μg ml^−1^ NTC (final conc. 5 μg ml^−1^), 500 μg ml^−1^ G418 (final conc. 100 μg ml^−1^), 200 μg ml^−1^ GFS (final conc. 40 μg ml^−1^) or 375 μg ml^−1^ CME (final conc. 75 μg ml^−1^). Putative transformants were transferred after 2–3 weeks to MEA containing 25 μg ml^−1^ of HYG and/or 5 μg ml^−1^ of NTC, or to SDNG containing 100 μg ml^−1^ of G418, 40 μg ml^−1^ of GFS or 75 μg ml^−1^ of CME. Targeted integration of replacement and expression constructs was detected by diagnostic PCRs with primers binding up- and downstream of the integration sites and within the transformed constructs. Gene editing events were detected by amplicon sequencing.

### Detection of Fluorescence

For measuring GFP fluorescence with a spectral photometer, the P*oi::gfp* strains were cultivated for 7 days in 20 ml of MEB at 25°C and 100 rpm. Cultures were dispersed in grinding jars with eight steel beads (5 mm) by using a TissueLyser™ (Retsch GmbH, Haan, GER; 10 min at 30 Hz). Cells were washed twice with PBS and finally suspended in 5 ml of PBS. Cell titers were determined using a Thoma cell counting chamber. Ninety-six-well plates were prepared with 1 × 10^9^ cells in 200 μl PBS per well. GFP fluorescence (excitation 485/20, emission 528/20) was quantified with a Synergy HT microplate spectrophotometer and the Gen5™ software (BioTek; Agilent Technologies, Inc., Santa Clara, CA, USA). Fluorescence and light microscopy of cells suspended in PBS were performed with a Zeiss AxioImager M2m microscope. Cells were resuspended in PBS-based solution in a final concentration of 2 μg ml^−1^ of 4′,6-diamidino-2-phenylindole (DAPI; Sigma-Aldrich) (1 mg ml^−1^ H_2_O) for staining the nuclei prior to microscopy. GFP fluorescence was examined with filter set 38 HE [excitation BP 470/40 (HE), beam splitter FT 495 (HE), emission BP 525/50 (HE)], mCherry fluorescence using filter set 14 (excitation BP 510-560, beam splitter FT 580, emission LP 590) and DAPI fluorescence using filter set 49 (excitation G 365, beam splitter FT 395, emission BP 445/50). Images were captured with a Zeiss AxioCam 503 mono camera and analyzed using the ZEN 2 (blue edition) v3.2.0.0000 software package (Carl Zeiss AG, Jena, GER).

## Results

### Plasmid-Based CRISPR/Cas9 for the Introduction of Multiple Mutations

In genome editing, the term “multiplexing” refers to the ability to edit more than one genomic site in an organism (Schuster and Kahmann, 2019). Multiplex CRISPR/Cas9 may include using a single sgRNA targeting the conserved region of related genes or using several sgRNAs targeting unrelated genes. In *K. petricola*, CRISPR/Cas9-mediated simultaneous replacement of *pks1* and *phs1* resulting in white mutants was achieved using two Cas9/sgRNA-delivering plasmids (pAMA/ribo−*pks*1^PS2^, pAMA/ribo−*phs*1^PS1^) or two RNPs (RNP-*pks*1^PS2^, RNP-*phs*1^PS1^) which were co-transformed with resistance cassette-containing replacement fragments (Voigt et al., 2020). For increasing the efficiency of simultaneous mutations with the perspective for generation of triple and quadruple mutants, an alternative for the expression of sgRNAs was quested. Considering that AMA1-bearing plasmids work in *K. petricola* when a sgRNA is released by ribozymes from a mRNA transcript (pAMA/ribozyme), the functionality of plasmid-based expression of single/multiple sgRNAs from tRNA splicing cassettes according to Nødvig et al. was assessed. In this system (pAMA/tRNA), a tRNA-sgRNA-tRNA cassette is flanked by the U3 regulatory sequences of *Aspergillus fumigatus* for mediating the transcription by the RNA polymerase III and subsequent release of sgRNA(s) from the RNA transcript by the endogenous tRNA maturation machinery (Nødvig et al., 2018) (Supplementary Figure 1).

To evaluate the efficiency in *K. petricola, pks1*, and *phs1* were targeted by using the same protospacers (*pks*1^PS2^, *phs*1^PS1^) and repair templates as in the previous study. For testing the expression of one and two sgRNAs from a tRNA splicing cassette, the plasmids pAMA/tRNA-*pks*1^PS2^, pAMA/tRNA-*phs*1^PS1^ and pAMA/tRNA-*pks*1^PS2^-*phs*1^PS1^ were cloned (Supplementary Table 3). For gene replacement, wild-type or *pks1*- protoplasts were co-transformed with the different Cas9/sgRNA-delivering plasmids (pAMA/tRNA *vs*. pAMA/ribozyme) and appropriate replacement fragments (Δ*pks1* [N], Δ*phs1* [H]) conferring resistance to nourseothricin (NTC) and hygromycin (HYG), respectively (Supplementary Table 4; Figure 2A). Single sgRNA expressed from tRNA and ribozyme cassettes resulted in similar numbers of differentially pigmented resistant colonies, i.e., pink (transformation of WT with sgRNA^*pks*1^ + Δ*pks1* [N], upper row) and white (transformation of *pks1-* with sgRNA^*phs*1^ + Δ*phs1* [H], middle row). However, the use of a single plasmid for the expression of two sgRNAs (pAMA/tRNA-*pks*1^PS2^-*phs*1^PS1^) in combination with both replacement fragments resulted in a greater number of white *natR hygR* colonies compared to the control approach with the same sgRNAs expressed from two different pAMA/ribozyme plasmids (Supplementary Figure 3).

In a second experiment, WT and *pks1*- protoplasts were transformed with the three pAMA/tRNA plasmids alone for random gene editing or together with the corresponding 80-bp-long single stranded DNA oligonucleotides *pks1*-oligo2.7 or *phs1*-oligo1 as repair templates for targeted gene editing (Supplementary Table 4; Figure 2B). Protoplasts were overlaid with medium containing HYG for transient selection of pAMA-containing cells potentially becoming edited strains. From the transformation plates shown, it is evident that the addition of oligonucleotides overlapping the Cas9 cutting sites considerably increased the number of differentially pigmented colonies which is in accordance with previous experiments with ribozyme-released sgRNAs. Furthermore, the rate of double gene editing (white mutants) by co-transformation of pAMA/tRNA-*pks*1^PS2^-*phs*1^PS1^ and the two oligonucleotides was increased [53 vs. ~40% for expression of the two sgRNA from two plasmids (Voigt et al., 2020)]. Double gene editing events in twelve white mutants (W1-12) were analyzed by amplicon sequencing of the DSB/PS-spanning regions of *pks1* and *phs1* (Supplementary Figures 1, 4). Accordingly, two different mutations causing in-frame deletions of 3 bp in *pks1* were identified resulting in PKS1^Δ*L*118^ (in seven mutants; mutation was present in used *pks1*-oligo2.7) or PKS1^Δ*L*119^ (five mutants). All twelve mutants contained the same 2-bp-mutation (present in used *phs1*-oligo1) in *phs1* resulting in a premature stop codon and a truncated protein (PHS1^1−63aa^).

Interestingly, the transformation of melanin-deficient *pks1-* protoplasts with pAMA/tRNA-*phs*1^PS1^ resulted in several colonies with an orange pigmentation instead of the expected white phenotype (Figures 2A,B). Three of them (O1-3) were studied by amplicon sequencing revealing that they achieved different deletions in the 5′ region of *phs1*: O1 contains an in-frame deletion of 9 bp resulting in a 3-aa-long deletion plus one aa exchange (PHS1^Δ63−65, *V*66*L*^), O2 an in-frame deletion of 12 bp resulting in a 4-aa-long deletion (PHS1^Δ63−66^), and O3 a deletion of 23 bp resulting in a 8-aa-long deletion plus one aa exchange (PHS1^Δ60−67, *G*68*S*^) (Figure 2C; Supplementary Figures 1, 4). Considering the color of the mutants indicating that carotenoids are still produced, and that the identified deletions retain the reading frame, it appears likely that the three PHS1 variants maintained the function to catalyze the formation of phytoene (first step in carotenoid synthesis) *via* the C-terminal domain but are affected in the cyclization of carotenoids *via* the N-terminal lycopene cyclase domain.

Taken together, expression of sgRNA(s) from pAMA/tRNA plasmids works well for gene editing and gene replacement approaches, indicating that the U3 regulatory sequences from *A. fumigatus* mediate moderate transcription of the tRNA-sgRNA-tRNA cassettes by the RNA polymerase III in *K. petricola*. Double mutants are obtained more easily, and the generation of triple and quadruple mutants becomes a realistic objective. Because of the eligibility and the flexibility of this system—one primer pair per sgRNA allows for assembling of plasmids with various combination of sgRNAs—it will replace the pAMA/ribozyme strategy in the long term.

### Additional Antibiotic/Drug Resistance Selection Marker for Mutant Generation

The CRISPR/Cas9 technique can be applied for mutating multiple genes without introducing the equal number of selection markers; however, replacing genes by resistance cassettes facilitates the screening for all desired mutations by selection and diagnostic PCR. Moreover, replacement approaches eliminate the entire gene and yield independent mutants with identical genotypes. Formerly, two selection systems, i.e., hygR and natR, were available in *K. petricola*. First triple mutants were generated by replacing genes in the selection marker-free pigment mutant *pks1-/phs1-* that derived from a gene editing approach (not shown). Nevertheless, additional selection systems are beneficial for various approaches. Further frequently used selection marker systems in fungi are bleR [phleomycin (PHLEO)/*ble*], genR [geneticin (G418)/*nptII*], baR [glufosinate (GFS)/*bar*], suR [chlorimuron ethyl (CME)/*sur*] and fenR [fenhexamid (FEN)/*erg*27^*^] (Sweigard et al., 1997; Cohrs et al., 2017; Lichius et al., 2020) (Table 1). The usage of bleR was excluded as phleomycin introduces DSBs in DNA and thus may lead to additional undesired mutations. A first rapid assay showed that growth of *K. petricola* is prevented in amino acid-lacking SDNG medium supplemented with geneticin (G418), glufosinate ammonium (GFS) and chlorimuron ethyl (CME), but is not affected by fenhexamid (data not shown). As a result, genR, baR, and suR were evaluated as putative new selection systems.

**Table 1:** Five resistance selection marker systems are available for *K. petricola*.

	**Toxic compound**	**Resistance gene, protein description**	***K. petricola***
hygR	Hygromycin B (HYG)	*Escherichia coli hph—*hygromycin B phosphotransferase	Applicable^a^
natR	Nourseothricin (NTC)	*Streptomyces noursei nat1—*nourseothricin N-acetyltransferase	Applicable^a^
bleR	Phleomycin (PHLEO)	*Streptoalloteichus hindustanus ble—*bleomycin-binding protein	n/a^b^
fenR	Fenhexamid (FEN)	*Fusarium fujikuroi erg27—*insensitive 3-keto sterol reductase	n/a^c^
genR	Geneticin (G418)	*Escherichia coli nptII—*neomycin phosphotransferase 3'[II]	Applicable^d^
baR	Glufosinate (GFS)	*Streptomyces hygroscopicus bar—*phosphinothricin acetyltransferase	Applicable^d^
suR	Chlorimuron ethyl (CME)	*Magnaporthe oryzae sur—*insensitive acetolactate synthase	Applicable^d^

a *Voigt et al. (2020)*.

b *Not tested because of high mutagenic potential of bleomycin/phleomycin*.

c *K. petricola ERG27 is insensitive to fenhexamid (data not shown)*.

d *This study*.

**Figure 3:**
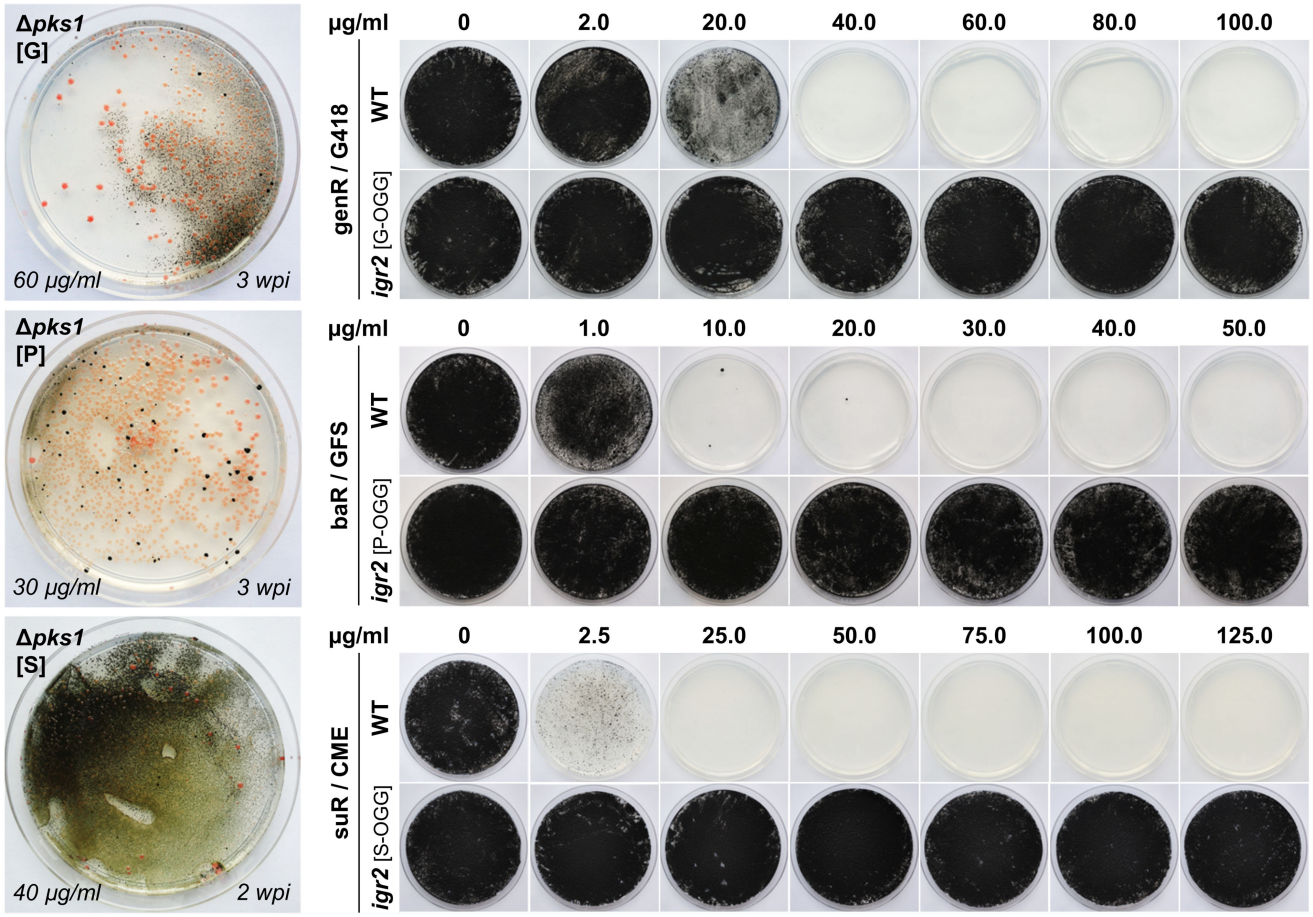
Implementation of genR, baR and suR selection marker systems in *K. petricola*. Pink Δ*pks1* mutants were generated by co-transformation of WT:A95 protoplasts with pAMA/ribo-*pks*1^PS2^ and Δ*pks1* fragments containing genR (G), baR (P), and suR (S) cassettes (Supplementary Table 4). Pictures of transformation plates with pink colonies and black background growth after 2–3 weeks of incubation are shown on the left. DNA uptake and homologous integration were tested for six pink mutants per selection marker by diagnostic PCR (Supplementary Figure 7). Black strains *igr2* (G/P/S-OGG) containing a GFP expression cassette fused to a genR, baR or suR cassette were generated by co-transformation of WT:A95 protoplasts with pAMA/ribo-*igr*2^PS1^ and expression constructs amplified with primers containing 5′ overhangs to *igr2*. Targeted insertion was examined in six strains per construct (Supplementary Figure 8). For evaluating the sensitivities of the wild type compared to those of the resistance gene-expressing strains, 5 × 10^5^ cells were spread onto solidified SDNG supplemented with the indicated concentrations of the selective agents geneticin (G418), glufosinate ammonium (GFS) or chlorimuron ethyl (CME) and incubated for 3 weeks in darkness at 25°C.

In a previous study, primers (*goi*-SH5F, *goi*-SH3R) were designed allowing for the amplification of the hygR [H] and natR [N] cassettes from pNDH-OGG and pNDN-OGG, respectively (Voigt et al., 2020). These plasmids derive from the pNXR-XXX series which have all the same modular structure (Schumacher, 2012) (Supplementary Figures 5, 6). The 3′ ends of the primers are standardized and bind to the identical regulatory sequences of the resistance cassettes, while the 5′ overhangs are flexible and represent the ~75-bp-long 5′- and 3′-noncoding sequences of the *goi* (SH sequences). Other compatible templates are pNDB-OGG (bleR) and pNDF-OCT (fenR) (Schumacher, 2012; Cohrs et al., 2017) (Supplementary Figure 5). Accordingly, plasmids with compatible genR, baR and suR cassettes were cloned. The resistance genes *nptII, bar*, and *sur* were amplified from pKS-Gen, pCB1524 or pCB1532 (Sweigard et al., 1997; Bluhm et al., 2008; McCluskey et al., 2010) with primers generating overlaps with P*trpC* or T*niaD* and obtained amplicons were assembled with the pND–OGG backbone. Resulting pNDG-OGG (genR), pNDP-OGG (baR) and pNDS-OGG (suR) (Supplementary Table 3) were used as templates for the PCR-based generation of Δ*pks1* fragments with genR [G], baR [P], or suR [S] cassettes (Table 2). Because GFS and CME depend on an amino acid-free medium for preventing growth, the complex osmotically stabilized transformation medium (MEAS) was replaced by synthetic-defined osmotically stabilized SDNG (KTM). Protoplasts of WT:A95 were co-transformed with pAMA/ribo-*pks*1^PS2^ and Δ*pks1* [G], Δ*pks1* [P], or Δ*pks1* [S] fragments (Supplementary Table 4), and overlaid with KTM-TOP containing G418, GFS and CME in different concentrations. Pink colonies appeared after 1–2 weeks on the top and could be clearly separated from the black cells growing in the lower layer (background) (Figure 3). By adjusting the concentrations in following experiments, background growth was reduced by using G418 and GFS in higher concentrations. However, increased concentrations of CME did not have the same effect likely because of the relatively short half-life of the compound (Choudhury and Dureja, 1996). Thus, putative transformants must be isolated from the transformation plates as soon as possible. Notably, pink *genR* colonies were found only when KTM (and not MEAS) was used. As usage of KTM also results in faster protoplast recovery which shortens the period until top-grown transformants can be transferred, KTM is used in the meantime for all transformations independent of the selective agent. Twelve pink colonies per selection marker were transferred to SDNG supplemented with the appropriate selective agent. All mutants displayed moderate growth, thus six mutants per selection marker system were examined by diagnostic PCR (Supplementary Figure 7). The detection of the expected amplicons for HR events at 5′ and 3′ of *pks1* for most of the mutants confirmed the stable integration of the different resistance cassettes into the genome of *K. petricola* for conferring resistance.

**Table 2:** Generation of replacement fragments with resistance cassettes by single-step PCR.

**R**		**Resistance cassette**	**Size**	**Plasmid**	**Size**	**References**	**Size of Δ*goi* SH**
H	hygR	P*trpC*::*hph*::T*niaD*	1.757 kb	pNDH-OGG	11.094 kb	Schumacher, 2012	1.941 kb
N	natR	P*trpC*::*nat1*::T*niaD*	1.295 kb	pNDN-OGG	10.626 kb	Schumacher, 2012	1.473 kb^*^
B	bleR	P*trpC*::*ble*::T*niaD*	1.129 kb	pNDB-OGG	10.434 kb	Schumacher, 2012	1.279 kb^*^
F	fenR	P*trpC*::*fferg27*::T*niaD*	2.221 kb	pNDF-OCT	11.755 kb	Cohrs et al., 2017	2.405 kb
G	genR	P*trpC*::*nptII*::T*niaD*	1.520 kb	pNDG-OGG	10.851 kb	This study	1.698 kb
P	baR	P*trpC*::*bar*::T*niaD*	1.277 kb	pNDP-OGG	10.608 kb	This study	1.455 kb^*^
S	suR	P*trpC*::*sur*::T*niaD*	3.212 kb	pNDS-OGG	12.543 kb	This study	3.390 kb

*Resistance (R) cassettes flanked by short homologous (SH) sequences of ~75 bp are generated by PCR using primers (goi-SH5F × goi-SH3R) binding in conserved regions of the resistance cassettes (TniaD, PoliC) and containing 5′ overhangs homologous to the 5′- and 3′-noncoding regions of the gene of interest (goi). The seven listed plasmids of the pNDR-OGG series are used as templates (Supplementary Figures 5, 6). Sizes of the Δgoi SH fragments are indicated when primers contain homologous 5′ overhangs of 75 bp*.

* *nat1, ble, and bar have high GC contents, thus special measures for the amplification of the GC-rich sequences must be taken*.

In a second experiment, expression constructs comprising the three different resistance cassettes linked to a *gfp* cassette should be integrated in a melanin-containing wild-type-like background. Therefore, the cloned pNDX-OGG plasmids were used as templates for amplifying the expression constructs with primers binding in T*niaD* (resistance cassette) and T*gluc* (expression cassette) and providing 5′ overhangs homologous to intergenic region 2 (*igr2*, see Section Identification of Two Neutral Genome Loci for Targeted Insertion of Expression Constructs). For generating resistant black mutants, WT:A95 protoplasts were co-transformed with the SH fragments and pAMA/ribo-*igr*2^PS1^ (Supplementary Table 4). Many black *genR, baR* and *suR* transformants were obtained. Six transformants per selection system were studied by diagnostic PCR detecting the correct insertion in most of the transformants (Supplementary Figure 8A). Finally, the growth phenotypes of resistant strains and WT:A95 were examined on solidified SDNG with different concentrations of G418, GFS, or CME, using two different modes of inoculation (Figure 3; Supplementary Figure 8B). In the first method, 5 × 10^5^ cells per Petri dish were distributed with glass beads, which corresponds to the number of protoplasts per Petri dish used for transformation. The second inoculation method simulates the transfer of transformants to fresh selection medium for secondary selection by inoculating an undefined number of cells from 1-week-old surface-grown colonies using an inoculation loop. Generally, higher concentrations of the selective agents are needed to prevent growth of wild type A95, when undefined numbers of cells are streaked out on solidified medium. Based on these results (inhibitory concentrations) as well as observations from transformation approaches, the most suitable concentrations for primary and secondary transformant selection were defined (see Section Materials and Methods).

The performed experiments demonstrated the applicability of the three selection marker systems for transforming protoplasts of *K. petricola*. The set of compatible plasmids was expanded allowing for the PCR-based generation of gene replacement fragments with seven different resistance cassettes (five usable in *K. petricola*) by using a single pair of ~100-bp-long primers, and for allowing the PCR-based generation of expression constructs for different applications including knock-in in native gene loci (Supplementary Figure 6) or other genomic loci as described in the following.

### Application of Black-Pink Screening for Promoter Studies

Promoters are the basis for gene expression, though the nature of the gene and the terminator may significantly influence the stability and half-life of the transcript and the protein. Appropriate promoter activities are essential for most molecular tools, e.g., for driving expression of endogenous or foreign genes including reporter genes. Few promoters are known to mediate high and constitutive gene expression in several but not all fungi. Known functional promoters in *K. petricola* derive from *A. nidulans* and are: P*trpC* used for expression of resistance cassettes, P*tef1* used for expression of *cas9* from AMA1-bearing plasmids, and P*gpdA* and P*oliC* used for expression of fluorescent reporter gene constructs (Voigt et al., 2020) (Supplementary Table 3). However, in the same study inconclusive results were obtained for P*act1* from *Botrytis cinerea* that allows for moderate expression of *gfp* in different fungi. To learn more about the performance of already used promoters and to identify those enabling controllable gene expression, experiments were set-up to compare promoter (of interest, P*oi*) activities using *gfp* as reporter gene for quantifying GFP fluorescence and expressing these P*oi::gfp* constructs in identical genetic backgrounds. For taking advantage of the pigmentation phenotypes that are already visible on the transformation plates due to the haploid and uninucleate nature of *K. petricola*, the *pks1* locus was chosen for targeted integration (knock-in) of the expression constructs. In addition, melanin-free strains appeared more favorable for quantifying GFP fluorescence. Six different promoters were included in the study (Table 3); the previously mentioned P*oliC* and P*gpdA* from *A. nidulans*, P*act1* from *B. cinerea* and P*gln1* from *Fusarium fujikuroi* as constitutive promoters, as well as P*niaD* from *A. nidulans* and P*gal1* from *K. petricola* (Supplementary Sequence 1) as candidates for controllable promoters. At least in some fungi, promoters of nitrogen assimilation and galactose metabolism genes underlie positive and negative regulation by substrate induction and catabolite repression, respectively (Krappmann and Braus, 2005; Christensen et al., 2011).

**Table 3:** Heterologous promoters used and/or tested in *K. petricola*.

**Name**		**Size**	**Gene/encoded protein (function/pathway)**	**Species**
P*trpC*		0.357 kb	Anthranilate synthase component 2 (tryptophan synthesis)	*Aspergillus nidulans*
P*tef1*		0.886 kb	Translation elongation factor EF-1 alpha subunit (translation)	*Aspergillus nidulans*
P*ddr48*		1.021 kb	DNA damage-responsive protein (stress response)	*Fusarium fujikuroi*
P*oliC*	O	0.845 kb	Subunit 9 of F1F0-ATPase complex (ATP synthesis)	*Aspergillus nidulans*
P*gpdA*	G	0.873 kb	Glyceraldehyde-3-P dehydrogenase (glucose catabolism)	*Aspergillus nidulans*
P*act1*	A	0.850 kb	Actin (cytoskeleton)	*Botrytis cinerea*
P*gln1*	Q	0.889 kb	Glutamine synthetase (ammonia assimilation)	*Fusarium fujikuroi*
P*niaD*	N	1.266 kb	Nitrate reductase (nitrate assimilation)	*Aspergillus nidulans*
P*gal1*	L	0.659 kb	Galactokinase (galactose catabolism)	*Knufia petricola*

**Figure 4:**
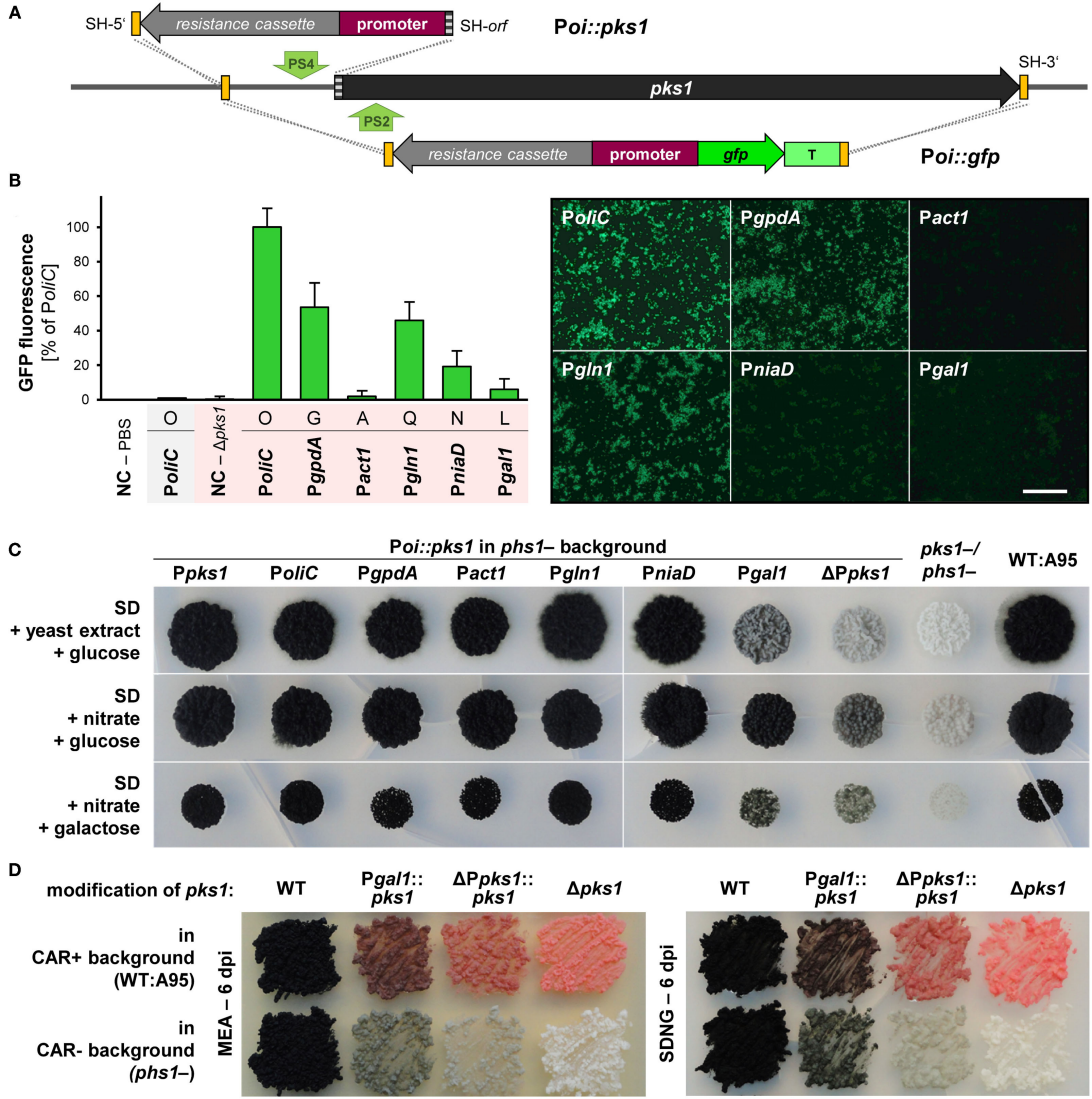
Promoter studies using GFP/fluorescence and PKS1/pigmentation as reporters in *K. petricola*. **(A)** Strategies for the targeted integration of P*oi::gfp* and P*oi*-only constructs at the *pks1* locus. The promoter and open reading frame of *pks1* were replaced by constructs containing *gfp* under control of six different promoters (P*oi*). This allowed for the isolation of correct (pink) strains (black-pink screening). For using pigmentation as reporter, the *pks1* promoter (1-kb-long sequence upstream of ATG) was replaced by constructs containing the six different promoters. All constructs comprise a hygR cassette for selection. Constructs were amplified from different plasmids with primers containing SH sequences of the non-coding or coding regions of *pks1* as 5′ overhangs (yellow/gray boxes). Green arrows highlight the Cas9 cutting sites (PS2, PS4). Primers and strategies for detection of HR events (dashed lines) are indicated in Supplementary Figures 9, 10. **(B)** Expression of *gfp* from P*oliC* and P*gpdA* but not from P*act1* result in considerable GFP fluorescence. The pink P*oi*::*gfp* strains were generated through co-transformation of WT:A95 protoplasts with pAMA/ribo-*pks*1^PS2^ and the six different P*oi*::*gfp* constructs with homologous sequences to the 5′- and 3′-noncoding of *pks1* (Supplementary Table 4; Supplementary Figure 9). Three strains per construct were cultivated for 7 days in liquid culture. GFP fluorescence was quantified by a microplate spectrophotometer (10^9^ cells per well). Mean values and standard deviations were calculated from three strains per construct and three wells per strain; except for P*oliC::gfp* in WT background (shaded gray; no standard deviation given as duplicates only). Negative controls (NC) were PBS only and cells of the GFP-free pink strain Δ*pks1* (left). Fluorescence patterns of the same cell suspensions observed by fluorescence microscopy with exposure times of 400 ms (right). Scale bar−300 μm. **(C)** Almost all promoters efficiently drive *pks1* expression resulting in pigmented colonies. Strains in which *pks1* is controlled by the P*oi* were generated by co-transformation of carotenoid-deficient *phs1-* protoplasts with pAMA/ribo-*pks*1^PS4^ and the six different hygR-promoter constructs or with hygR only (background control) as specified in Supplementary Table 4 and Supplementary Figure 10. 10^4^ cells of the P*oi::pks1* strains, background control (ΔP*pks1*), recipient strain *phs1-* (P*pks1*), pigment-deficient *pks1-*/*phs1-* and WT:A95 were dropped on solidified SD medium supplemented with glucose or galactose as carbon sources, and nitrate or yeast extract as nitrogen sources and incubated for 25 days in darkness. **(D)** The replacement of the endogenous *pks1* promoter leads to different shades of melanin. Strains with reduced melanin formation in a carotenoid-containing background were generated by co-transformation of WT:A95 protoplasts with pAMA/ribo-*pks*1^PS4^ and the two hygR-promoter constructs resulting in ΔP*pks1::pks1* and *Pgal1::pks1*. Cells of the indicated strains were dispersed on solidified MEA and SDNG and incubated for 6 days in darkness.

Expression constructs for the first four promoters were available (Schumacher, 2012), compatible constructs for P*niaD* and P*gal1* were cloned (Supplementary Table 3). As the application of CRISPR/Cas9 mediates efficient HR with short homologous (SH) sequences and the used plasmids/constructs comprise identical modular structures, the six different expression constructs could be amplified from the six plasmids with (four) primers binding in T*niaD*/T*niiA* of the resistance cassette and in T*gluc*/T*trpC* of the expression cassette, and containing ~75-bp-long 5′ overhangs homologous to noncoding regions of *pks1* (Figure 4A; Supplementary Figure 5). The co-transformation of WT:A95 protoplasts with pAMA/ribo-*pks*1^PS2^ and the six P*oi::gfp* expression constructs (Supplementary Table 4) resulted in plenty of pink *hygR* transformants (data not shown). The correct integration event at *pks1* that left the flanking sequences intact, was exemplarily examined for 16 strains of P*niaD::gfp* and P*gal1::gfp* by diagnostic PCR as described in Supplementary Figure 9 (data not shown). In fact, the expected amplicons for HR at 5′ and 3′ of *pks1*/the Cas9 cutting site were obtained in all transformants demonstrating the suitability of the applied black-pink screening for the fast identification of expression construct-containing transformants. For further analyses, three strains per P*oi::gfp* construct were chosen. Cell suspensions from seven-day-old liquid cultures were prepared, and different numbers of cells in 200 μl PBS were transferred to microtiter plates for detecting GFP fluorescence in a microplate spectrophotometer. This pilot experiment revealed that 10^9^ melanin-free GFP-expressing cells per well resulted in the most reliable data while no fluorescence could be detected for black GFP-expressing (P*oliC::gfp*) cells by this set-up. The experiment, i.e., cultivation of the pink P*oi::gfp* strains followed by the detection of fluorescence was repeated twice yielding the same results (Figure 4B). Thus, P*oliC* (100%) mediated the highest GFP fluorescence followed by P*gpdA* (54%) and P*gln1* (46%). Very low levels of fluorescence were detected for P*niaD* (19%), P*gal1* (6%), and P*act1* (2%). Overall, these data are in accordance with the observations made by fluorescence microscopy when images with identical exposure times were captured.

Inspired by the first promoter studies using *gfp* as reporter and *pks1* as integration site, another approach was taken using the endogenous *pks1* (pigmentation) as a reporter. The same plasmids were used as templates but only the resistance cassette with the P*oi* was amplified by PCR with primers attaching 75-bp-long homologous sequences to the 5′-noncoding region (as before) or the 5′ end of *pks1* to the expression constructs (Figure 4A; Supplementary Figure 10). Thus, by co-transformation of these amplicons with pAMA/ribo-*pks*1^PS4^ for inserting a DSB in the 5′-noncoding region of *pks1* (Supplementary Table 4), the endogenous promoter was replaced by the P*oi* resulting in P*oi::pks1*. As “background” control, P*pks1* (1.0 kb) was replaced by the hygR cassette only (ΔP*pks1*). To obtain clearer nuances of melanin, the constructs were introduced into the carotenoid-free *phs1-* mutant. Obtained *hygR* transformants were checked by diagnostic PCR, and for two transformants per promoter, the 3′ region comprising the P*oi-pks1* transitions were amplified and sequenced to confirm the absence of mutations in *pks1*. Cell suspensions of P*oi::pks1* and control strains were dropped onto different solidified media including SDYG (control; Figure 4C) and media containing nitrate or galactose as single nitrogen or carbon source to induce P*niaD* and P*gal1*, respectively. However, no obvious differences to the control plates were found (Figure 4C). The experiment revealed that deletion of 1 kb of the 5′-noncoding region (considered promoter) of *pks1* did not eliminate *pks1* expression by leading to gray colonies. In this expression system, P*gal1* showed the lowest activity by resulting in colonies pigmented similarly to the background control. All other P*oi::pks1* fusions led to fully melanized colonies not allowing for detection of gradual promoter activities. The mutations P*gal1::pks1* and ΔP*pks1::pks1* were introduced into the wild-type background as well for yielding mutants with reduced melanin contents for further phenotypic characterization (Supplementary Tables 1, 2; Figure 4D).

In sum, the promoter studies clarified that P*oliC* or P*gpdA* (and not P*act1*) must be used for appropriate expression of fluorescent reporter genes in *K. petricola*. No controllable promoters could be identified in this pilot study; however, the established protocols for generating (melanin-free) strains with identical genetic backgrounds and studying promoter activities by quantification of GFP fluorescence will allow screening further candidate promoters in the future. Finally, the knock-in of expression constructs by replacing *pks1* enables the rapid identification of correct (pink) transformants (black-pink screening).

### Application of Black-White Screening for Protein-Protein Interaction Studies

To elucidate biological processes in detail, protein localization and protein-protein interaction studies *in vivo* might be crucial and are more favorable than performing interaction studies in heterologous hosts. Both co-localization and bimolecular fluorescence complementation (BiFC) studies require the integration of two expression constructs into the genome, and the two-step generation of such strains can be laborious and time-consuming.

The BiFC technology is based on the association of non-fluorescent protein fragments, e.g., split GFP or YFP that are fused to proteins of interest and expressed in living cells. A physical interaction of the proteins of interest will bring the reporter fragments in proximity, allowing the formation of the native reporter protein and the emission of its fluorescent signal. Therefore, BiFC studies identify both the localization and interaction partners of the protein of interest in different organisms including fungi (Kerppola, 2008). Co-localization studies refer to a spatial overlap between two (or more) different fluorescently labeled proteins with varying emission wavelengths. A suitable pair of fluorescent proteins represents GFP and DsRED/mCherry. In contrast to BiFC, these approaches are not suitable to detect protein-protein interactions, but they can be extremely helpful to locate the protein of interest to a specific cellular compartment by avoiding the application of fluorescent dyes.

**Figure 5:**
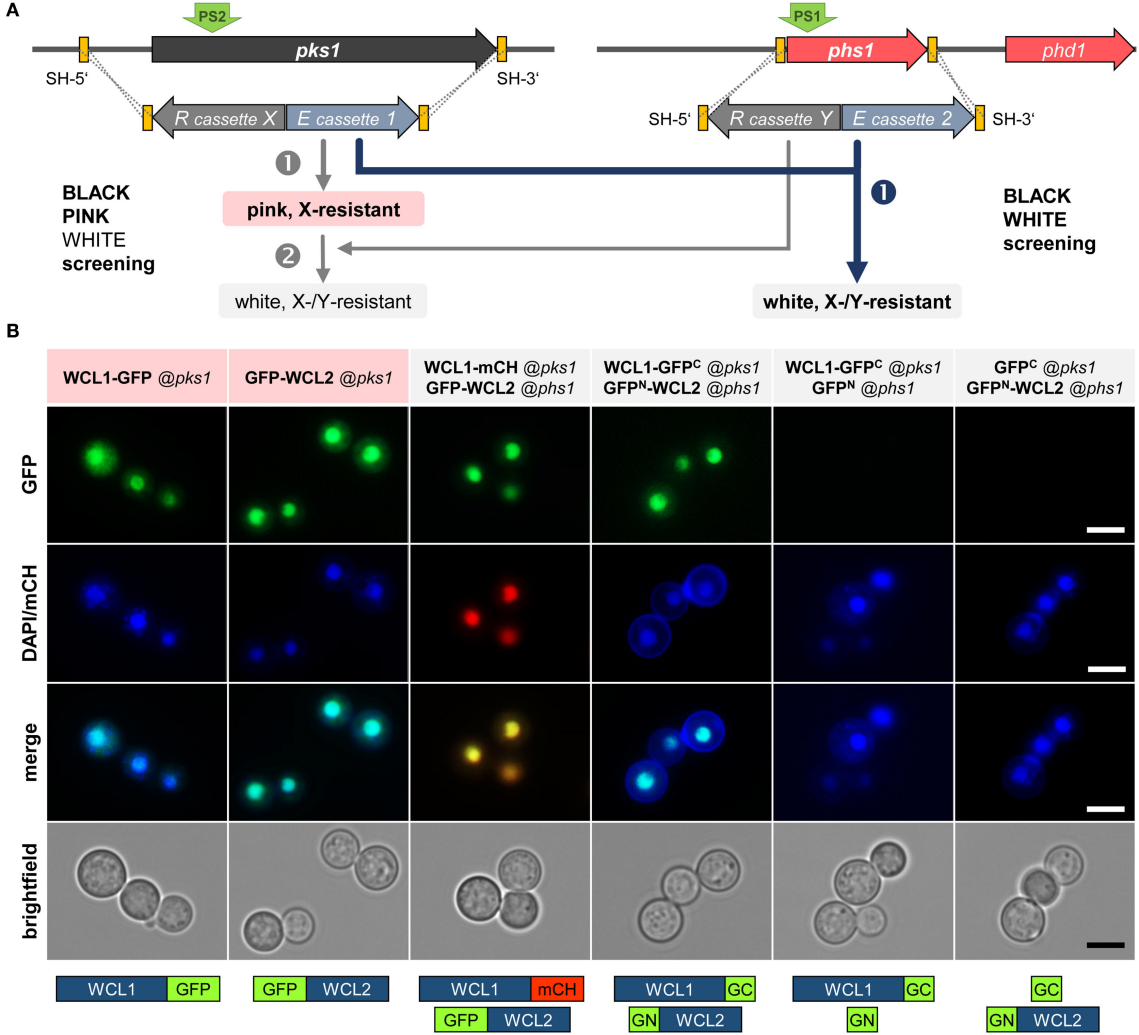
Physical interaction of the *K. petricola* White Collar transcription factors in the nuclei. **(A)** Mutation of essential genes for pigment synthesis allows for a two color-based screening of transformants. The color screening in combination with the introduced resistance (R) cassette X/Y enables a rapid identification of transformants carrying the expression (E) constructs 1/2. The integration of two different constructs by replacing *pks1* and *phs1* can be done stepwise (black-pink + pink-white screening; gray arrows on the left) or in one step (black-white screening; blue arrow on the right). Yellow boxes indicate the SH sequences for targeted integration by HR (dashed lines). For transformation, the constructs are amplified by PCR with primers having target-specific sequences as 5′-overhangs. The green arrows (PS2/PS1) highlight the Cas9 cutting sites. **(B)** The White Collar transcription factors WCL1 and WCL2 co-localize and interact in the nuclei. The coding regions of *wcl1* and *wcl2* were amplified from DNA of *K. petricola* and fused to full-length reporters and *gfp* fragments (*gfpN, gfpC*) in basal cloning vectors (Schumacher, 2012) (Supplementary Table 3). The expression constructs were amplified with primers containing the sequences homologous to the 5′ and 3′-noncoding regions of *pks1* or *phs1*. WT:A95 protoplasts were co-transformed with expression constructs and pAMA/ribo-*pks*1^PS2^ for single integrations or with pAMA/tRNA-*pks*1^PS2^-*phs*1^PS1^ for double integrations (Supplementary Table 4). Cell suspensions of pink *hygR* and white *hygR/natR* transformants were examined by fluorescence microscopy. Localization/positive controls (lanes 1–2), co-localization (3), bimolecular fluorescence complementation (BiFC) (4), negative controls for BiFC (5–6). Scale bars–5 μm.

With the aim of demonstrating the functionality of BiFC studies in *K. petricola* using available plasmids with split-GFP constructs of *B. cinerea* codon-optimized *gfp* (Leroch et al., 2011; Schumacher, 2012), a method was quested allowing for the easy and fast generation of strains containing two expression constructs. Considering the results for double editing by plasmid-based multiplex CRISPR/Cas9 and the well-working black-pink screening, the color-based screening method was expanded by using the *phs1* locus for the knock-in of the second expression construct. Successful replacement of *pks1* and *phs1* by two expression constructs comprising hygR or natR cassettes, mediated by the two RNPs expressed from pAMA/tRNA-*pks*1^PS2^-*phs*1^PS1^, is expected to yield white *hygR* and *natR* transformants which can be immediately identified on the transformation plates and should contain both expression constructs (black-white screening) (Figure 5A).

As proof-of-principle, the interaction of the conserved White Collar GATA-type transcription factors (TFs) in *K. petricola* was studied. White Collar 1 is a blue light receptor that forms a complex (called WCC) with White Collar 2 in the nucleus to induce the transcription of light-responsive genes (Yu and Fischer, 2019). The *K. petricola* orthologs WCL1/2 (white collar-like1/2) contain the characteristic protein domains for sensing blue light (LOV—light-oxygen-voltage), for protein-protein interactions (PAS—Per-Arnt-Sim) and DNA binding (ZF—GATA-type zinc finger) (Supplementary Sequences 2, 3) and share 28/27% aa identity with *A. nidulans* LreA/B, 34/41% aa identity with *Neurospora crassa* WC-1/WC-2, 42/43% aa identity with *B. cinerea* WCL1/2, and 54/56% aa identity with the *Exophiala dermatitidis* orthologs. The coding regions of the *K. petricola* genes were amplified and fused to full-length *gfp, mch* and split *gfp* (*gfpN, gfpC*). As (the non-functional) P*act1* is contained in some basal plasmids (P*act1::gfpN*), the promoter was replaced by P*gpdA* in the same cloning step, yielding fusion constructs *wcl1-gfp, wcl1-mch, gfp-wcl2, wcl1*-*gfpC*, and *gfpN-wcl2* under control of P*gpdA* or P*oliC* (Supplementary Table 3). The expression constructs were amplified using plasmids as template and primers containing the sequences homologous to the 5′ and 3′-noncoding regions of *pks1* or *phs1* as 5′ overhangs. WT:A95 protoplasts were co-transformed with different combinations of expression constructs and pAMA/ribo-*pks*1^PS2^ (single integration of *wcl1/2* fused to full-length *gfp/mch via* selection with HYG) or pAMA/ribo-*pks*1^PS2^-*phs*1^PS1^ (double integration of *wcl1/2* constructs at *pks1* and *phs1 via* selection with HYG and NTC) as schematically depicted in Figure 5B and specified in Supplementary Table 4. As expected, pink *hygR* transformants were obtained for *wcl1-gfp* and *gfp-wcl2* fusion constructs targeted to the *pks1* locus and white *hygR/natR* transformants for pairs of *wcl1/2* fusion constructs targeted to *pks1* and *phs1* (data not shown). Cell suspensions of three transformants per approach were examined by fluorescence microscopy revealing identical results. Thus, the single expression of WCL1-GFP or GFP-WCL2, and the co-expression of WCL1-mCH and GFP-WCL2 (co-localization approach) as well as of WCL1-GFP^C^ and GFP^N^-WCL2 (BiFC approach) led to bright fluorescence in the nuclei indicating that the method works and that WCL1 and WCL2 do interact in the nuclei of *K. petricola*. For none of the included negative controls, i.e., strains co-expressing GFP^C^ + GFP^N^, WCL1-GFP^C^ + GFP^N^, or GFP^C^ + GFP^N^-WCL2, fluorescence was detectable (Figure 5B, data not shown). As a pleasant side effect, it was found that white strains are more convenient for microscopy as no mechanical separation of the cells is necessary (compared to black cells) and cells do not form clumps (compared to black and pink cells).

In conclusion, the performed experiments demonstrated the practical application of BiFC coupled to a color-based screening approach for protein-protein interaction studies in *K. petricola*. Furthermore, the co-localization and interaction of the two *K. petricola* White Collar orthologs in the nucleus were proven. A set of four cloning vectors—pNAH-OGCGc, pNAH-OGCGn (Schumacher, 2012), pNDN-GGNTn, pNDN-GGNTc (this study)—is now available that allows to fuse the proteins of interest *via* variable linker sequences to the N- and C-terminal halves of GFP to test eight distinct combinations of fusion proteins for bimolecular fluorescence complex formation in *K. petricola* and beyond.

### Identification of Two Neutral Genome Loci for Targeted Insertion of Expression Constructs

Ectopic integrations of expression constructs may disrupt other genes, or the integration in a silent genomic region (heterochromatin) may prevent the expression of the inserted constructs. By targeted integration of constructs in defined genomic regions (knock-in) such negative effects are bypassed, and strains with predictable genetic backgrounds are generated. The established color-based screening procedures are suitable for promoter, localization and BiFC studies for which the pigmentation phenotype can be ignored as far as genetic backgrounds are identical. However, the phenotypic characterization of complemented deletion mutants or over-expression strains requires neutral genetic backgrounds, i.e., that inserted genes are expressed from noncoding genomic regions leaving all genes intact.

**Figure 6:**
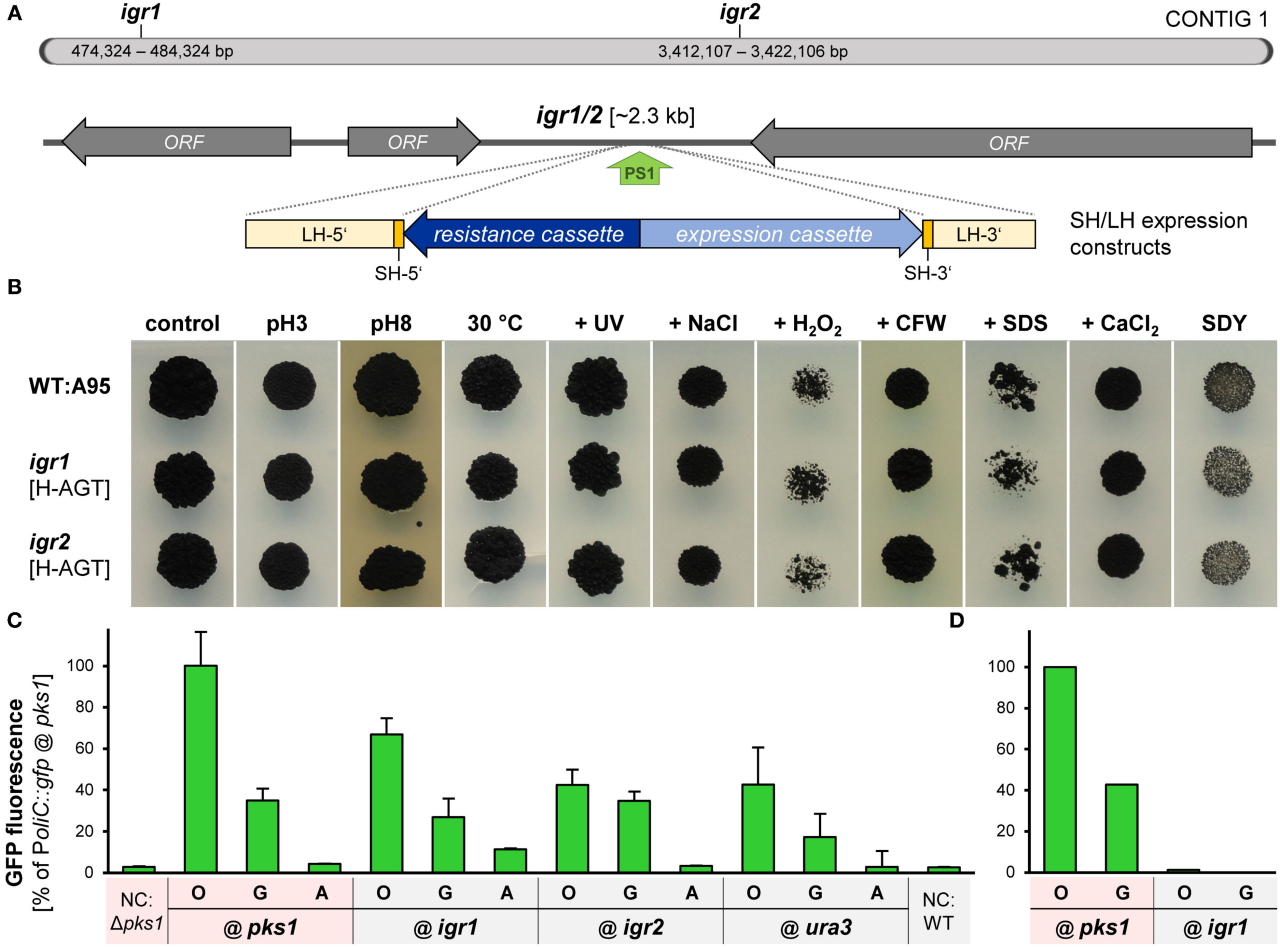
*Igr1* and *igr2* are neutral genomic regions for targeted integration of expression constructs into the genome of *K. petricola*. **(A)** Scheme of contig 1 with the studied intergenic regions 1 and 2 (*igr1/2*) of *K. petricola* A95. Two regions between converging ORFs with sizes of ~2.3 kb and moderate GC contents were chosen as candidates for neutral genomic regions. The green arrow (PS1) indicates the Cas9 cutting site. For insertion *via* HR (dashed lines), the expression constructs (blue arrows) were amplified by PCR using primers with SH sequences as 5′ overhangs (yellow boxes) or isolated by digestion from cloned plasmids (LH sequences; light yellow bars). **(B)** Insertions of different expression constructs at *igr1* and *igr2* do not result in growth phenotypes. Insertional strains were generated by co-transformation of WT:A95 protoplasts with pAMA/ribo-*igr*1^PS1^ or pAMA/ribo-*igr*2^PS1^ and different expression constructs flanked by appropriate homologous sequences to *igr1* or *igr2* as listed in Supplementary Table 4. The growth phenotypes under different stress conditions were evaluated for two insertional strains with different expression constructs (H-AGT, N-OCT) per genomic locus. Cell suspensions of the *gfp*- or *mch*-expressing strains were dropped onto solid SDYG pH 5 (control) and modified media. Results for WT, one strain per *igr* and dropping of 10^4^ cells are exemplarily shown. The entire dataset including details on the applied stresses is shown in Supplementary Figure 14. Pictures were taken after 9 days of incubation. **(C)** *Gfp* controlled by P*oliC* and P*gpdA* is considerably expressed when inserted in *igr1* or *igr2*. Strains comprising identical P*oliC*::*gfp* (O), P*gpdA::gfp* (G) and P*act1::gfp* (A) cassettes in different genomic regions i.e. *igr1, igr2* (B), Δ*pks1* (Figure 4A), and Δ*ura3* (Supplementary Figure 15) were cultivated for 4 days in liquid SDNG. Cell suspensions were dropped onto objective slides and submitted to the fluorescence microscopy. Images were captured with exposure times of 30 ms. Mean values and standard deviations of GFP intensities derived from 70 cells per strain. Negative controls (NC) were GFP-free wild-type (black) and Δ*pks1* (pink) cells. **(D)** GFP fluorescence in black strains cannot be detected by a microplate spectrophotometer. Results for pink and black strains expressing two P*oi*::*gfp* constructs (10^9^ cells per well) are exemplarily shown (technical duplicates only). For details on experimental set-up see Figure 4B.

Aiming the identification of such neutral insertion sites in the genome of *K. petricola*, two intergenic regions in transcriptionally active areas of contig 01 (data not shown), hereafter named *igr1* and *igr2*, were chosen as candidates for the experimental validation. *Igr1* and *igr2* represent shared terminator sequences of two adjacent genes, have sizes of ~2.3 kb, exhibit regular GC contents of 50%, lack restriction sites for frequently used enzymes and contain appropriate Cas9 sites (PS+PAM) in the middle of the sequences (Figure 6A; Supplementary Sequences 4, 5). The PSs were fused with the sgRNA scaffold for yielding pAMA/ribo-*igr*1^PS1^ and pAMA/ribo-*igr*2^PS1^. Cloning vectors were assembled comprising ~1.5-kb-long sequences that flank the considered Cas9 cutting sites/insertion sites, adapter sequences and multiple cloning sites for subsequent cloning, different resistance cassettes for selection (pIGRXR) and additonal expression cassettes containing *gfp* or *mcherry* (pIGRXH-AGT, pIGRXN-OCT) (Supplementary Table 3; Supplementary Figure 11). For the generation of first *igr* insertion strains, WT:A95 protoplasts were co-transformed with a CRISPR plasmid (pAMA/ribo-*igr*1^PS1^ or pAMA/ribo-*igr*2^PS1^) and the corresponding insertion construct isolated by digestion from pIGR1H-AGT, pIGR1N-OCT, pIGR2H-AGT, or pIGR2N-OCT (Supplementary Table 4). Four resistant transformants per construct were screened by diagnostic PCR for the correct insertion in *igr1* and *igr2*, respectively (Supplementary Figures 12, 13). Finally, the growth phenotypes of two strains per *igr* (from each approach) with verified insertion events and the wild-type strain A95 were assessed under 10 different stress conditions (Figure 6B). For this, cell suspensions of the strains were dropped onto solidified SDYG-based medium, that was adjusted to extreme pH values or supplemented with stress-inducing agents including hydrogen peroxide, calcofluor white and SDS. Heat and UV stress were applied to dropped cells on control medium (SDYG, pH 5) (see Supplementary Figure 14 for details). The tested *igr1/2* insertion strains showed wild-type-like growth phenotypes under all conditions, suggesting that the insertion of the ~4-kb-long sequences into *igr1* and *igr2* does not result in obvious phenotypes.

To analyze whether genes inserted into *igr1* or *igr2* are efficiently expressed, further *gfp* expression cassettes were inserted into *igr1* and *igr2* as well as into *ura3* (gene locus, considered as control; Supplementary Figure 15). For this, the expression cassettes were amplified by PCR using pNAH-GGG, pNDH-OGG, or pNDH-AGT as template and suitable primers attaching 75-bp-long homologous sequences to the expression constructs as described previously. These SH constructs were co-transformed with appropriate CRISPR plasmids (Supplementary Table 4), and six transformants per approach were screened by diagnostic PCR (Supplementary Figures 12, 13, data not shown). As a consequence, strains were generated that contain the same expression constructs (P*oliC::gfp*, P*gpdA::gfp*, or P*act1::gfp*) at different genomic loci, i.e., at *igr1* or *igr2 via* insertion in noncoding regions or at *ura3* or *pks1 via* replacement of coding sequences resulting in wild-type-like and melanin-free pigmentation. Finally, cell suspensions of the strains were examined by fluorescence microscopy. Representative results for one strain each are shown (Figure 6C). Moderate levels of GFP fluorescence were detected for P*oliC::gfp* and P*gpdA::gfp* constructs integrated at the four different loci, indicating that fluorescence microscopy works for melanin-containing (black) and melanin-free (pink) cells and that inserted constructs at *igr1* and *igr2* are well expressed (Figure 6D). As observed before (Figure 4), P*oliC* mediated best GFP fluorescence, followed by P*gpdA*. Fluorescence levels of P*act1::gfp* were comparable to those of the negative controls (*gfp*-lacking cells) confirming the unsuitablity of P*act1* for driving gene expression in *K. petricola*.

As a result, two intergenic regions in the genome of *K. petricola* were identified in which expression cassettes can be inserted by application of plasmid-based CRISPR/Cas9. Given the experimentally proven neutrality of insertions at *igr1* and *igr2*, phenotypes of insertional strains are due to the expression of the introduced gene(s). Therefore, constructs can be inserted in both *igr1* and *irg2* in a single step using pAMA/tRNA−*igr*1^PS1^−*igr*2^PS1^ (Supplementary Table 3) e.g., for co-overexpression of two genes in a wild-type-like background. While expression constructs from any plasmid can be amplifed using primers with short homologous sequences to *igr1* and *igr2*, the generated plasmids with long homologous sequences and five different resistance cassettes (pIGRXR) are particulary suitable for cloning of complementation constructs as depicted in Supplementary Figure 11. Constructs for transformation of *K. petricola* can be isolated by digestion due to attached multiple cloning sites (might be crucial for large constructs) or amplified by PCR using primers binding in the *igr*1/2^PS1^-flanking sequences. In fact, first deletion mutants were successfully complemented by targeting expression cassettes to *igr2* (R. Gerrits, pers. comm.).

## Discussion

Extremotolerant black fungi colonize natural and anthropogenic environments and are supposed to displace species that cannot resist the consequences of the climate change, e.g., desertification and temperature shifts. Understanding biodiversity and behavior of indicator species in terrestrial arid habitats is essential, while survival functions and environmental preferences of these organisms can be explored best with genetic approaches. A better understanding of these indicator species is crucial to protect materials and preserve cultural assets by using biocides as efficiently as possible and finding more environmentally friendly alternatives. More genomic data of black fungi from extreme habitats become available (Teixeira et al., 2017; Selbmann et al., 2020), enabling the identification of unique genes and assigning functions to newly identified genes as far as the fungus can be cultivated and techniques for its genetic manipulation are available.

We use the rock inhabitant *Knufia petricola* (strain A95) as a representative model to study organism-material and organism-organism interactions in subaerial biofilms (Noack-Schönmann et al., 2014b; Gerrits et al., 2021), and aim to develop advanced tools for reverse and forward genetic approaches for elucidating the survival functions and environmental preferences of black fungi (at least for Chaetothyriales). *K. petricola* exhibits moderate growth rates in axenic culture, is haploid and uninucleate which facilitates genetic studies as mutant phenotypes directly become visible. Despite the melanized cell walls, protoplasts are readily obtained by treatment with glucanase-rich lysing enzymes mixes and are transformed using Ca^2+^ and PEG immediately after their generation or storage for a prolonged period at −80°C. In a previous study, expression constructs of genes encoding fluorescent proteins were ectopically integrated into the genome of *K. petricola* and first genes were deleted by conventional gene replacement approaches using long homologous sequences. Applying the CRISPR/Cas9 system led to significantly increased rates of homologous recombination. Moreover, both *in vivo*-assembled RNPs (expression of *cas9* and target-specific sgRNA from AMA-bearing plasmid) and *in vitro*-assembled RNPs (purified Cas9 and synthesized target-specific sgRNA) were equally efficient when the donor DNA contained a resistance cassette for selection. Besides, the length of homologous sequences could be reduced to 65–75 bp (SH) which enables the generation of donor DNA with homologous sequences by a single PCR (Nai et al., 2013; Noack-Schönmann et al., 2014a; Voigt et al., 2020).

In this study, we proceeded with our efforts to design and optimize techniques for transformation and targeted genome editing of *K. petricola* by plasmid-based CRISPR/Cas9. The expression of multiple sgRNAs from a single plasmid simplifies the single-step introduction of multiple knock-out or knock-in mutations. The availability of three additional resistance selection markers enables the selection for simultaneous deletions of more than two genes as well as for insertions of multiple expression constructs. HygR, natR, genR and baR systems were found equally efficient for selection. The application of the suR system has some disadvantages as the half-life of the selective agent CME is limited and a second copy of an endogenous gene is expressed. The overexpression of the acetolactate synthase required for the synthesis of the branched-chain amino acids may interfere with the desired mutation and therefore, the usage of the suR cassette for knock-out approaches should be carefully considered. Resistance cassette-containing donor DNAs, optionally containing an expression cassette, are generated by PCR using plasmids from the pNXR-XXX and pR-XXX series as template and primers containing SH sequences as 5′ overhangs. Due to the modular structure of the cloning vectors, a set of standard primers can be used to target different expression constructs to gene loci essential for pigment synthesis (resistance- and color-based screening of transformants) or to neutral intergenic loci (resistance- and PCR-based screening of transformants). Alternatively, the 5′ overhangs of primers are homologous to the gene locus of interest.

*K. petricola* produces characteristic pigments, typical for many other Ascomycetes. DHN melanin is an inherent component of the cell walls of extremotolerant black fungi/yeasts while it is produced by specific cell types or under certain environmental conditions in filamentous fungi. Examples are the conidia of *A. fumigatus*, ascospores and perithecia of *Sordaria macrospora*, and the conidiophores, conidia, sclerotia and stressed mycelia of *B. cinerea* (Tsai et al., 1999; Engh et al., 2007; Schumacher, 2016). The synthesis pathway can be divided into three phases: (i) the *de novo* synthesis of the pentaketide T4HN (1,3,6,8-tetrahydroxynaphthalene), (ii) its modification to DHN, and (iii) the polymerization of DHN resulting in the highly complex DHN melanin (Butler and Day, 1998). The genes involved in the first two phases are highly conserved in all fungi and are tightly, partially, or not clustered in the genome. In contrast, different multicopper oxidases (laccases) may carry out the cross-linking of DHN in distantly related Ascomycetes (Jia et al., 2021). So far, the involvement of two *K. petricola* genes in melanogenesis have been demonstrated by genetic approaches. The mutation of *pks1* (polyketide synthase catalyzing the first step of phase I) results in an early block preventing the formation of any intermediates, while the mutation of *sdh1* (scytalone dehydratase catalyzing the second and fourth step of phase II) results in the accumulation of different water-soluble pathway intermediates (Voigt et al., 2020). The mutation of *pks1* yielding melanin-free mutants proved to be ideally suited to assay the effectiveness of different genetic engineering methods, the transformation success and efficiency (standard positive controls) as well as the targeted integration of expression constructs (color-based screening).

Strains with reduced melanization were obtained in this study by replacing the 1-kb-long region upstream of *pks1* which can be considered as the promoter sequence. However, the replacement by the hygR cassette (without another promoter sequence) did not abolish *pks1* expression suggesting that the regulatory region is longer and that the entire intergenic region upstream of *pks1* (4.323 kb) may contribute to its regulation. Strains in which *pks1* is expressed from P*gal1* or just contain the hygR cassette can be used for phenotyping as they are genetically stable in contrast to the *pks1* silencing mutants which were generated previously by triggering RNA interference (RNAi) (Voigt et al., 2020). The promoter studies performed provided important information on the activities of exogenous standard promoters for constitutive expression but failed to identify promoters for controllable gene expression in *K. petricola*. No indications were found that gene expression mediated by the two tested candidates (P*niaD*, P*gal1*) is altered by media containing inducing (nitrate, galactose) or repressing (amino acids, glucose) substances. This observation might be in accordance with the oligotrophic behavior of black fungi and highlights once more the importance to study the metabolic regulatory networks in these extremotolerant fungi. Apparently, knowledge gained from model fungi such as *S. cerevisiae, A. nidulans*, and *N. crassa* that are adapted to nutritionally rich environments, cannot be directly transferred to the group of black fungi that face nutrient scarcity in their natural habitats. An alternative system for controllable gene expression that does not dependent on endogenous regulatory networks, is the genetically engineered Tet-On system that comes with an inducible promoter and the corresponding regulator of bacterial origin. The regulator is activated upon binding of tetracycline or doxycycline and mediates gene expression through the promoter in a dose-dependent manner. The Tet-On system is successfully used in several fungi including different *Aspergillus* spp. and *F. fujikuroi* (Meyer et al., 2011; Helmschrott et al., 2013; Janevska et al., 2017) and thus may be implemented in *K. petricola* as well.

Carotenoids are unmasked by elimination of melanin synthesis in black fungi; however, the amounts and compositions of carotenoids may differ between species. Melanin-deficient mutants of *K. petricola* have a pronounced reddish pigmentation while those of the closely related black yeast *E. dermatitidis* exhibit a fainter coloration (Chen et al., 2014; Poyntner et al., 2018). Nine different carotenoids, i.e., phytoene, β-carotene, γ-carotene, ζ-carotene, lycopene, dihydrolycopene, didehydrolycopene, torulene, and torularhodin have been extracted and identified from *K. petricola* cells (Gorbushina et al., 2008; Flieger et al., 2018). The two essential genes for carotenogenesis—both enzymes catalyze several steps—are often clustered with genes encoding a carotenoid oxygenase for retinal formation and a microbial opsin that incorporates retinal for absorbing green light (Thewes et al., 2005; Chen et al., 2014; Schumacher et al., 2014). This cluster is conserved in *K. petricola*, and mutants of *phs1* and *phd1* do not produce colored carotenoids. However, *phd1* mutants may accumulate colorless phytoene (Voigt et al., 2020). By chance, a third carotenoid mutant was obtained in this study by random editing of *phs1*. The new mutants contain slightly different in-frame deletions in the N-terminal lycopene cyclase domain and exhibit a remarkable orange coloration, which indicates an altered carotenoid composition. Mutations in this protein domain were also reported to change the carotenoid composition in *N. crassa*; here, they caused the shift from orange to reddish carotenoids (Diaz-Sanchez et al., 2011).

Simultaneous mutation of *pks1* and *phs1* yielding white colonies allowed to detect the targeted integration of two expression constructs for co-localization and BiFC studies (black-white screening) and proved to be beneficial for fluorescence microcopy. Melanized cells form clumps and must be mechanically separated when single cells are needed. Even melanin-deficient cells have the tendency to accumulate and to form clumps after mechanical separation, possibly due to hydrophobic interactions in polar solvents. In contrast, white cells lacking both DHN melanin and carotenoids are found as single cells in suspension cultures and thus, no mechanical separation prior to microscopy is needed which decreases the risk of observing artifacts in damaged cells. Yet it is unknown whether the decreased cohesiveness of melanin-free cells depends on the absence of melanin *per se* or on the altered quantity and composition of EPS. Melanin-free mutants produce more EPS containing fewer pullulan-related glycosidic linkages. The latter enable melanin-producing strains to attach more strongly to olivine and dissolve it at a higher rate (Breitenbach et al., submitted). In sum, the concurrent replacement of the two essential pigment genes by expression constructs is advantageous for fluorescence microscopy approaches, at least for studying gene expression, and the localization and interactions of intracellular proteins. As the deletion of *ade2* (essential enzyme for adenine synthesis) in the *pks1-*/*phs1-* background resulted in rose-colored colonies due to the accumulation of pathways intermediates (data not shown), the *ade2* locus appears suitable for the simultaneous integration of a third expression construct (black-rose screening).

Sunlight is a source of stress for exposed organisms and can be an environmental cue for decision-making when it is sensed by the organism. Light-absorbing proteins (photoreceptors) in fungi are located in membranes (green-light absorbing opsins), the cytosol or the nucleus (near-UV, blue, and red/far-red sensors) and thus, light must pass the melanized cell wall to be sensed and to induce a response. Similarly, fluorescence microscopy relies on the excitation of intracellularly localized fluorescent proteins or incorporated fluorescent dyes by specific wavelengths. The fact that fluorescent microscopy is feasible with fully melanized *K. petricola* cells was already demonstrated. Here, we studied the impact of melanization by quantifying GFP fluorescence intensities in melanin-containing and melanin-free strains expressing different P*oi::gfp* constructs. In conclusion, melanization only marginally affected fluorescence intensities in single cells. By contrast, fluorescence intensities of high-density cell suspensions that mimic biofilm growth could be measured for melanin-free cells but not for melanin-containing cells. This suggests that only the outer exposed cells of a black biofilm can sense the actual light condition and that cells of melanin-free biofilms may differently respond to light. High numbers of photoreceptor-encoding genes in the genomes of *K. petricola* and other exposed black fungi support the relevance of light and its sensing for regulation of metabolism and stress responses. With 12 photoreceptors, *K. petricola* has even more photoreceptors than the plant pathogen *B. cinerea* (eleven known photoreceptors) that coordinates growth, virulence, and differentiation by sensing wavelengths of the entire visible spectrum and beyond (Schumacher and Gorbushina, 2020). Higher Ascomycetes may sense and respond to an array of wavelengths or certain wavelengths only. Blue light responses mediated by the transcriptional White Collar Complex—firstly identified and named after the mutant phenotype in *N. crassa* (Liu et al., 2003)—may have evolved early and retained in almost all fungi. Here, we demonstrated the co-localization and interaction of the two *K. petricola* orthologs in the nuclei as a first effort to elucidate the light signaling network in *K. petricola*.

## Conclusions

Black fungi dominate the harshest terrestrial ecosystems thus maintaining the balance and functionality of these stress-tolerant microbial communities (Selbmann et al., 2020) as well as belong to an ancestral lineage of the Chaetothyriales (Gueidan et al., 2008). We continue developing the rock-inhabiting fungus *K. petricola* as a model to obtain molecular insights into how black fungi interact with environmental challenges, materials and phototrophic partners. With the available advanced genetic tools, shared phenotypic traits of extremotolerant black fungi can be dissected. Thus, genetic foundations and regulation of pigment synthesis, the unconventional cell division cycles (Mitchison-Field et al., 2019), morphological and physiological adaptations to extreme environments (Tesei et al., 2021), the formation of (multispecies) biofilms (Gorbushina and Broughton, 2009), and mineral weathering mechanisms (Gerrits et al., 2021) can be elucidated. In addition, the established protocols and knowledge gained from *K. petricola* form a starting point for making other extremotolerant black fungi accessible to genetic manipulation. Protocols for generation and transformation of protoplasts can be adapted, pAMA-based plasmids can be used for delivery of sequences encoding Cas9 and target-specific sgRNA for *in-vivo* RNP formation (at least in the Chaetothyriales), essential genes for pigment synthesis can be targeted for monitoring transformation and CRISPR/Cas9 efficiencies, and the described modular cloning vectors can be used for generation of knockout mutants, strains with altered gene expression levels or strains expressing fusion proteins for localization and interaction studies. Genetic perspectives arising from these approaches will form a foundation for biodeterioration, geomicrobiological and astrobiological experiments as well as gain significance in functional studies of symbiotic capacities and polyextremotolerance of black fungi.

## Data Availability Statement

The original contributions presented in the study are included in the article/Supplementary Material, further inquiries can be directed to the corresponding author.

## Author Contributions

EE contributed to the design of the study, performed experimental work and data analysis, wrote the draft manuscript, reviewed, and edited the manuscript. SN performed experimental work and data analysis, reviewed, and edited the manuscript. AG contributed to reviewing and editing of the manuscript. JS designed and coordinated the study, analyzed data, reviewed, and edited the manuscript. All authors contributed to the article and approved the submitted version.

## Funding

This work was supported by internal funds of the BAM.

## Conflict of Interest

The authors declare that the research was conducted in the absence of any commercial or financial relationships that could be construed as a potential conflict of interest.

## Publisher's Note

All claims expressed in this article are solely those of the authors and do not necessarily represent those of their affiliated organizations, or those of the publisher, the editors and the reviewers. Any product that may be evaluated in this article, or claim that may be made by its manufacturer, is not guaranteed or endorsed by the publisher.
