# Supplementary material for: Genetic Engineering of the Rock Inhabitant Knufia petricola Provides Insight Into the Biology of Extremotolerant Black Fungi
Source: Front Fungal Biol. 2022 Apr 8;3:862429. doi: 10.3389/ffunb.2022.862429 (PMC10512386; doi:10.3389/ffunb.2022.862429)
Supplement: Supplementary file 1 [file Data_Sheet_1.PDF]

Supplementary Material

Supplementary Figure 1. *In vivo* assembly of CRISPR/Cas9 complexes...

Supplementary Figure 2. Strategies for cloning of pAMA-based CRISPR plasmids.

Supplementary Figure 3. Plasmid-based CRISPR/Cas9 with tRNA-sgRNA cassettes.

Supplementary Figure 4. Multiplexed gene editing through CRISPR/Cas9 and DNA oligos.

Supplementary Figure 5. pNXR-XXX cloning vectors have the same modular structure...

Supplementary Figure 6. pR-XXX cloning vectors consist of resistance and expression cassettes…

Supplementary Figure 7. Generation of pink replacement mutants using genR, baR and suR...

Supplementary Figure 8. Generation of black *gfp* expressing strains using genR, baR and suR...

Supplementary Figure 9. Strategies for targeting *gfp* expression constructs to the *pks1* locus.

Supplementary Figure 10. Replacement of P*pks1* by promoters of interest in the *phs1*- background.

Supplementary Figure 11. pIGRXR cloning vectors for targeted insertion of constructs into *igr1/2*.

Supplementary Figure 12. Insertion of expression constructs in the intergenic region 1.

Supplementary Figure 13. Insertion of expression constructs in the intergenic region 2.

Supplementary Figure 14. Constructs in *igr1* and *irg2* do not result in obvious phenotypes.

Supplementary Figure 15. Integration of *gfp* expression constructs by replacement of *ura3*.

Supplementary Table 1. *K. petricola* strains used in this study.

Supplementary Table 2. Oligonucleotides used in this study.

Supplementary Table 3. Plasmids cloned in this study.

Supplementary Table 4. Transformations of *K. petricola* protoplasts carried out in this study.

Supplementary Sequence 1. *K. petricola* galactokinase 1.

Supplementary Sequence 2. *K. petricola* white collar-like 1.

Supplementary Sequence 3. *K. petricola* white collar-like 2.

Supplementary Sequence 4. *K. petricola* intergenic region 1 (*igr1*).

Supplementary Sequence 5. *K. petricola* intergenic region 2 (*igr2*).

Supplementary References

1 Supplementary Figures

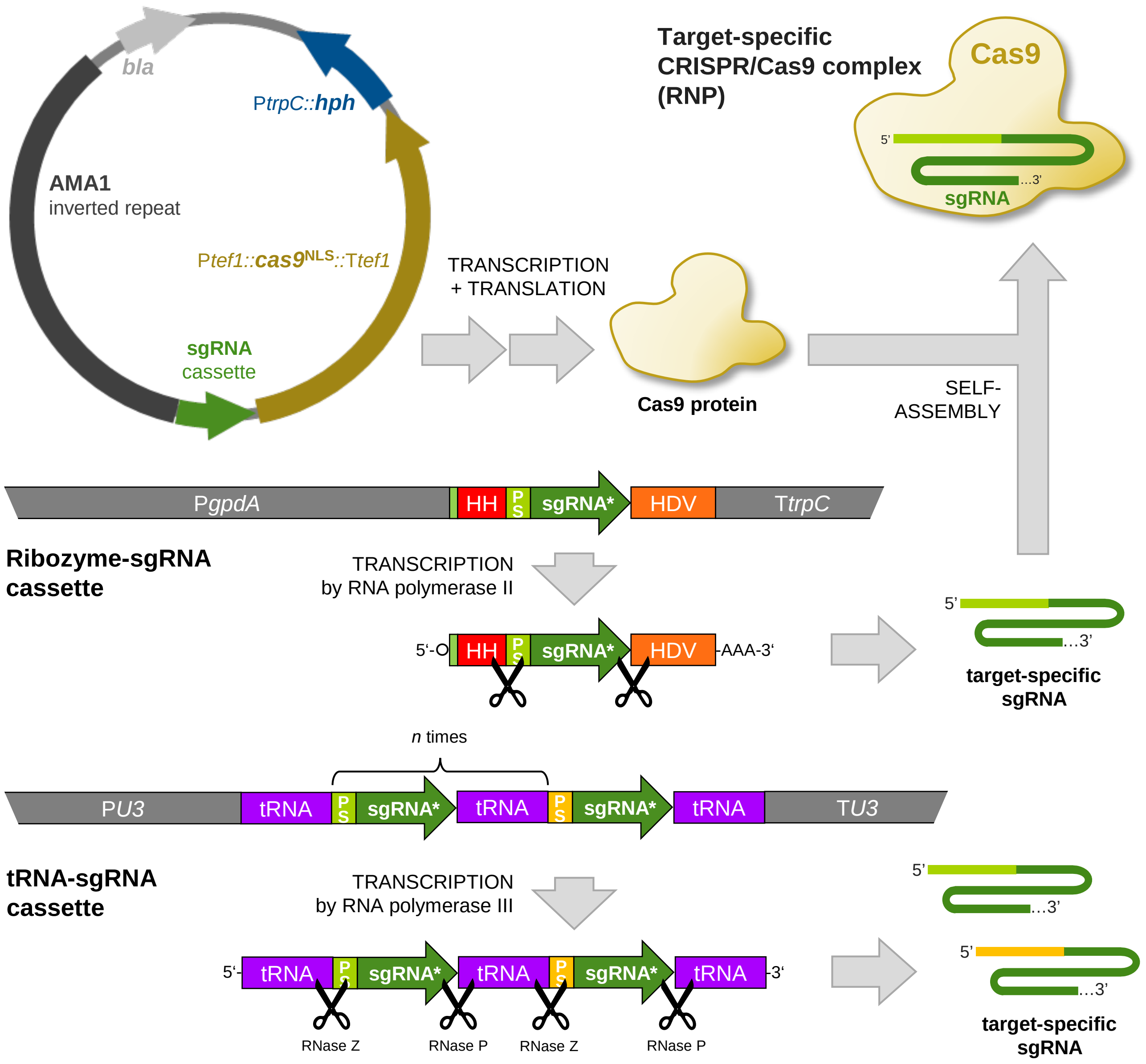

Supplementary Figure 1. *In vivo* assembly of CRISPR/Cas9 complexes (ribonucleoproteins, RNPs) from plasmid-encoded components. In this transient expression system, cassettes for expression of *cas9* and target-specific sgRNA [protospacer (PS) + sgRNA backbone (*)] are combined with a hygR cassette (P*trpC*::*hph*) and the AMA1 replicator sequence from *Aspergillus nidulans* in a circular plasmid. *Cas9* is codon-optimized for *Aspergillus niger*, fused to an encoded SV40 nuclear localization signal [PKKKRKV] and under control of the regulatory sequences of *A. nidulans tef1*. In ribozyme-sgRNA cassettes the sgRNA is flanked by sequences encoding ribozymes [hammerhead (HH) and hepatitis delta virus (HDV)] liberating the sgRNA from the larger mRNA transcript in the nucleus. Transcription is controlled by regulatory sequences from *A. nidulans* (Nødvig et al., 2015). In tRNA-sgRNA cassettes the sgRNA (one or more) is flanked by tRNA sequences and released from the transcript by the endogenous tRNA processing machinery. Transcription by RNA polymerase III is mediated by U3 regulatory sequences from *Aspergillus fumigatus* (Nødvig et al., 2018).

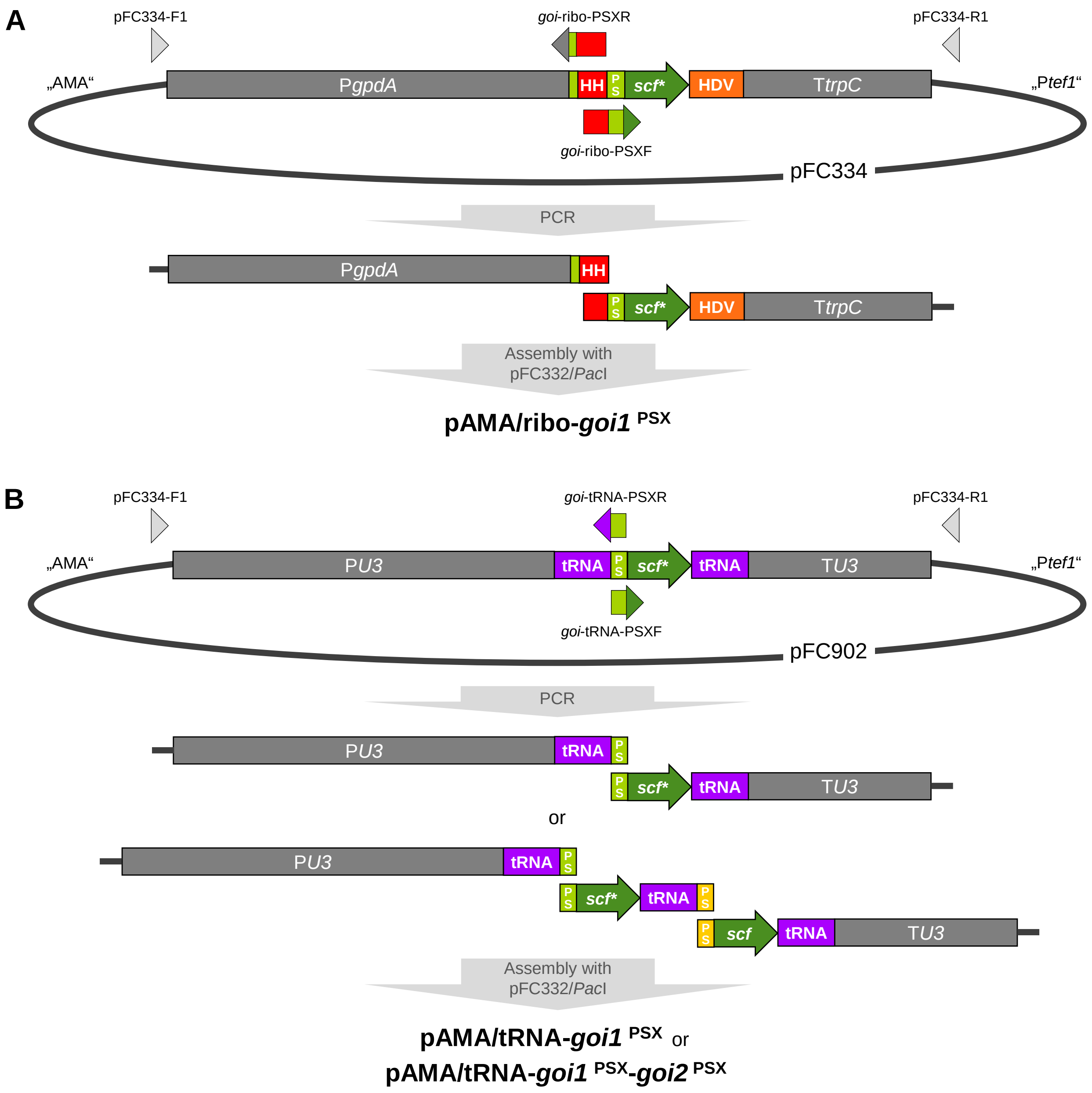

Supplementary Figure 2. Strategies for cloning of pAMA-based CRISPR plasmids. (**A**) Two target-specific sgRNA fragments additionally containing P*gpdA* (0.570 kb) or T*trpC* (0.463 kb) are generated by high-fidelity PCR using pFC334 as template (Nødvig et al., 2015), standard primers binding in pFC334/332 (pFC334-F1/-R1) and designed target-specific primers (*goi*-ribo-PSXF/-PSXR) comprising the protospacer (PS) and the 6-bp-long cleavage site for the HH ribozyme (light green boxes). Assembly of the sgRNA fragments is mediated via the 30-bp-long overlap in the HH sequence, the assembly with pFC332 via 25-bp-long overlaps generated by primers pFC334-F/-R1 during the PCR. (**B**) The target-specific sgRNA fragments are generated by high-fidelity PCR using pFC902 as template (Nødvig et al., 2018), standard primers (pFC334-F1/-R1) and the target-specific primers binding to the sgRNA scaffold (*scf*) (*goi*-tRNA-PSxF) or the tRNA (*goi*-tRNA-PSxR) and comprising the PS sequences as 5’ overhangs. Assembly of two, three or more sgRNA fragments is mediated via 20-bp-long overlaps i.e. the attached PS sequences.

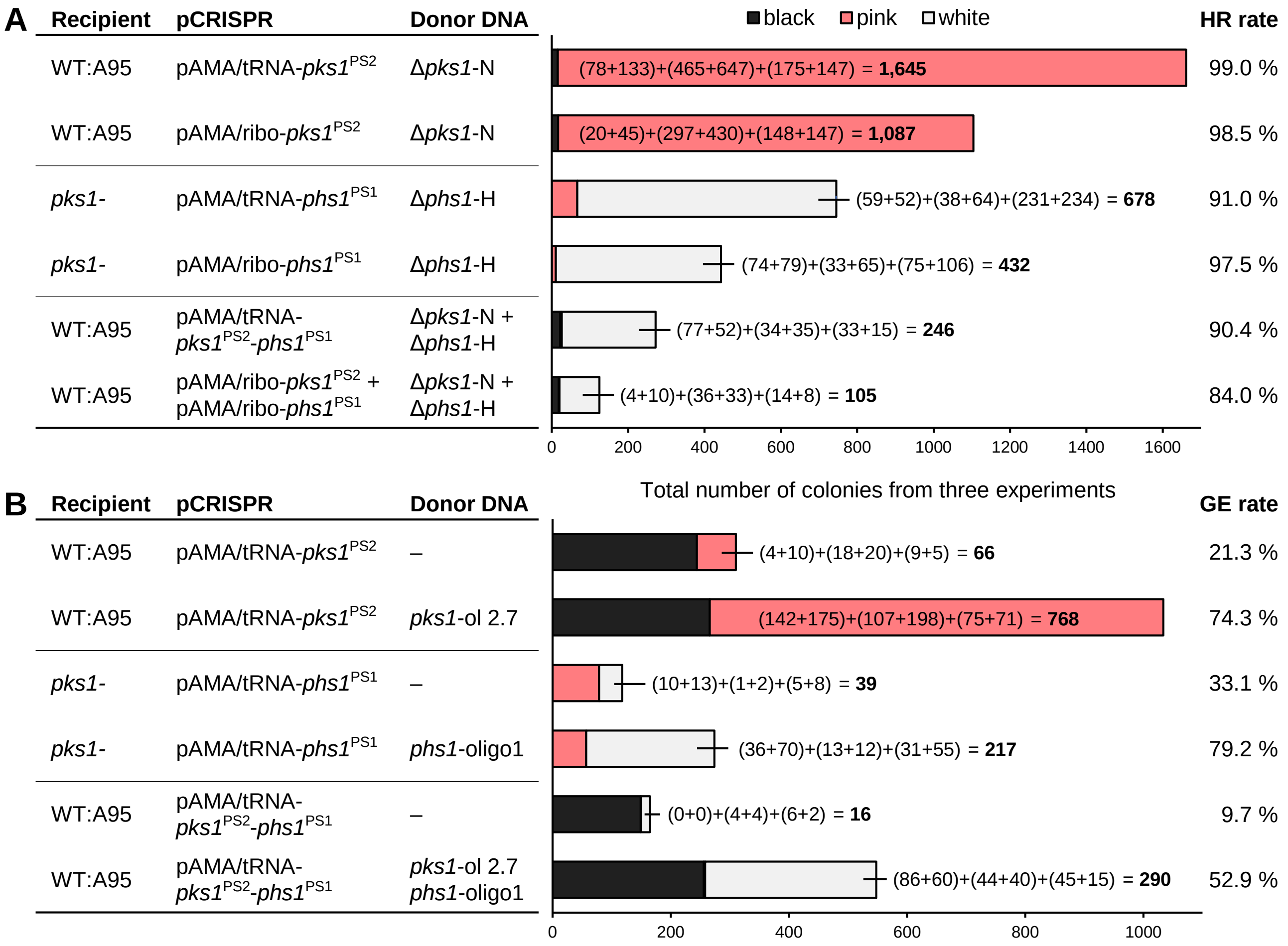

Supplementary Figure 3. Plasmid-based CRISPR/Cas9 with tRNA-sgRNA cassettes. Protoplasts of the listed recipient strains were co-transformed with the CRISPR plasmids and donor DNA as indicated (Supplementary Table 4). Donor DNA for replacement of *pks1* and *phs1* were resistance cassettes with 75-bp-long 5’ overhangs **(A)** and 80-bp-long single-stranded oligonucleotides for targeted gene editing **(B)**. Data i.e. the numbers of differentially pigmented colonies derive from three independent transformation experiments yielding two plates each (numbers of colonies in brackets). Pictures shown in Figure 2A, B derive from the first experiment. Rates of homologous recombination (HR) and gene editing (GE) were calculated by dividing the number of pink or white colonies through the total number of colonies counted.

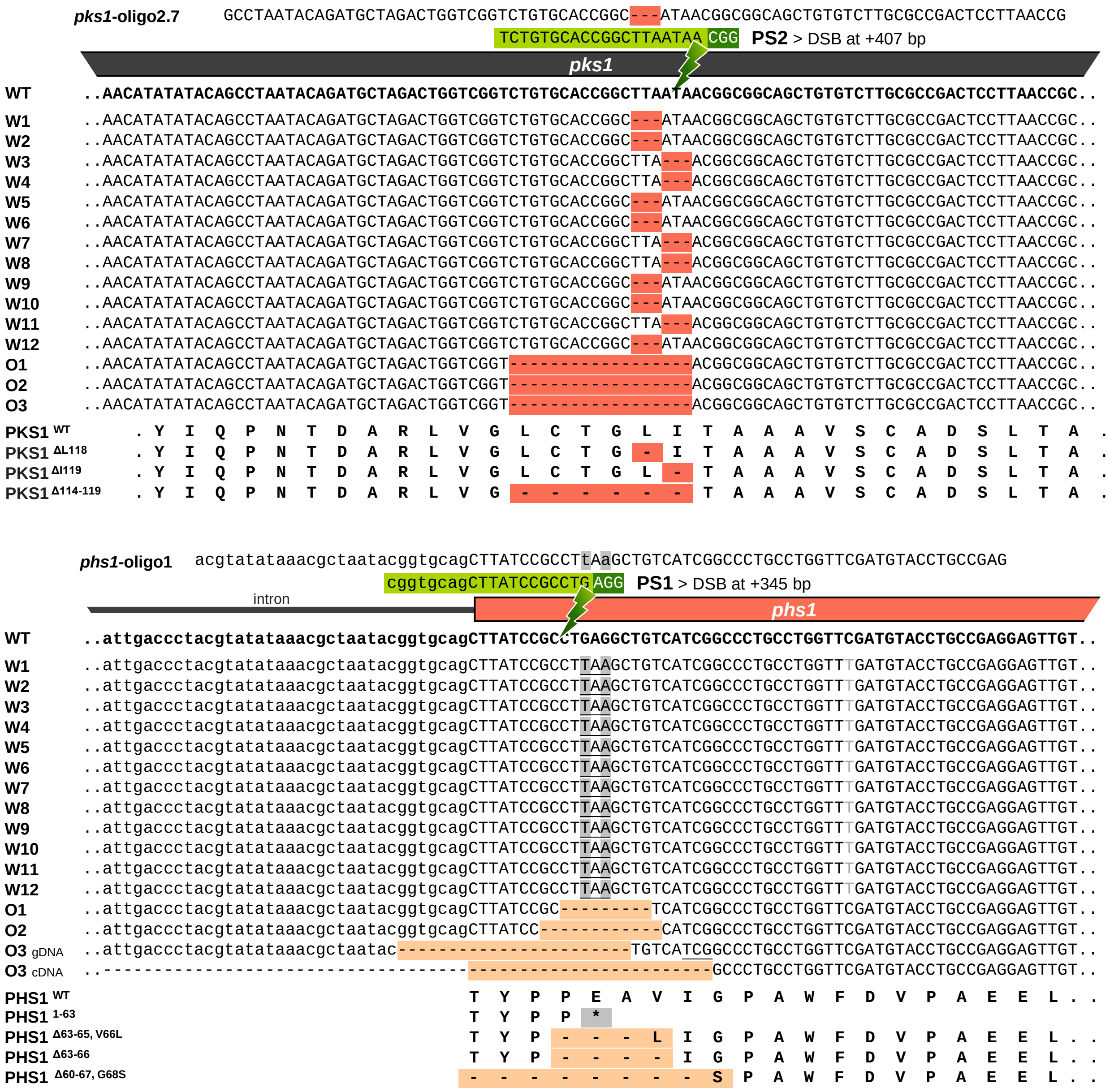

Supplementary Figure 4. Multiplexed gene editing through CRISPR/Cas9 and DNA oligonucleotides. White mutants (W1-W12) were obtained from co-transformation of WT:A95 protoplasts with circular pAMA/tRNA-*pks1*^{PS2}-*phs1*^{PS1} and the single-stranded DNA oligonucleotides *pks1*-oligo2.7 and *phs1*-oligo1 (Supplementary Table 4). Orange mutants (O1-O3) were obtained from the transformation of *pks1*- protoplasts with circular pAMA/tRNA-*phs1*^{PS1} (Figure 2B,C). The PS-spanning regions of pigment-deficient mutants were amplified by PCR (*pks1*^{PS2}: *pks1*-PS1-sF2/*pks1*-RNAi-R1; *phs1*^{PS1}: *phs1*-hi5F/*phs1*-WT-R) and sequenced. Two different mutations causing in-frame deletions of 3 bp in *pks1* combined with the same 2-bp-mutation in *phs1* resulting in a premature stop codon were identified in the twelve white mutants. O1-3 contain the same 18-bp-long in-frame deletion in *pks1* and different in-frame mutations of 9 bp (> 3-aa-deletion plus one aa exchange), 12 bp (> 4-aa-deletion) or 23 bp in *phs1*. As the deletion in the latter mutant includes the 3’ splicing site of an intron, cDNA of mutant O3 was synthesized and sequenced as well.

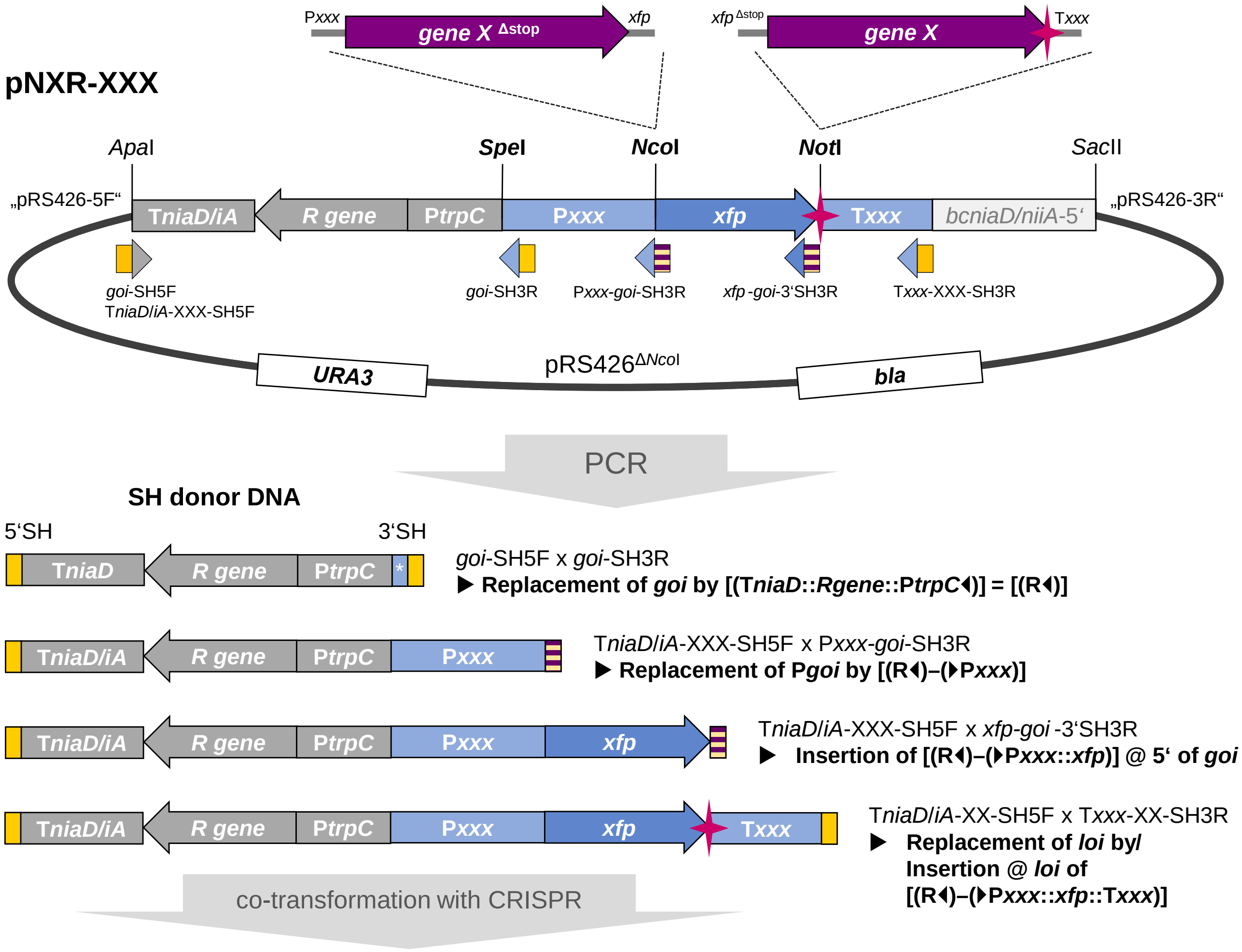

Supplementary Figure 5. pNXR-XXX cloning vectors have the same modular structure and were primarily designed for usage in *B. cinerea* (Schumacher, 2012). The plasmids contain a resistance (R) cassette and an expression (E) cassette in opposite orientation that are flanked by the 5' and 3' noncoding regions of *bcniaD* (nitrate reductase) or *bcniiA* (nitrite reductase) for facilitating targeted integration into respective gene loci of *B. cinerea*. The 3' noncoding regions represent the terminators of the two genes, and thus function as terminators (T*niaD*, T*niiA*) of the R genes (*hph*, *nat1*, *bar*, *nptII*, *sur*, *ble*, *fferg27* in these constructs. Genes can be inserted upstream or downstream of *xfp* (*mch*, *gfp*, *dsred*, *gfpN*, *gfpC*) or can replace *xfp* by using the *Nco*I and/or *Not*I sites. Modules are contained in different combinations in the available plasmids (P*xxx* – P*oliC*, P*act1*, P*gpdA*; T*xxx* – T*gluc*, T*tub1*, T*trpC*). New modules in recently cloned plasmids are P*gpdA* and the R genes *nptII*, *bar* and *sur* (Supplementary Table 3). R cassettes flanked by ~75-bp-long homologous (SH) sequences are generated by PCR using plasmids of the pNDR-OGG series as template and primers binding in conserved regions of the R cassettes (T*niaD*, short part of P*oliC* [*]) and containing 5' overhangs homologous to the 5'- and 3'-noncoding regions of the *goi*. Similarly, primers binding in the R (T*niaD*/*iA*-XXX-SH5F) and E cassettes (T*xxx*-XXX-SH3R, P*xxx*-*goi*-SH3R, *xfp*-*goi*-3'-SH3R) and containing ~75-bp-long homologous sequences to a locus of interest as 5' overhangs can be used to amplify expression constructs for targeted integration into the fungal genome (yellow boxes – noncoding regions of the locus/gene of interest; striped boxes – coding sequence of gene of interest).

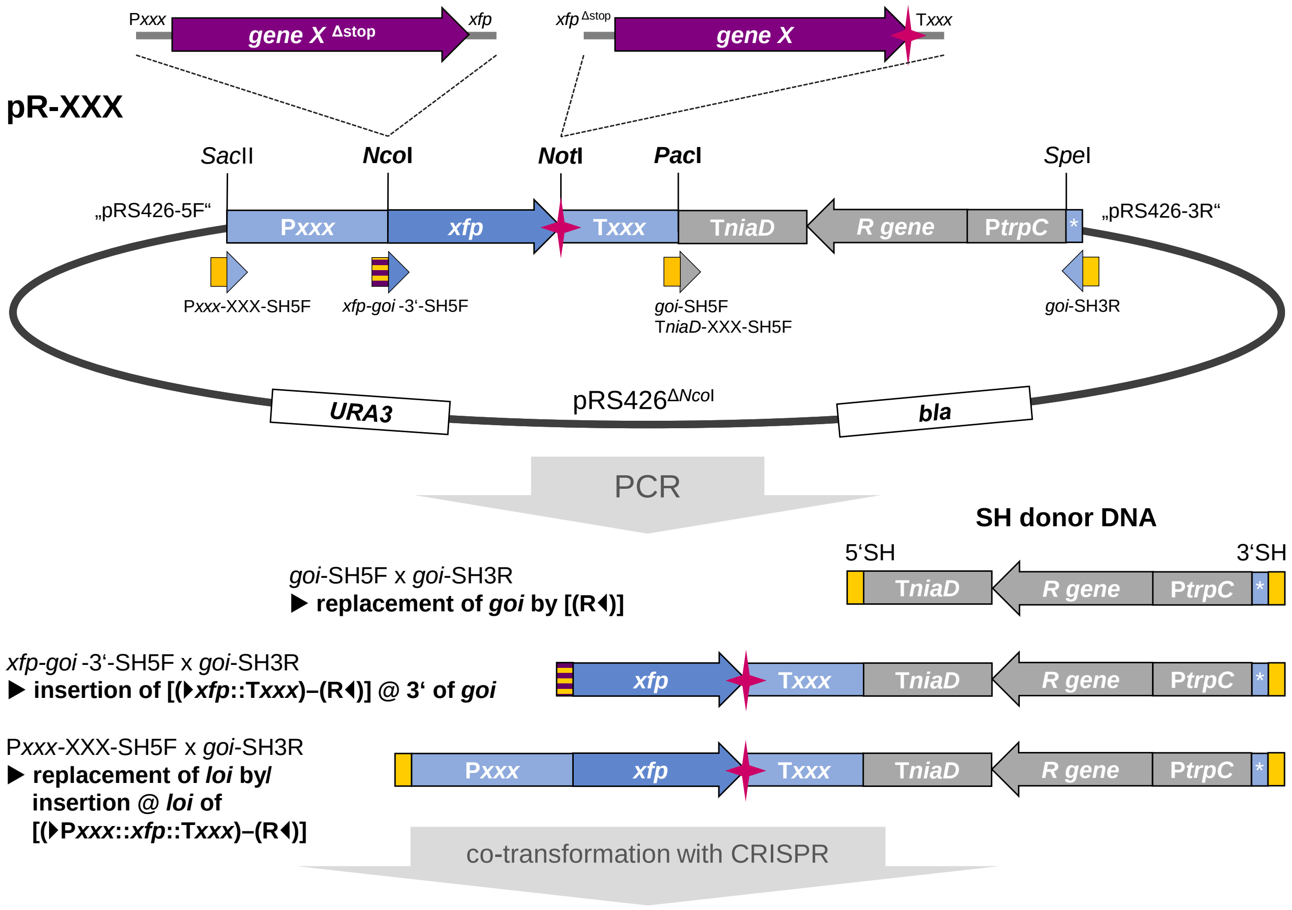

Supplementary Figure 6. pR-XXX cloning vectors consist of resistance (R) and expression cassettes (-XXX) from the pNXR-XXX series (Schumacher, 2012) that were assembled in the opposite orientation in pRS426$^{\Delta NcoI}$ (this study; Supplementary Table 3). The first set of vectors contain the expression cassette P*gpdA*::*gfp*::T*gluc* (-GGG) fused to one of the five available R cassettes for *K. petricola* (hygR [H], natR [N], genR [G], baR [P], suR [S]). pR-XXX can be used as template for amplification of SH fragments for expressing a *goi-xfp* fusion gene from the native (*goi*) promoter. pR-XXX contains a short part of P*oliC* (*) i.e. the *goi*-SH3R binding site that allows for the re-use of *goi*-SH3R from a standard gene replacement (KO) approach. Primer pairs for generating the target-specific donor DNA [(▶*xfp*::T*xxx*)–(T*niaD*::*R gene*::P*trpC*◀)]$^{goi\text{-}3'}$ from pR-XXX for knock-in downstream of the *goi* are *xfp-goi*-3'-SH5F/*goi*-SH3R. As the R cassettes contain T*niaD*, these plasmids can be used for the amplification of regular target-specific donor DNA for KO approaches as well i.e. [(T*niaD*::*R gene*::P*trpC*◀)]$^{\Delta goi}$ with primer pair *goi*-SH5F/*goi*-SH3R. Besides, pR-XXX vectors are suitable for cloning of gene fusion constructs by using the restriction sites *Nco*I (pH-, pN- and pP-XXX only) and *Not*I (all pR-XXX) as described for pNXR-XXX. SH/yellow boxes – noncoding regions of the locus/gene of interest (*loi*/*goi*); SH/striped boxes – coding sequence of *goi*; red asterisks – stop codons of *xfp*/*goi*.

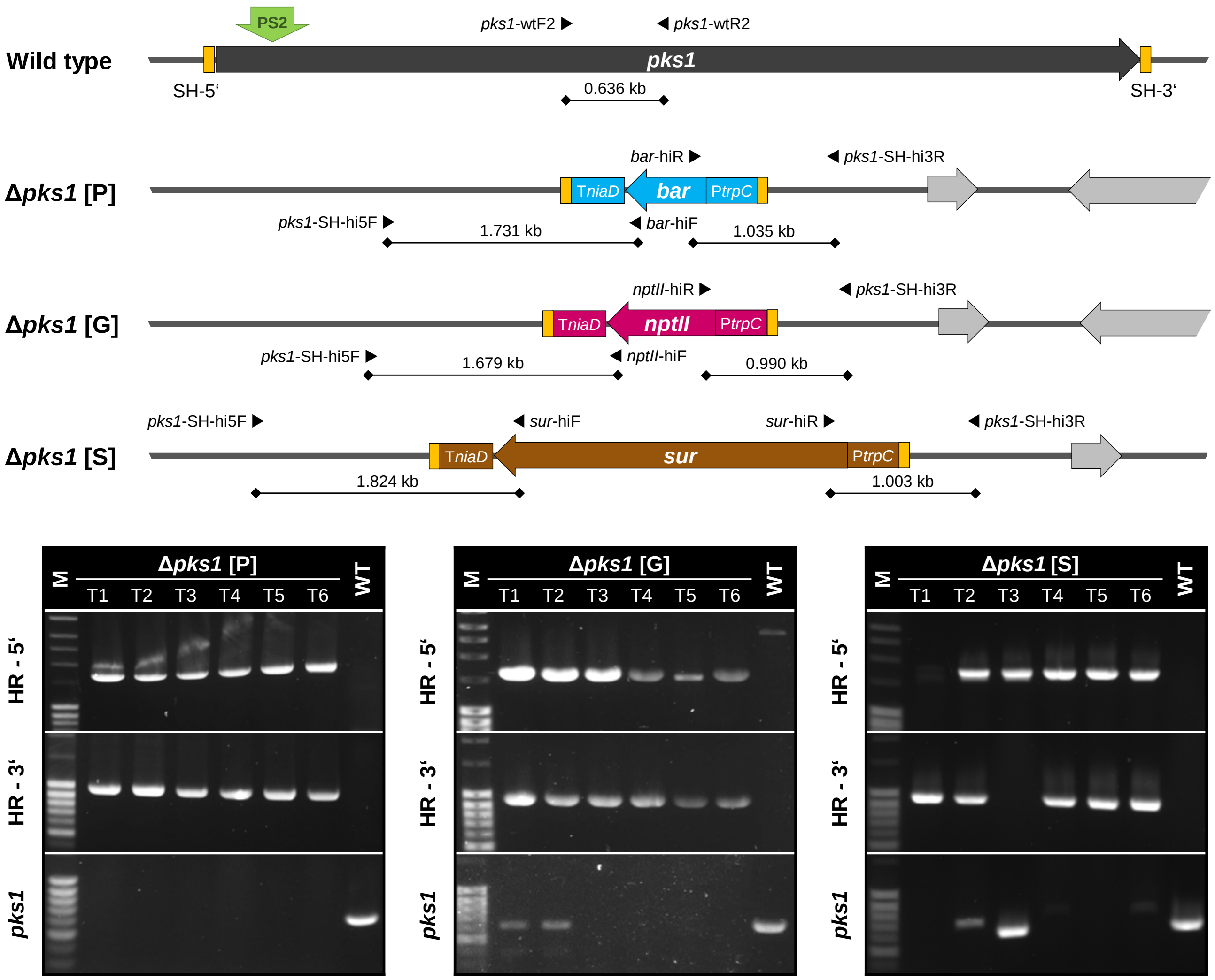

Supplementary Figure 7. Generation of pink replacement mutants using genR, baR and suR selection systems. Replacement fragments with 75-bp-long homologous sequences to the 5’ and 3’ noncoding regions of *pks1* and comprising genR [G], baR [P] or suR [S] cassettes were generated by PCR using the primer pair *pks1*-RT5F/*pks1*-RT3R and pNDP-OGG, pNDG-OGG or pNDS-OGG (Supplementary Table 3) as template. Protoplasts of WT:A95 were co-transformed with the amplicons and pAMA/ribo-*pks1*^{PS2} (Supplementary Table 4) yielding high numbers of pink colonies on the transformation plates (Figure 3). Diagnostic PCRs of six pink *genR*, *baR* and *suR* transformants with primer combinations shown in the scheme were performed. For most mutants the expected amplicons for HR events at 5’ and 3’ of *pks1*^{PS2} were detected.

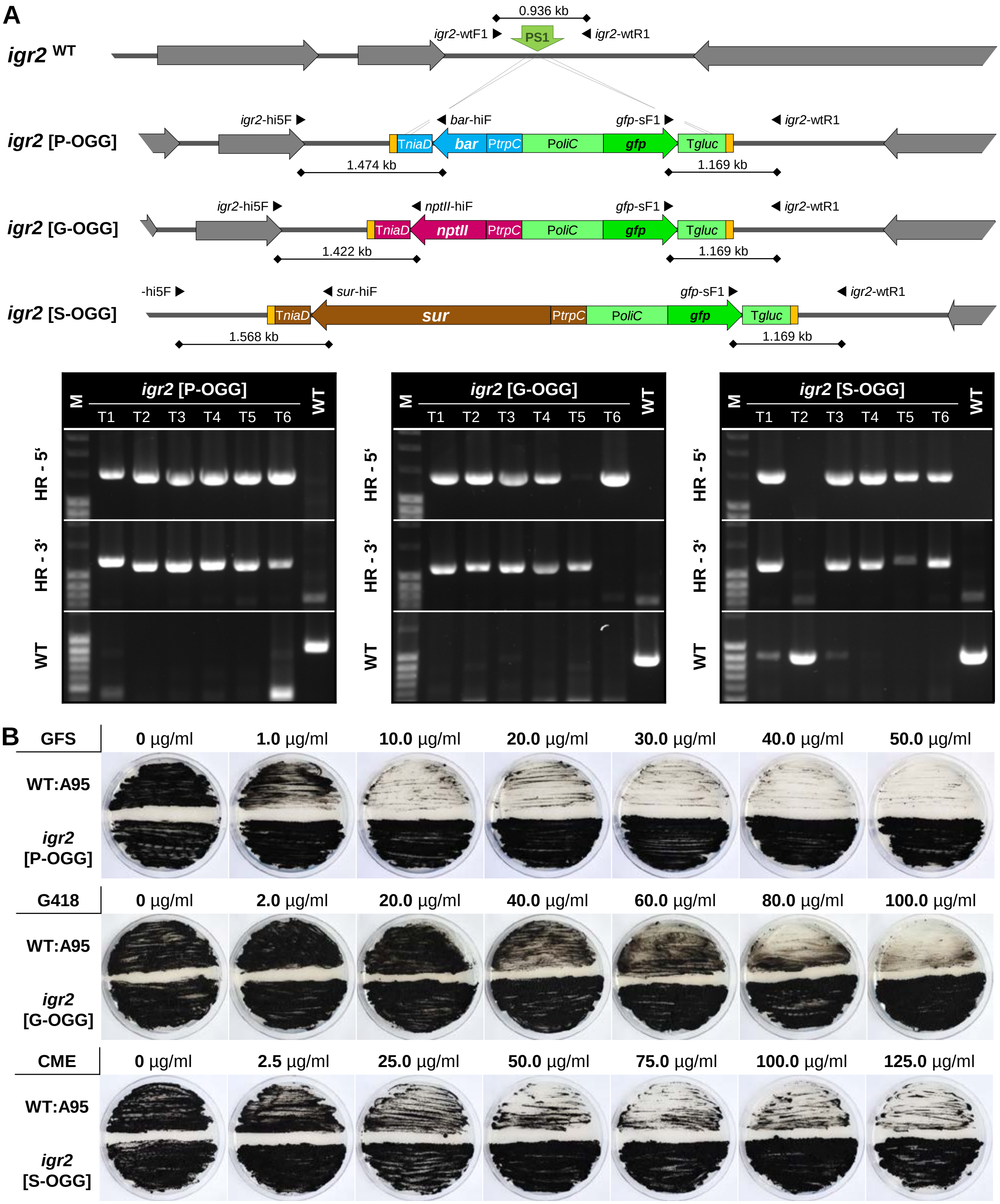
A
igr2 WT
0.936 kb
igr2-wtF1
PS1
igr2-wtR1
igr2 [P-OGG]
igr2-hi5F
bar-hiF
gfp-sF1
igr2-wtR1
TniaD
bar
PtrpC
PoliC
gfp
Tgluc
1.474 kb
1.169 kb
igr2 [G-OGG]
igr2-hi5F
nptII-hiF
gfp-sF1
igr2-wtR1
TniaD
nptII
PtrpC
PoliC
gfp
Tgluc
1.422 kb
1.169 kb
igr2 [S-OGG]
-hi5F
sur-hiF
gfp-sF1
igr2-wtR1
TniaD
sur
PtrpC
PoliC
gfp
Tgluc
1.568 kb
1.169 kb
M
igr2 [P-OGG]
T1 T2 T3 T4 T5 T6
WT
HR - 5'
HR - 3'
WT
igr2 [G-OGG]
igr2 [S-OGG]
B
GFS
0 µg/ml
1.0 µg/ml
10.0 µg/ml
20.0 µg/ml
30.0 µg/ml
40.0 µg/ml
50.0 µg/ml
WT:A95
igr2 [P-OGG]
G418
0 µg/ml
2.0 µg/ml
20.0 µg/ml
40.0 µg/ml
60.0 µg/ml
80.0 µg/ml
100.0 µg/ml
WT:A95
igr2 [G-OGG]
CME
0 µg/ml
2.5 µg/ml
25.0 µg/ml
50.0 µg/ml
75.0 µg/ml
100.0 µg/ml
125.0 µg/ml
WT:A95
igr2 [S-OGG]

Supplementary Figure 8. Generation of black *gfp* expressing strains using genR, baR and suR selection markers. **(A)** Strategies for targeted insertion of *gfp* expression cassettes into *igr2* using the new resistance cassettes. Fragments with 75-bp-long homologous sequences to *igr2* (Supplementary Figure 13; Figure 6) were generated by PCR using the primer pair T*niaD*-*igr2*-SH5F/T*gluc*-*igr2*-SH3R and pNDP-OGG, pNDG-OGG or pNDS-OGG (Supplementary Table 3) as template. Protoplasts of WT:A95 were co-transformed with the amplicons and pAMA/ribo-$igr2^{PS1}$ (Supplementary Table 4). Diagnostic PCRs of six arbitrarily chosen *genR*, *baR* and *suR* transformants with primer pairs shown in the scheme revealed high rates of homologous recombination (HR) at both sites of the Cas9 cutting site which is indicated as green arrow (PS1). **(B)** Wild type A95 is less sensitive to G418, GFS and CME when cells are spread with an inoculation loop onto the agar. In parallel with the growth assay shown in Figure 3, the same media i.e., solidified SDNG without (control) and with different concentrations of the selective agents, was inoculated with WT:A95 and the three resistant insertion strains *igr2* [G-OGG], *igr2* [P-OGG] and *igr2* [S-OGG] by streaking undefined numbers of cells taken from one-week-old surface-grown colonies using plastic inoculation loops. As this inoculation procedure resembles the transfer of cells of top-grown (putative resistant) colonies from transformation plates (primary selection) to fresh selective medium for confirming the transformation event (secondary selection), inhibitory concentrations of G418 (geneticin), GFS (glufosinate ammonium) and CME (chlorimuron ethyl) for efficient transformant selection were determined on this basis.

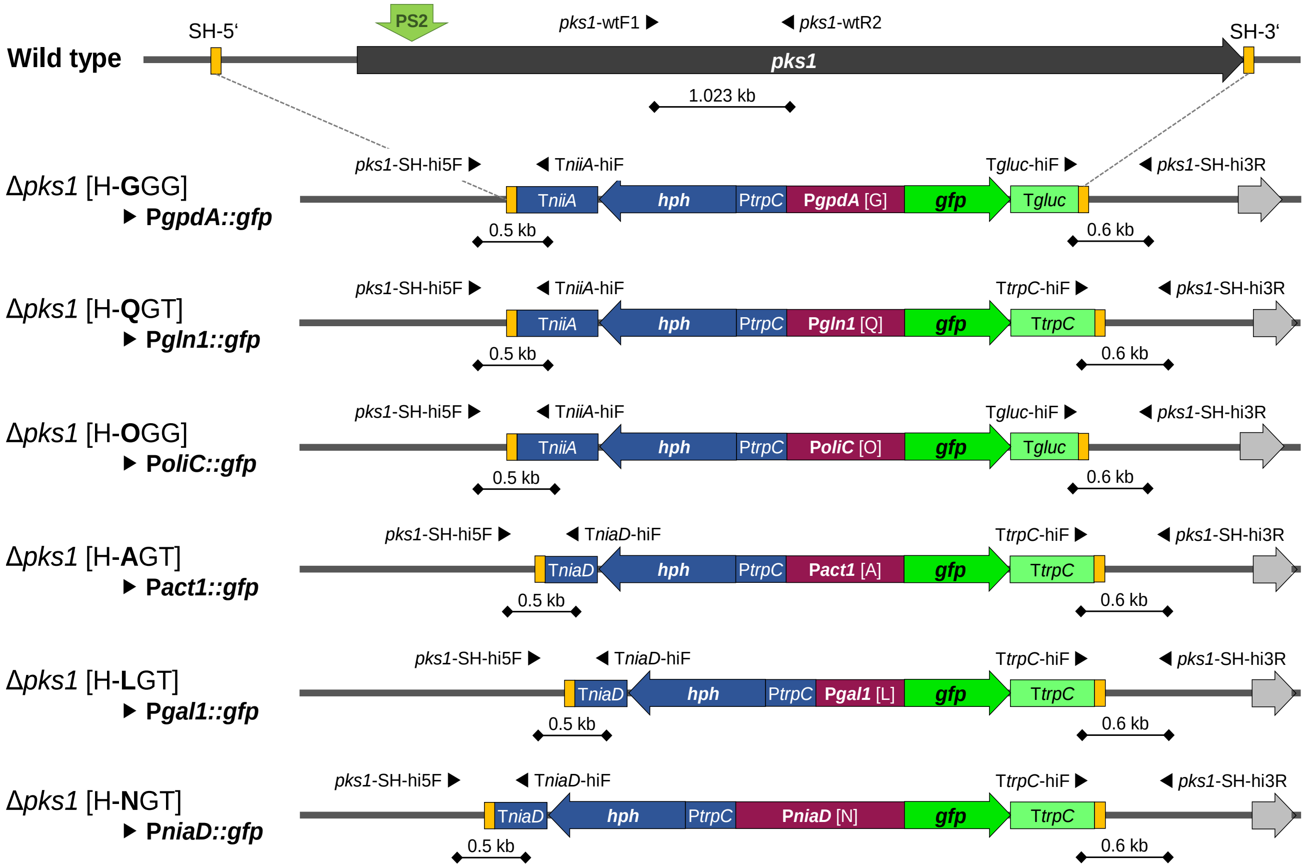

Supplementary Figure 9. Strategies for targeting *gfp* expression constructs to the *pks1* locus. For the comparative analysis of *gfp* expression from six different promoters (P*gpdA*, P*gln1*, P*oliC*, P*act1*, P*gal1*, P*niaD*; Table 3), the constructs (*gfp* expression cassette linked to a hygR cassette) were integrated into WT:A95 by replacing *pks1* including 1 kb of the upstream noncoding region. Expression constructs were amplified from plasmids (Schumacher, 2012) (Supplementary Table 3) using primers with 75-bp-long 5' overhangs homologous to the non-coding regions of *pks1* (yellow boxes SH-5' and SH-3') and co-transformed with the Cas9/sgRNA^{pks1}-delivering plasmid pAMA/ribo-*pks1*^{PS2} (Supplementary Table 4, Figure 4). Primers used for diagnostic PCRs (data not shown) and the site of the Cas9-induced DSB (PS2) are indicated.

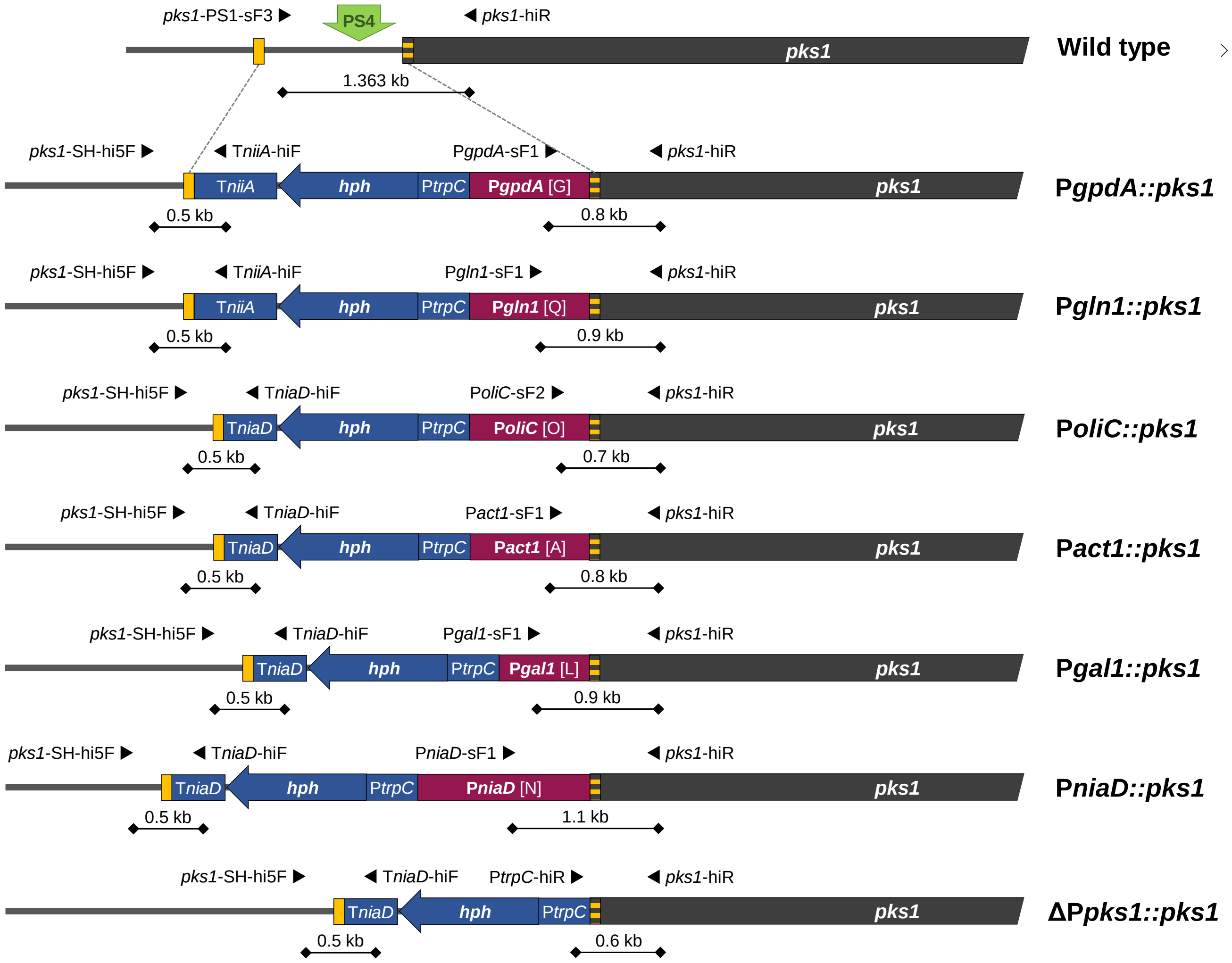

Supplementary Figure 10. Replacement of P*pks1* by promoters of interest (P*oi*) in the *phs1*- background. For comparative analysis of *pks1* expression (pigmentation) from different promoters (Table 3), the indicated constructs (promoter fused to a hygR cassette) were introduced in the *phs1*- background. Lacking carotenoid synthesis was considered to enable the detection of different pigmentation levels ranging from white to black more easily. Sequences homologous to the 5'-noncoding and the coding region of *pks1* for targeted integration (yellow / striped boxes) were attached to the expression constructs by PCR using primers with appropriate 5' overhangs. *Phs1*- protoplasts were co-transformed with a Cas9/sgRNA^{pks1}-delivering plasmid pAMA/ribo-*pks1*^{PS4} (Cas9 cutting site is highlighted with the green arrow PS4) and the seven different constructs shown (Supplementary Table 4; Figure 4). By this, the 5'-noncoding region of *pks1* (1-kb-long, considered as P*pks1*) was replaced by hygR-P*oi* or hygR only (ΔP*pks1*::*pks1*, background control). Primers used for genotyping are indicated. PCR fragments obtained with P*oi*-sFX/-hiR and *pks1*-hiR (HR at 3') of two clones per construct were sequenced to verify the absence of detrimental point mutations in *pks1* (data not shown).

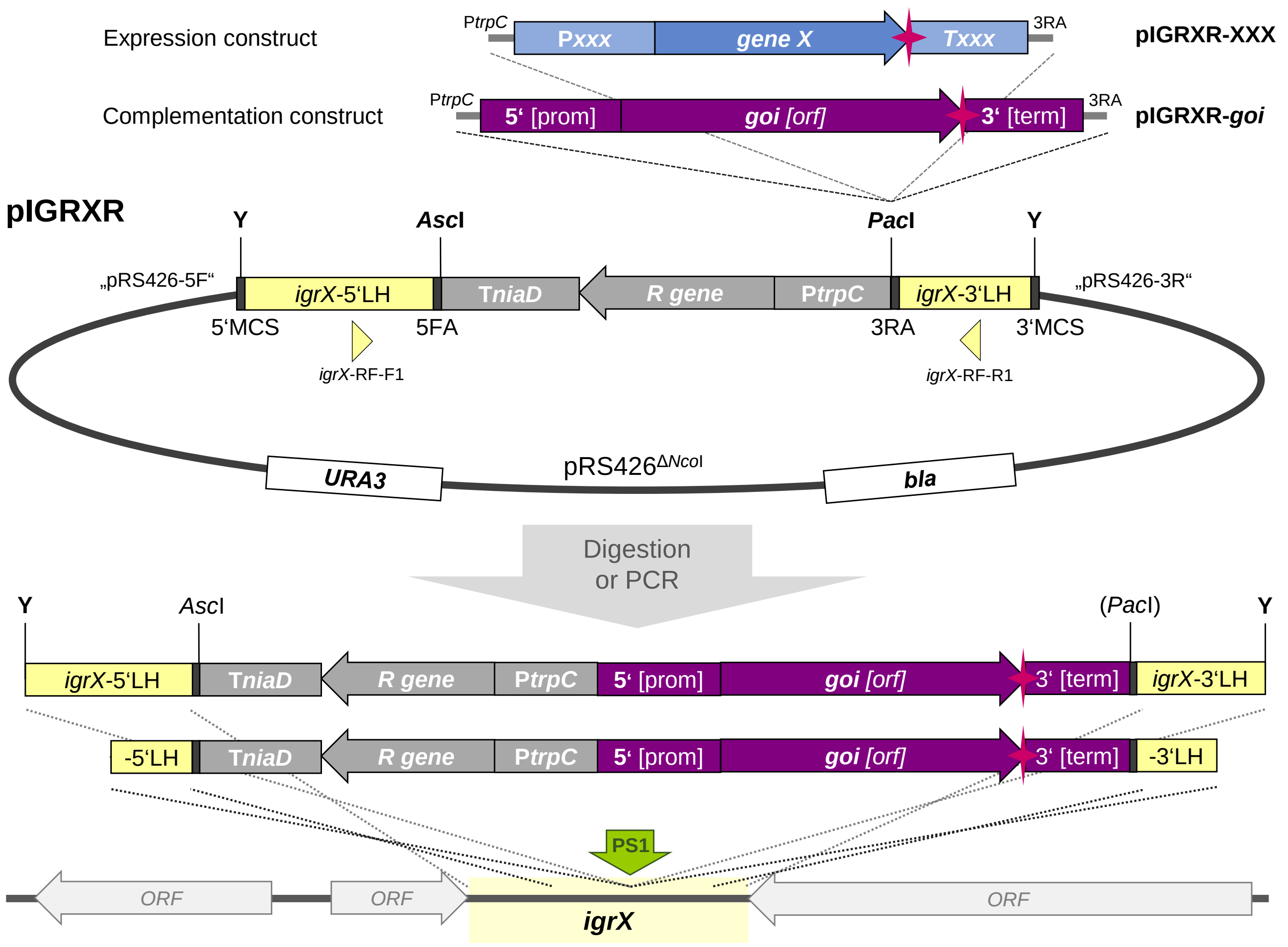

Supplementary Figure 11. pIGRXR cloning vectors for targeted insertion of expression constructs into *igr1/2*. These vectors were assembled by the yeast recombination machinery in this study (Supplementary Table 3) and contain the following modules: [1] long homologous (LH) sequences to the two chosen intergenic regions (*igrX* is *igr1* or *igr2*) that flank the site for the Cas9-induced DSB (PS1, arrow) for mediating insertion of the constructs by HR (*igr1*-5‘ – 1.185 kb, *igr1*-3‘ – 0.848 kb, *igr2*-5‘ – 0.917 kb, *igr2*-3‘ – 1.591 kb), [2] a cassette from the pNDR-XXX series consisting of P*trpC*, R (resistance) gene, and T*niaD* for mediating resistance to HYG (*hph*), NTC (*nat1*), G418 (*nptII*), GFS (*bar*) or CME (*sur*), [3] identical 5F adapter sequences with *Asc*I site (5FA), and 3R adapter sequences with *Pac*I site (3RA) for cloning, and [4] multiple cloning sites (5’MCS and 3’MCS) that contain sites for four rarely cutting restriction enzymes for isolation of large expression/complementation constructs by digestion. Both unique restriction sites in the adapter sequences can be used for cloning, i.e., for linearization of the entry plasmid for assembly with amplicons with overlapping sequences to 5FA and T*niaD* (*Asc*I) or P*trpC* and 3RA (*Pac*I) via HiFi DNA assembly or yeast recombination cloning. So far expression constructs (e.g., AGT or OCT resulting in pIGRXH-AGT and pIGRXN-OCT) and complementation constructs (gene of interest with 5’ and 3’ noncoding regions for native expression; not shown) have been inserted into pIGRXR by using the *Pac*I site. Smaller expression/complementation constructs for transformation of *K. petricola* can be also isolated from the plasmid by PCR using the primer pair *igrX*-RF-F1/*igrX*-RF-R1 (Supplementary Table 2). Then, all homologous sequences (flanks) are ~0.5 kb in length. Y – restriction enzyme(s) with restriction site in the MCS only; *goi* – gene of interest.

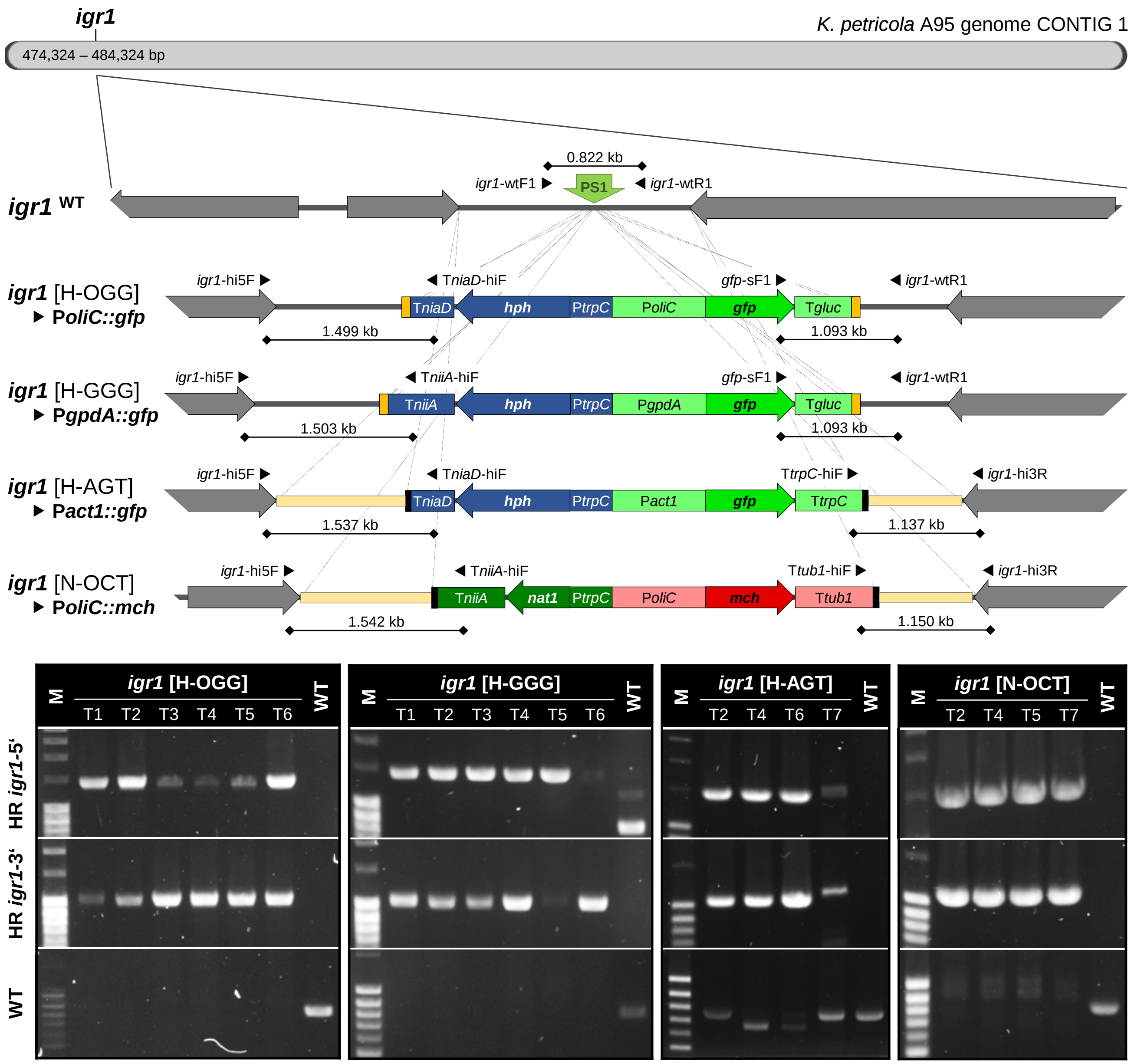

Supplementary Figure 12. Insertion of expression constructs in the intergenic region 1. The genomic locus hereafter called intergenic region 1 (*igr1*) on contig 1 of the *K. petricola* A95 genome is 2.033 kb in length (Supplementary Sequence 4). The insertion site i.e., the site for the Cas9-mediated DSB is marked by an arrow (PS1). For experimental validation of *igr1* as a suitable insertion site, four strains containing the indicated constructs – *gfp* or *mch* expression cassettes (green or red) fused to hygR or natR cassettes (blue or dark green) – were generated by co-transformation of WT:A95 protoplasts with pAMA/ribo-*igr1*^{PS1} and SH constructs (yellow boxes) amplified by PCR using pNDH-OGG and pNAH-GGG as templates or LH constructs (light yellow boxes) isolated by digestion from cloned pIGR1H-AGT or pIGR1N-OCT (Supplementary Table 3, Supplementary Table 4). Primers used for diagnostic PCR and expected fragment sizes are indicated (black triangles and lines). Diagnostic PCRs detected the desired HR events at 5’ and 3’ of the insertion site *igr1*^{PS1} revealing that all tested strains – with exception of *igr1* [H-AGT]-T7 – have inserted the expression constructs correctly.

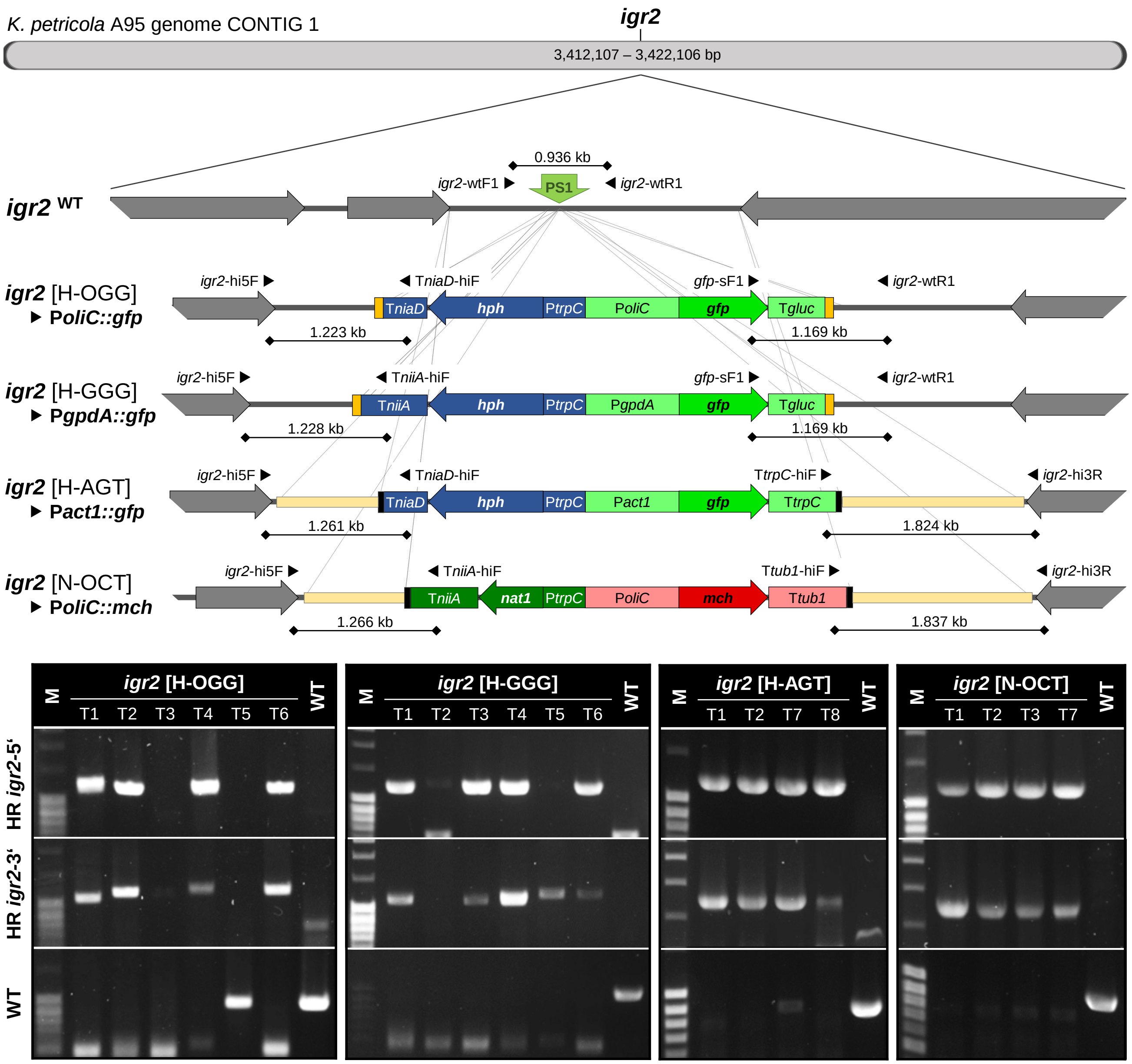

Supplementary Figure 13. Insertion of expression constructs in the intergenic region 2. The genomic locus hereafter called intergenic region 2 (*igr2*) on contig 1 of the *K. petricola* A95 genome is 2.568 kb in length (Supplementary Sequence 5). The insertion site i.e., the site for the Cas9-mediated DSB is marked by the green arrow (PS1). For experimental validation of *igr2* as a suitable insertion site, four strains containing the indicated constructs were generated by co-transformation of WT:A95 protoplasts with pAMA/ribo-*igr2*^{PS1} and SH constructs (yellow boxes) amplified by PCR from pNDH-OGG and pNAH-GGG or LH constructs (light yellow boxes) isolated by digestion from cloned pIGR2H-AGT or pIGR2N-OCT (Supplementary Table 3, Supplementary Table 4). The correct insertion of expression constructs was determined by diagnostic PCR using the indicated primers. Most of the tested strains had undergone homologous integration of the constructs.

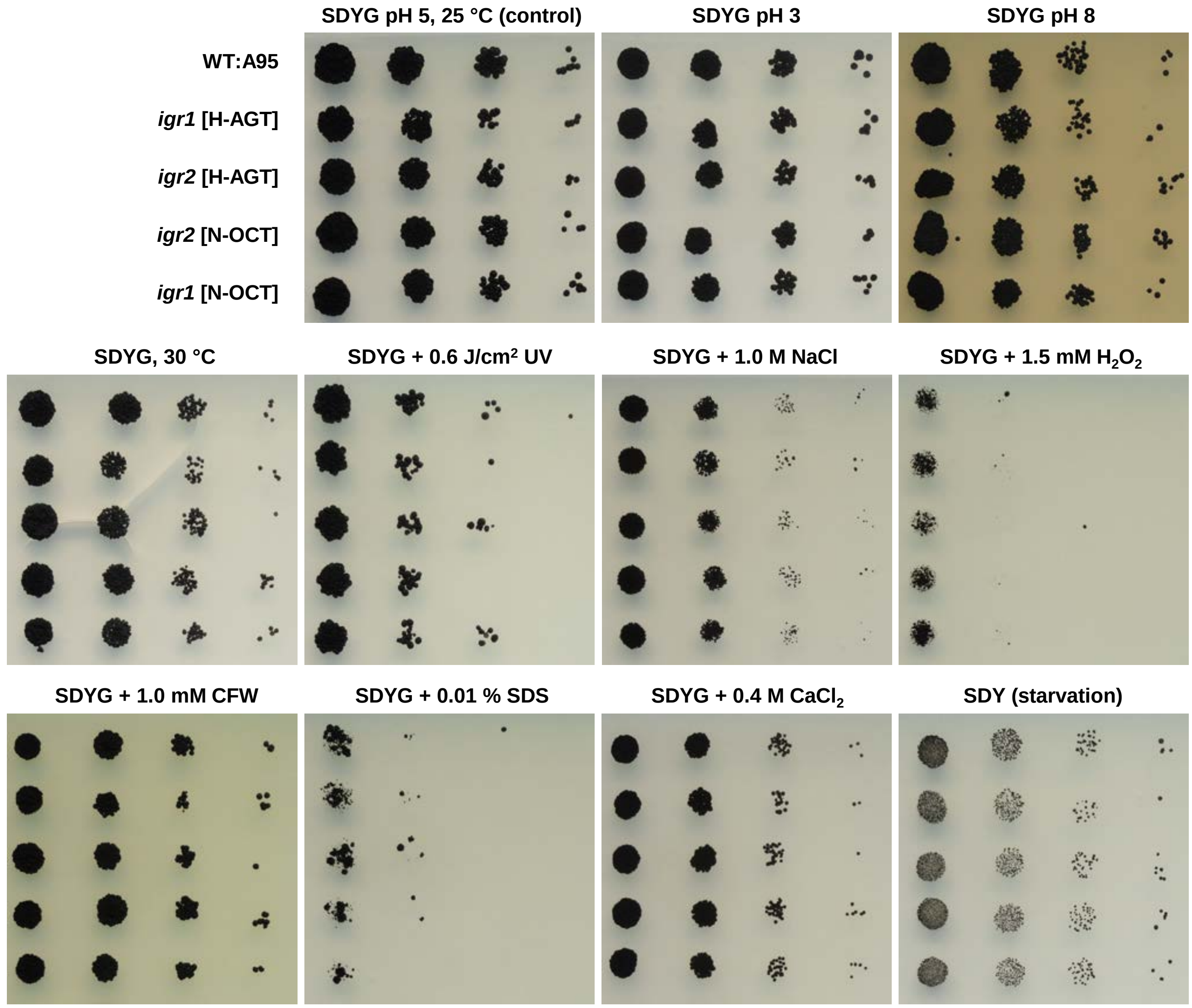

Supplementary Figure 14. Constructs in *igr1* and *irg2* do not result in obvious phenotypes. Cell suspensions (10^4, 10^3, 10^2 and 10^1 cells from the left to the right) of the *gfp*- (H-AGT) or *mcherry*- (N-OCT) expressing strains were dropped onto solid SDYG pH 5 (control), SDYG adjusted to pH 3 or pH 8 for inducing pH stress, SDYG supplemented with different stress-inducing agents [1.0 M NaCl for inducing osmotic stress, 1.5 mM hydrogen peroxide (H_2O_2) for inducing oxidative stress, 1.0 mM calcofluor white (CFW) for inducing cell wall stress, 0.01 % sodium dodecyl sulfate (SDS) for inducing membrane stress, or 0.4 M $CaCl_2$ for triggering calcium/stress signaling pathways]. For UV stress, the dropped cells on SDYG pH 5 were treated with 0.6 J/cm^2. Pictures were taken after nine days of incubation at 25 °C (control, + stresses) or 30 °C (heat stress) in darkness.

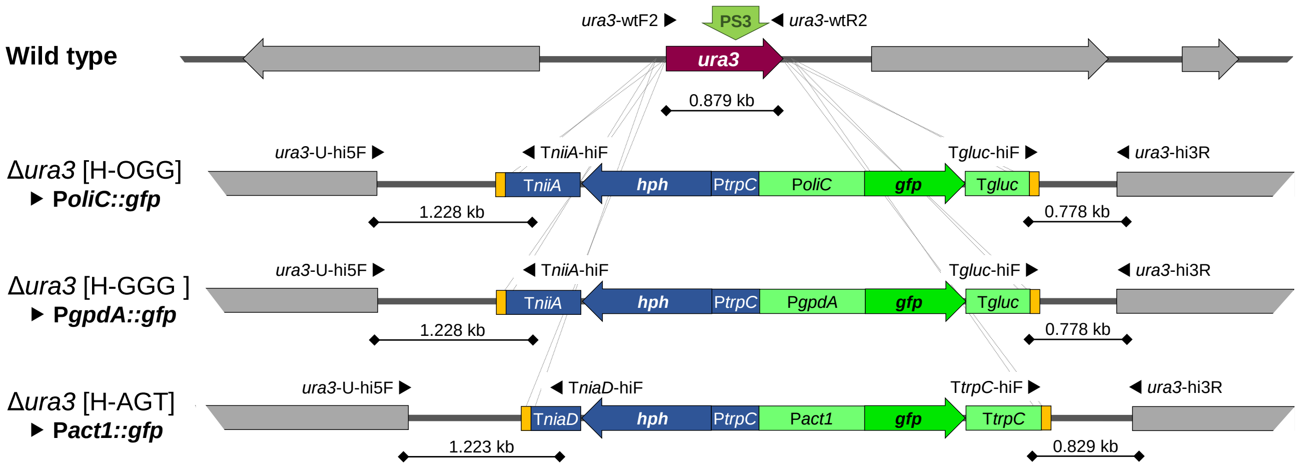

Supplementary Figure 15. Integration of *gfp* expression constructs by replacement of *ura3*. For comparing the expression of *gfp* under control of P*oliC*, P*gpdA* and P*act1* from different genomic loci, *ura3* encoding the orotidine 5'-phosphate decarboxylase (uracil synthesis) was chosen as another integration site to obtain melanized *gfp*-expressing strains. The ORF was replaced by the indicated P*oi::gfp* constructs by co-transforming WT:A95 protoplasts with pAMA/ribo-*ura3*^{PS3} and PCR-amplified expression constructs using primers with 75-bp-long homologous sequences (yellow boxes) to 5'- and 3'-noncoding regions of *ura3* and pNDH-OGG, pNAH-GGG or pNDH-AGT as template (Supplementary Table 4). The site of the Cas9-induced DSB is highlighted by the green arrow (PS3). The used primer pairs for genotyping and the expected fragment sizes are indicated. Two strains with correct replacement/integration per expression construct as determined by diagnostic PCR (data not shown) were studied by fluorescence microscopy (Figure 6C).

2 Supplementary Tables

Supplementary Table 1. *K. petricola* strains used in this study.

Strain name	GMO ID	Clone #	Genotype	Phenotype	Reference
**WT:A95**	n/a	n/a	A95	black	(Nai et al., 2013)
pks1–	**KP-0067**	O26.1	A95; *pks1*– (Δ18 bp @ ~407 bp)	pink	(Voigt et al., 2020)
phs1–	**KP-0085**	PO.22	A95; *phs1*– (premature stop @ 347 bp)	black	(Voigt et al., 2020)
pks1–/phs1–	**KP-0081**	OO.2	A95; *pks1*– (+1-bp @ 407 bp); *phs1*– (premature stop @ 347 bp)	white	(Voigt et al., 2020)
pks1–/phs1–	**KP-0201**	O1, O2, O3	A95; *pks1*– (Δ18 bp @ ~407 bp); *phs1*– (in-frame deletions @ ~345 bp)	orange	Figure 2, Figure S4
pks1–/phs1–	**KP-0205**	W1, W2, W3, W4	A95; *pks1*– (Δ3 bp @ ~407 bp); *phs1*– (premature stop @ 347 bp)	white	Figure 2, Figure S4
**Δ*pks1* [H-AGT]**	**KP-0115**	T1.1, 2.1, 3.1	A95; Δ*pks1* [(T*niaD*::*hph*::P*trpC*◀)–(▶**Pact1::gfp**::T*trpC*)]	pink, hygR; GFP	Figure 4, Figure S9
**Δ*pks1* [H-GGG]**	**KP-0116**	T1.1, 2.1, 3.1	A95; Δ*pks1* [(T*niiA*::*hph*::P*trpC*◀)–(▶**PgpdA::gfp**::T*gluc*)]	pink, hygR; GFP	Figure 4, Figure S9
**Δ*pks1* [H-OGG]**	**KP-0117**	T1.1, 2.1, 2.4	A95; Δ*pks1* [(T*niaD*::*hph*::P*trpC*◀)–(▶**PoliC::gfp**::T*gluc*)]	pink, hygR; GFP	Figure 4, Figure S9
**Δ*pks1* [H-QGT]**	**KP-0119**	T1.1, 2.1, 3.1	A95; Δ*pks1* [(T*niiA*::*hph*::P*trpC*◀)–(▶**Pgln1::gfp**::T*gluc*)]	pink, hygR; GFP	Figure 4, Figure S9
**Δ*pks1* [H-LGT]**	**KP-0120**	T1.1, 2.1, 3.1	A95; Δ*pks1* [(T*niaD*::*hph*::P*trpC*◀)–(▶**Pgal1::gfp**::T*trpC*)]	pink, hygR; GFP	Figure 4, Figure S9
**Δ*pks1* [H-NGT]**	**KP-0121**	T1.1, 2.1, 3.2	A95; Δ*pks1* [(T*niaD*::*hph*::P*trpC*◀)–(▶**PniaD::gfp**::T*trpC*)]	pink, hygR; GFP	Figure 4, Figure S9
***phs1–/Pact1::pks1* [H]**	**KP-0141**	T5, T6	A95; *phs1*– (stop @ 347 bp); ΔP*pks1* [(T*niaD*::*hph*::P*trpC*◀)–(▶**Pact1::pks1**)]	black, hygR	Figure 4, Figure S10
***phs1–/PgpdA::pks1* [H]**	**KP-0142**	T3, T4	A95; *phs1*– (stop @ 347 bp); ΔP*pks1* [(T*niiA*::*hph*::P*trpC*◀)–(▶**PgpdA::pks1**)]	black, hygR	Figure 4, Figure S10
***phs1–/PoliC::pks1* [H]**	**KP-0143**	T3, T4	A95; *phs1*– (stop @ 347 bp); ΔP*pks1* [(T*niaD*::*hph*::P*trpC*◀)–(▶**PoliC::pks1**)]	black, hygR	Figure 4, Figure S10
***phs1–/Pgln1::pks1* [H]**	**KP-0144**	T7, T8, T9	A95; *phs1*– (stop @ 347 bp); ΔP*pks1* [(T*niiA*::*hph*::P*trpC*◀)–(▶**Pgln1::pks1**)]	black, hygR	Figure 4, Figure S10
***phs1–/Pgal1::pks1* [H]**	**KP-0145**	T1, T2, T3	A95; *phs1*– (stop @ 347 bp); ΔP*pks1* [(T*niaD*::*hph*::P*trpC*◀)–(▶**Pgal1::pks1**)]	gray, hygR	Figure 4, Figure S10
***phs1–/PniaD::pks1* [H]**	**KP-0146**	T1, T5, T6	A95; *phs1*– (stop @ 347 bp); ΔP*pks1* [(T*niaD*::*hph*::P*trpC*◀)–(▶**PniaD::pks1**)]	black, hygR	Figure 4, Figure S10
***phs1–/ΔPpks1::pks1* [H]**	**KP-0147**	T1, T2, T3	A95; *phs1*– (stop @ 347 bp); ΔP*pks1* [(T*niaD*::*hph*::P*trpC*◀)]	gray, hygR	Figure 4, Figure S10
**P*gal1::pks1* [H]**	**KP-0265**	T3, T4, T5, T6	A95; ΔP*pks1* [(T*niaD*::*hph*::P*trpC*◀)–(▶**Pgal1::pks1**)]	dark pink, hygR	Figure 4, Figure S10
**ΔP*pks1::pks1* [H]**	**KP-0260**	T1, T3, T4, T5	A95; ΔP*pks1* [(T*niaD*::*hph*::P*trpC*◀)]	dark pink, hygR	Figure 4, Figure S10
***igr1* [H-AGT]**	**KP-0106**	T2, T4, T6	A95; *igr1* [(T*niaD*::*hph*::P*trpC*◀)–(▶**Pact1::gfp**::T*trpC*)]	black, hygR; GFP	Figure 6, Figure S12

Strain name	GMO ID	Clone #	Genotype	Phenotype	Reference
***igr1* [N-OCT]**	**KP-0101**	T2, T4, T5, T7	A95; *igr1* [(T*niiA*::*nat1*::P*trpC*◂)–(▸**P*oliC*::*mch***::T*tub1*)]	black, natR; GFP	Figure 6, Figure S12
***igr1* [H-GGG]**	**KP-0172**	T1, T2, T3, T4	A95; *igr1* [(T*niiA*::*hph*::P*trpC*◂)–(▸**P*gpdA*::*gfp***::T*gluc*)]	black, hygR; GFP	Figure 6, Figure S12
***igr1* [H-OGG]**	**KP-0173**	T1, T2, T3, T6	A95; *igr1* [(T*niaD*::*hph*::P*trpC*◂)–(▸**P*oliC*::*gfp***::T*gluc*)]	black, hygR; GFP	Figure 6, Figure S12
***igr2* [H-AGT]**	**KP-0094**	T1, T2, T7, T8	A95; *igr2* [(T*niaD*::*hph*::P*trpC*◂)–(▸**P*act1*::*gfp***::T*trpC*)]	black, hygR; GFP	Figure 6, Figure S13
***igr2* [N-OCT]**	**KP-0108**	T1, T2, T3, T7	A95; *igr2* [(T*niiA*::*nat1*::P*trpC*◂)–(▸**P*oliC*::*mch***::T*tub1*)]	black, natR; GFP	Figure 6, Figure S13
***igr2* [H-GGG]**	**KP-0176**	T1, T3, T4, T6	A95; *igr2* [(T*niiA*::*hph*::P*trpC*◂)–(▸**P*gpdA*::*gfp***::T*gluc*)]	black, hygR; GFP	Figure 6, Figure S13
***igr2* [H-OGG]**	**KP-0177**	T2, T4, T6	A95; *igr2* [(T*niaD*::*hph*::P*trpC*◂)–(▸**P*oliC*::*gfp***::T*gluc*)]	black, hygR; GFP	Figure 6, Figure S13
**Δ*ura3* [H-AGT]**	**KP-0148**	T1.6, T1	A95; Δ*ura3* [(T*niaD*::*hph*::P*trpC*◂)–(▸**P*act1*::*gfp***::T*trpC*)]	black, hygR, ura-; GFP	Figure 6, Figure S15
**Δ*ura3* [H-OGG]**	**KP-0149**	T6, T7, T8	A95; Δ*ura3* [(T*niaD*::*hph*::P*trpC*◂)–(▸**P*oliC*::*gfp***::T*gluc*)]	black, hygR, ura-; GFP	Figure 6, Figure S15
**Δ*ura3* [H-GGG]**	**KP-0171**	T1.5, T6	A95; Δ*ura3* [(T*niiA*::*hph*::P*trpC*◂)–(▸**P*gpdA*::*gfp***::T*gluc*)]	black, hygR, ura-; GFP	Figure 6, Figure S15
**Δ*pks1* [G]**	**KP-0192**	T1, T2, T3, T4	A95; Δ*pks1* [(T*niaD*::***nptII***::P*trpC*◂)]	pink, genR	Figure 3, Figure S7
**Δ*pks1* [P]**	**KP-0242**	T1, T2, T3, T4	A95; Δ*pks1* [(T*niaD*::***bar***::P*trpC*◂)]	pink, baR	Figure 3, Figure S7
**Δ*pks1* [S]**	**KP-0243**	T2, T4, T5, T6	A95; Δ*pks1* [(T*niaD*::***sur***::P*trpC*◂)]	pink, suR	Figure 3, Figure S7
***igr2* [G-OGG]**	**KP-0246**	T1, T2, T3, T6	A95; *igr2* [(T*niaD*::***nptII***::P*trpC*◂)–(▸P*oliC*::*gfp*::T*gluc*)]	black, genR; GFP	Figure 3, Figure S8
***igr2* [P-OGG]**	**KP-0247**	T2, T3, T4, T5	A95; *igr2* [(T*niaD*::***bar***::P*trpC*◂)–(▸P*oliC*::*gfp*::T*gluc*)]	black, baR; GFP	Figure 3, Figure S8
***igr2* [S-OGG]**	**KP-0248**	T4, T6	A95; *igr2* [(T*niaD*::***sur***::P*trpC*◂)–(▸P*oliC*::*gfp*::T*gluc*)]	black, suR; GFP	Figure 3, Figure S8
**Δ*pks1* [H-W1G]**	**KP-0182**	T1, T2, T3, T4	A95; Δ*pks1* [(T*niiA*::*hph*::P*trpC*◂)–(▸P*gpdA*::***wcl1-gfp***::T*gluc*)]	pink, hygR; nuclear GFP	Figure 5
**Δ*pks1* [N-GW2]**	**KP-0186**	T1, T2, T3, T4	A95; Δ*pks1* [(T*niaD*::*nat1*::P*trpC*◂)–(▸P*oliC*::***gfp-wcl2***::T*gluc*)]	pink, natR; nuclear GFP	Figure 5
**Δ*pks1* [H-W1M]**<br>**+ Δ*phs1* [N-GW2]**	**KP-0214**	B1-1, -2, -3, -4	A95; Δ*pks1* [(T*niiA*::*hph*::P*trpC*◂)–(▸P*gpdA*::***wcl1-mch***::T*tub1*)];<br>Δ*phs1* [(T*niaD*::*nat1*::P*trpC*◂)–(▸P*oliC*::***gfp-wcl2***::T*gluc*)]	white, hygR, natR; nuclear GFP, nuclear mCH	Figure 5
**Δ*pks1* [H-W1GC]**<br>**+ Δ*phs1* [N-GNW2]**	**KP-0215**	B2-1, -2, -3, -4	A95; Δ*pks1* [(T*niiA*::*hph*::P*trpC*◂)–(▸P*gpdA*::***wcl1-gfpC***::T*gluc*)];<br>Δ*phs1* [(T*niaD*::*nat1*::P*trpC*◂)–(▸P*gpdA*::***gfpN-wcl2***::T*trpC*)]	white, hygR, natR; nuclear GFP	Figure 5
**Δ*pks1* [H-W1GC]**<br>**+ Δ*phs1* [N-GN]**	**KP-0216**	B3-1, -2, -3	A95; Δ*pks1* [(T*niiA*::*hph*::P*trpC*◂)–(▸P*gpdA*::***wcl1-gfpC***::T*gluc*)];<br>Δ*phs1* [(T*niaD*::*nat1*::P*trpC*◂)–(▸P*gpdA*::***gfpN***::T*trpC*)]	white, hygR, natR	Figure 5
**Δ*pks1* [H-GC]**<br>**+ Δ*phs1* [N-GNW2]**	**KP-0217**	B4-1, -2, -3	A95; Δ*pks1* [(T*niiA*::*hph*::P*trpC*◂)–(▸P*oliC*::***gfpC***::T*gluc*)];<br>Δ*phs1* [(T*niaD*::*nat1*::P*trpC*◂)–(▸P*gpdA*::***gfpN-wcl2***::T*trpC*)]	white, hygR, natR	Figure 5
**Δ*pks1* [H-GC]**<br>**+ Δ*phs1* [N-GNW2]**	**KP-0218**	B5-1, -2, -3	A95; Δ*pks1* [(T*niiA*::*hph*::P*trpC*◂)–(▸P*oliC*::***gfpC***::T*gluc*)];<br>Δ*phs1* [(T*niaD*::*nat1*::P*trpC*◂)–(▸P*gpdA*::***gfpN***::T*trpC*)]	white, hygR, natR	Figure 5

Supplementary Table 2. Oligonucleotides used in this study.

Primer name	Sequence (5’➔ 3’)	Features (5’➔ 3’)
***Kppks1*-tRNA-PS2R**	ttattaagccggtgcacaga-TGCATCATCCGTGAATCGAAC	*kppks1* ^{PS2} – tRNA
***Kppks1*-tRNA-PS2F**	tctgtgcaccggcttaataa-GTTTTAGAGCTAGAAATAGCAAGTTAAAAT	*kppks1* ^{PS2} – sgRNA
***Kppks1*-AMA-PS4F**	gtccgtgaggacgaaacgagtaagctcgtc-TTGAGATTCGGAAACCGACC-gttttagagctagaaatagcaagttaaa	HH-dw – PS4 – sgRNA
***Kppks1*-AMA-PS4R**	gacgagcttactcgtttcgtcctcacggactcatcag-TTGAGA-cggtgatgtctgctcaagcg	HH-up – 6bp-PS4 – P*gpdA*
**T*niaD*-*kppks1*-SH5F**	agcgtccactctttccgcagcaagtatcccagtagaccagcagatgcttctcagcttgatcactcctgc-GCATTGGATTAATAATTGTTGCTAAGCGAG	*kppks1*-5' – T*niaD*
**T*niiA*-*kppks1*-SH5F**	ctggtggaagcgtccactctttccgcagcaagtatcccagtagaccagcagatgcttctcagcttgatcactcctgc-CAGATGCTGCTGGCAAGGTTAC	*kppks1*-5' – T*niiA*
**P*gpdA*-*kppks1*-SH5F**	ctggtggaagcgtccactctttccgcagcaagtatcccagtagaccagcagatgcttctcagcttgatcactcctgc-GTACAGTGACCGGTGACTCTTTC	*kppks1*-5' – P*gpdA*
**T*trpC*-*kppks1*-SH3R**	ttgttgaaacaggttgcagtagttgatccagacaacaccatgatatgccagtcaagtgagtaggttcgtgtggttgc-TCGAGTGGAGATGTGGAGTGGG	*kppks1*-3' – T*trpC*
**T*gluc*-*kppks1*-SH3R**	tgttgaaacaggttgcagtagttgatccagacaacaccatgatatgccagtcaagtgagtaggttcgtgtggttgc-ATCTTGTTGGGGGGAAGGGGTTG	*kppks1*-3' – T*gluc*
**T*tub1*-*kppks1*-SH3R**	tgttgaaacaggttgcagtagttgatccagacaacaccatgatatgccagtcaagtgagtaggttcgtgtggttgc-ggatccAGACAGCCAATTGAAACG	*kppks1*-3' – T*tub1*
***Kppks1*-P*act1*-SH3R**	aagacctttgtgaagaacgcccggcagtctgccgtttggtcaccgaacacgtagacttcctccatgg-GGTTGATAAATTAAGACG	*kppks1* – P*act1*
***Kppks1*-P*gpdA*-SH3R**	ctttcttgggaagacctttgtgaagaacgcccggcagtctgccgtttggtcaccgaacacgtagacttcctccat-GGAAGCTTAGATCTGTAGCTG	*kppks1* – P*gpdA*
***Kppks1*-P*oliC*-SH3R**	ctttcttgggaagacctttgtgaagaacgcccggcagtctgccgtttggtcaccgaacacgtagacttcctccat-GGTTGGATCGATTGTGATGTG	*kppks1* – P*oliC*
***Kppks1*-P*gln1*-SH3R**	ctttcttgggaagacctttgtgaagaacgcccggcagtctgccgtttggtcaccgaacacgtagacttcctccat-TGTGAATGTGGTTGTGATACGGGG	*kppks1* – P*ffgln1*
***Kppks1*-P*gal1*-SH3R**	aagacctttgtgaagaacgcccggcagtctgccgtttggtcaccgaacacgtagacttcctccat-GGTTGTACGAGTCCTAGCCTGAT	*kppks1* – P*kpgal1*
***Kppks1*-P*niaD*-SH3R**	ttgggaagacctttgtgaagaacgcccggcagtctgccgtttggtcaccgaacacgtagacttcctccat-GGTGAGAGTATGGGATAGGA	*kppks1* – P*anniaD*
***Kppks1*-P*trpC*-SH3R**	ctttgtgaagaacgcccggcagtctgccgtttggtcaccgaacacgtagacttcctccat-ACTAGTGATATTGAAGGAGCATTTTTTGGGC	*kppks1* – P*trpC*
***Kppks1*-RT5F** (SH5F)	gatcagcccttcttttgtttttctgctcgttaagaaccgcacccgaagtacgtcgacactcattcacatttcact-GCTAAGCGAGCGGGAGCTATCG	*kppks1*-5' – T*niaD*
***Kppks1*-RT3R** (SH3R)	gttgaaacaggttgcagtagttgatccagacaacaccatgatatgccagtcaagtgagtaggttcgtgtggttgc-GAATCGGGAATGCGGCTCCACAG	*kppks1*-3' – P*oliC*
***Kppks1*-oligo-2.7**	GCCTAATACAGATGCTAGACTGGTCGGTCTGTGCACCGGCATAACGGCGGCAGCTGTGTCTTGCGCCGACTCCTTAACCG	Δ3 bp of *kppks1*-ORF
***Kppks1*-SH-hi5F**	GGTTGTCGGCAGTGATACGACAAG	*kppks1*-5’
***Kppks1*-SH-hi3R**	GAGTTAGATTCGAGACACTCCACCAG	*kppks1*-3‘
***Kppks1*-WT-F1**	GGTGAGGCTGTATCTGGTGT	*kppks1*-ORF
***Kppks1*-WT-R2**	GTCCGAGACGCCGTTGATGCATG	*kppks1*-ORF
***Kppks1*-hiR**	GCTCCAGCTCTGAAAGCAATGCG	*kppks1*-ORF
***Kppks1*-PS1-sF3**	GCTGGTAGAGTACGCTATATCCGC	*kppks1*-5’

Primer name	Sequence (5'→ 3')	Features (5'→ 3')
***Kppks1*-PS1-sF2**	GGCATCATGCCCAGGTCGG	*kppks1*-5'
***Kppks1*-RNAi-R1**	CCTTCTCGGAGTGCAAGTGTGC	*kppks1*-ORF
***Kpphs1*-tRNA-PS1R**	caggcggataagctgcaccg-TGCATCATCCGTGAATCGAAC	*kpphs1* PS1 – tRNA
***Kpphs1*-tRNA-PS1F**	cggtgcagcttatccgcctg-GTTTTAGAGCTAGAAATAGCAAGTTAAAAT	*kpphs1* PS1 – sgRNA
***TniaD-kpphs1*-SH5F**	tactcaccaccaaagccgtgattcttgatttcgcgtagacgtctgcacatcaacttgcgcatcgcgatcc-GCATTGGATTAATAATTGTTGCTAAGCGAG	*kpphs1*-5' – *TniaD*
***TniiA-kpphs1*-SH5F**	gatcacgttactcaccaccaaagccgtgattcttgatttcgcgtagacgtctgcacatcaacttgcgcatcgcgatc-AGATGCTGCTGGCAAGGTTAC	*kpphs1*-5' – *TniiA*
***PgpdA-kpphs1*-SH5F**	gatcacgttactcaccaccaaagccgtgattcttgatttcgcgtagacgtctgcacatcaacttgcgcatcgcgatc-GTACAGTGACCGGTGACTCTTTC	*kpphs1*-5' – *PgpdA*
***TtrpC-kpphs1*-SH3R**	gcgcggcgactgcattactattcttacgttgaaggcccgctacacaagcagaagtcgagagccgttagacgcagat-TCGAGTGGAGATGTGGAGTGGG	*kpphs1*-3' – *TtrpC*
***Tgluc-kpphs1*-SH3R**	gcgcggcgactgcattactattcttacgttgaaggcccgctacacaagcagaagtcgagagccgttagacgcagat-ATCTTGTTGGGGGGAAGGGGTTG	*kpphs1*-3' – *Tgluc*
***Ttub1-kpphs1*-SH3R**	gcgcggcgactgcattactattcttacgttgaaggcccgctacacaagcagaagtcgagagccgttagacgcagat-ggatccAGACAGCCAATTGAAACG	*kpphs1*-3' – *Ttub1*
***Kpphs1*-RT5F** (SH5F)	tcacgttactcaccaccaaagccgtgattcttgatttcgcgtagacgtctgcacatcaacttgcgcatcgcgatc-GCTAAGCGAGCGGGAGCTATCG	*kppks1*-5' – *TniaD*
***Kpphs1*-RT3R** (SH3R)	cgcggcgactgcattactattcttacgttgaaggcccgctacacaagcagaagtcgagagccgttagacgcagat-GAATCGGGAATGCGGCTCCACAG	*kppks1*-3' – *PoliC*
***Kpphs1*-oligo-1**	ACGTATATAAACGCTAATACGGTGCAGCTTATCCGCCTTAAGCTGTCATCGGCCCTGCCTGGTTCGATGTACCTGCCGAG	STOP in *kpphs1*-ORF
***Kpphs1*-hi5F**	GCGAGGTTCTGGTAGTGCAGCTG	*kpphs1*-5'
***Kpphs1*-SH-hi5F**	GGTGCTAAGCTTTGCCAGCCTCG	*kpphs1*-5'
***Kpphs1*-hi3R**	GCGCTCTGACATTAGCCAGTCTCC	*kpphs1*-3'
***Kpphs1*-WT-F**	GTTCGATGTACCTGCCGAGGAGTTG	*kpphs1*-ORF
***Kpphs1*-WT-R**	CTCGGCTGCACTAGCAGCATCATC	*kpphs1*-ORF
***Kpura3*-AMA-PS3-F**	gtccgtgaggacgaaacgagtaagctcgtc-TACACCTCGATCCGTAGTAC-gttttagagctagaaatagcaagttaaa	HH-dw – PS3 – sgRNA
***Kpura3*-AMA-PS3-R**	gacgagcttactcgtttcgtcctcacggactcatcag-TACACC-cggtgatgtctgctcaagcg	HH-up – 6bp-PS3 – *PgpdA*
***TniaD-kpura3*-SH5F**	ccagacctgcacagatcactcacgtcagtgggaggatcgaccaacatttacacataaggtgtagccgagc-GCATTGGATTAATAATTGTTGCTAAGCGAG	*kpura3*-5' – *TniaD*
***TniiA-kpura3*-SH5F**	tctacgcaccagacctgcacagatcactcacgtcagtgggaggatcgaccaacatttacacataaggtgtagccgagcc-AGATGCTGCTGGCAAGGTTAC	*kpura3*-5' – *TniiA*
***TtrpC-kpura3*-SH3R**	tcatgaatcctactcaaatcatgaaacaatgtgcggataacccaccgaacatgcaactctggccaaagggactggcgt-TCGAGTGGAGATGTGGAGTGGG	*kpura3*-3' – *TtrpC*
***Tgluc-kpura3*-SH3R**	catgaatcctactcaaatcatgaaacaatgtgcggataacccaccgaacatgcaactctggccaaagggactggcgt-ATCTTGTTGGGGGGAAGGGGTTG	*kpura3*-3' – *Tgluc*
***Ttub1-kpura3*-SH3R**	atgaatcctactcaaatcatgaaacaatgtgcggataacccaccgaacatgcaactctggccaaagggactggcgt-GGATCCAGACAGCCAATTGAAACG	*kpura3*-3' – *Ttub1*
***Kpura3*-WT-F2**	GAGTGCCGTACCGTTCGCGAAG	*kpura3*-ORF
***Kpura3*-WT-R2**	CATTCTGGTACTCCTTGACCGC	*kpura3*-ORF
***Kpura3*-5U-hi5F**	GCGCTCTGAGTAGATTTGGTCTCG	*kpura3*-5'

Primer name	Sequence (5'→ 3')	Features (5'→ 3')
***Kpura3*-hi3R**	CCATGTCCGCTGACACCATTCG	*kpura3*-3'
***Kpigr1*-AMA-PS1F**	gtccgtgaggacgaaacgagtaagctcgtc-ACCGTACTCTCCGCGTAGAC-gttttagagctagaaatagcaagttaaa	HH-dw – *kpigr1* ^{PS1} – sgRNA
***Kpigr1*-AMA-PS1R**	gacgagcttactcgtttcgtcctcacggactcatcag-ACCGTA-cggtgatgtctgctcaagcg	HH-up – 6bp-*igr1* ^{PS1} – *PgpdA*
***Kpigr1*-tRNA-PS1F**	accgtactctccgcgtagac-GTTTTAGAGCTAGAAATAGCAAGTTAAAAT	*kpigr1* ^{PS1} – sgRNA
***Kpigr1*-tRNA-PS1R**	gtctacgcggagagtacggt-TGCATCATCCGTGAATCGAAC	*kpigr1* ^{PS1} – tRNA
***Kpigr1*-5F**	gttgggtaacgccagggttttcccagtcacga-atttaaatgggcccgtttaaacgctagc-CCGCCAGGTTGAACTCATGCTTAC	pRS426 – MCS – *kpigr1*
***Kpigr1*-5R**	gtcttaagatcgcgaacaagacgtcgctcc-TACGCGGAGAGTACGGTCCGTT	5R-adapter – *kpigr1*
***Kpigr1*-3F**	ggtattccaattccattcgggaacaccacc-GACTGGTCGTCGAGCTAGTCCTG	3R-adapter – *kpigr1*
***Kpigr1*-3R**	tgtgagcggataacaatttcacacaggaaaca-gggcccgctagcatttaaatgtttaaac-GGTGTCCAGCTCAAAGTGGCGC	pRS426 – MCS – *kpigr1*
***Kpigr1*-RF-F1**	GCGGTTACCGAAGGATCTTTGCAAG	*kpigr1*-5'
***Kpigr1*-RF-R1**	GTTCTGTAATCTTCGCTCGTACAACGG	*kpigr1*-3'
***TniaD-kpigr1*-SH5F**	ctgccgctcggaacgttccatccggtcctgtctgatccatccactcccaacggaccgtactctccgcgta-GCATTGGATTAATAATTGTTGCTAAGCGAG	*kpigr1*-5' – T*niaD*
***TtrpC-kpigr1*-SH3R**	gtagggcatggcggtgagtgaggctcaacggtgatcgcgatcgatctggcaaaacaggactagctcgacgaccagtc-TCGAGTGGAGATGTGGAGTGGG	*kpigr1*-3' – T*trpC*
***TniiA-kpigr1*-SH5F**	ctgccgctcggaacgttccatccggtcctgtctgatccatccactcccaacggaccgtactctccgcgta-AGATGCTGCTGGCAAGGTTAC	*kpigr1*-5' – T*niiA*
***Tgluc-kpigr1*-SH3R**	gtagggcatggcggtgagtgaggctcaacggtgatcgcgatcgatctggcaaaacaggactagctcgacgaccagtc-ATCTTGTTGGGGGGAAGGGGTTG	*kpigr1*-3' – T*gluc*
***Ttub1-kpigr1*-SH3R**	tagggcatggcggtgagtgaggctcaacggtgatcgcgatcgatctggcaaaacaggactagctcgacgaccagtc-GGATCCAGACAGCCAATTGAAACG	*kpigr1*-3' – T*tub1*
***Kpigr1*-hi5F**	GATGGGCCAGGCGTTGTTCATTC	*kpigr1*
***Kpigr1*-hi3R**	GGCAGCTGCTAGACGAATTAGTGC	*kpigr1*
***Kpigr1*-WT-F1**	GCCTCTCAAATTCCTCCGCGCAG	*kpigr1*
***Kpigr1*-WT-R1**	GACTCGTGAAGAAGCAGCCCACAC	*kpigr1*
***Kpigr2*-AMA-PS1F**	gtccgtgaggacgaaacgagtaagctcgtc-AGCTGCTAGTCCATAACTCA-gttttagagctagaaatagcaagttaaa	HH-dw – *kpigr2* ^{PS1} – sgRNA
***Kpigr2*-AMA-PS1R**	gacgagcttactcgtttcgtcctcacggactcatcag-AGCTGC-cggtgatgtctgctcaagcg	HH-up – 6bp-*igr2* ^{PS1} – *PgpdA*
***Kpigr2*-tRNA-PS1F**	agctgctagtccataactca-GTTTTAGAGCTAGAAATAGCAAGTTAAAAT	*kpigr2* ^{PS1} – sgRNA
***Kpigr2*-tRNA-PS1R**	tgagttatggactagcagct-TGCATCATCCGTGAATCGAAC	*kpigr2* ^{PS1} – tRNA
***Kpigr2*-5F**	gttgggtaacgccagggttttcccagtcacga-ccgcgggtttaaacggtaccatttaaat-ATGGTCTGCGCCAACGACAAAGAC	pRS426 – MCS – *kpigr2*
***Kpigr2*-5R**	gtcttaagatcgcgaacaagacgtcgctcc-GTTATGGACTAGCAGCTATAGTGAGAGTCG	5R-adapter – *kpigr2*
***Kpigr2*-3F**	ggtattccaattccattcgggaacaccacc-TCACGGTGATGACTGTCTGACGC	3R-adapter – *kpigr2*
***Kpigr2*-3R**	tgtgagcggataacaatttcacacaggaaaca-ggtaccccgcggatttaaatgtttaaac-CACTGAGGGTGGTGTTGTCTGCC	pRS426 – MCS – *kpigr2*

Primer name	Sequence (5'→ 3')	Features (5'→ 3')
***Kpigr2*-RF-F1**	GCCTGCCAGAGTTCGGATTACCAG	*kpigr2*-5'
***Kpigr2*-RF-R1**	GACCTGAGCTACAGGCCTCGATCG	*kpigr2*-3'
***TniaD-kpigr2*-SH5F**	cgaacactaggcgatgccgacaacacaacagagtcaccgtcgactctcactatagctgctagtccataac-GCATTGGATTAATAATTGTTGCTAAGCGAG	*kpigr2*-5' – T*niaD*
***TtrpC-kpigr2*-SH3R**	ttgattgtgcacctacggaaagaactagaaatacatcctgttggccgagctggtgcgtcagacagtcatcaccgtga-TCGAGTGGAGATGTGGAGTGGG	*kpigr2*-3' – T*trpC*
***TniiA-kpigr2*-SH5F**	cgaacactaggcgatgccgacaacacaacagagtcaccgtcgactctcactatagctgctagtccataac-AGATGCTGCTGGCAAGGTTAC	*kpigr2*-5' – T*niiA*
***Tgluc-kpigr2*-SH3R**	ttgattgtgcacctacggaaagaactagaaatacatcctgttggccgagctggtgcgtcagacagtcatcaccgtga-ATCTTGTTGGGGGGAAGGGGTTG	*kpigr2*-3' – T*gluc*
***Ttub1-kpigr2*-SH3R**	tgattgtgcacctacggaaagaactagaaatacatcctgttggccgagctggtgcgtcagacagtcatcaccgtga-ggatccAGACAGCCAATTGAAACG	*kpigr2*-3' – T*tub1*
***Kpigr2*-hi5F**	GAGCGATGTCGTCTCGGAAGATTAC	*kpigr2*
***Kpigr2*-hi3R**	GAGCAGCTAAGGTTGAGGAGCCC	*kpigr2*
***Kpigr2*-WT-F1**	GGCAACTCAGACGCAATGGAGCC	*kpigr2*
***Kpigr2*-WT-R1**	CCTGAGCTACAGGCCTCGATCG	*kpigr2*
**P*trpC*-3RAM-R2**	ggtggtgttcccgaatggaattggaatacc-ttaattaa-GATATTGAAGGAGCATTTTTTGGGCTTG	3R-adapter – *Pac*I – P*trpC*
**T*bcniiA*-5FAM-R**	ggagcgacgtcttgttcgcgatcttaagac-ggcgcgcc-CAGATGCTGCTGGCAAGGTTAC	5F-adapter – *Asc*I – T*niiA*
**T*bcniaD*-5FAM-R**	ggagcgacgtcttgttcgcgatcttaagac-ggcgcgcc-GCATTGGATTAATAATTGTTGCTAAGCGAG	5F-adapter – *Asc*I – T*niaD*
**T*trpC*-3RAM-R**	ggtggtgttcccgaatggaattggaatacc-ttaattaa-TCGAGTGGAGATGTGGAGTGGG	3R-adapter – *Pac*I –T*trpC*
**T*gluc*-3RAM-R**	ggtggtgttcccgaatggaattggaatacc-ttaattaa-ATCTTGTTGGGGGGAAGGGGTTG	3R-adapter – *Pac*I –T*gluc*
**T*tub*-3RAM-R**	ggtggtgttcccgaatggaattggaatacc-ttaattaa-AGACAGCCAATTGAAACGTTCTGAC	3R-adapter – *Pac*I –T*tub1*
**5F-adapter-F**	GCGACGTCTTGTTCGCGATCTTAAG	5F-adapter
**3R-adapter-R**	GGTGGTGTTCCCGAATGGAATTGGAATAC	3R-adapter
**P*kpgal1*-P*trpC*-F1**	caagcccaaaaaatgctccttcaatatc-GTTTGGCGGTAGTGTGGCGGG	P*trpC* – *kpgal1*-5'
**P*kpgal1*-*ogfp*-R1**	cttacctcacccttggaaaccatgg-TTGTACGAGTCCTAGCCTGATATTG	*kpgal1*-5'
**P*ffgln1*-P*trpC*-F**	gcccaaaaagtgctccttcaatgtcactagt-GGCTCCGTAAGCGGAGCAAAGCG	P*trpC* – *ffgln1*-5'
**P*ffgln1*-*gfp*-R**	tacttacctcacccttggaaaccat-TGTGAATGTGGTTGTGATACGGGG	*gfp* – *ffgln1*-5'
**P*ffgln1*-sF1**	GTCGAAGTATCTTCCCTGTGCGTG	*ffgln1*-5'
**P*kpgal1*-sF1**	ggctcatccctactccgacctcg	*kpgal1*-5'
**P*anniaD*-P*trpC*-F1**	caagcccaaaaaatgctccttcaatatc-GATGGCGGGCGCGGTGATTGAG	P*trpC* – *anniaD*-5'
**P*anniaD*-*ogfp*-R1**	cttacctcacccttggaaaccatgg-TGAGAGTATGGGATAGGAAAATAATATAGAG	*gfp* – *anniaD*-5'
**P*anniaD*-sF1**	GCAGTGACTGTACACTATGAGCGG	*anniaD*-5'

Primer name	Sequence (5'➔ 3')	Features (5'➔ 3')
***Kpwcl1*-*PoliC*-F**	ccatcacatcacaatcgatccaacc-ATGAACGGCATGTCGAATATGTAC	*PoliC* – *kpwcl1*
***Kpwcl1*-*gfp*-R**	tacttacctcacccttggaaaccat-TGTAGGTAACACGTCAGATTGAGG	*Gfp* – linker /Δstop – *kpwcl1*
***Kpwcl1*-OGCGc-R**	cgttcgggatcttgcaggccgggcg-TGTAGGTAACACGTCAGATTGAGG	Linker – Δstop – *kpwcl1*
***Kpwcl1*-*PgpdA*-F**	tgactaacagctacagatctaagctt-ATGAACGGCATGTCGAATATGTAC	*PgpdA* – NcoI – *kpwcl1*
***Kpwcl2*-*gfp*-F**	cttgggaatggatgaactttacaaa-ATGGATCCTTACAATCAAAACCCTG	*Gfp* (Δstop) – *kpwcl2*
***Kpwcl2*-AGNTn-F**	aggcggttcaggcggaggtggatct-ATGGATCCTTACAATCAAAACCCTG	Linker – *kpwcl2*
***Kpwcl2*-*TtrpC*-R**	ttgttgacatggagctattaaatca-TCAATGGCTACTACCAGTTGAGTG	*TtrpC* – *kpwcl2*
***Kpwcl2*-*Tgluc*-R**	taatcatacatcttatctacatacg-TCAATGGCTACTACCAGTTGAGTG	*Tgluc* – *kpwcl2*
***PgpdA*-S-*PtrpC*-F**	gcccaaaaaatgctccttcaatatc-actagt-ACAGTGACCGGTGACTCTT	*PtrpC* – *SpeI* – *PgpdA*
***PgpdA*-ATG-R**	CATAAGCTTAGATCTGTAGCTGTTAGTC	*PgpdA*
***PgpdA*-11aalinker-R**	gagcggcggccgctgagctccatgg-AAGCTTAGATCTGTAGCTGTTAGTC	*Linker gfpN* – NcoI – *PgpdA*
***MCh*-*NcoI*-*PgpdA*-F**	cttacctcgcccttgcttaccatgg-AAGCTTAGATCTGTAGCTGTTAGTC	*mch* – NcoI – *PgpdA*
***Gfp*-*NcoI*-*PgpdA*-F**	cttacctcacccttggaaaccatgg-AAGCTTAGATCTGTAGCTGTTAGTC	*ogfp* – NcoI – *PgpdA*
***NptII*-*PtrpC*-F1**	acttacctattctacctaagcatctc-ATGATTGAACAAGATGGATTGCAC	*PtrpC* – *nptII*
***NptII*-*TniaD*-R1**	tgatgctttggtttagggttaggccc-TCAGAAGAACTCGTCAAGAAGGCG	*TniaD* – *nptII*
***Bar*-*PtrpC*-F1**	acttacctattctacctaagcatctc-ATGAGCCCAGAACGACGCCCGG	*PtrpC* – *bar*
***Bar*-*TniaD*-R1**	tgatgctttggtttagggttaggccc-CTAAATCTCGGTGACGGGCAGG	*TniaD* – *bar*
***Sur*-*PtrpC*-F1**	acttacctattctacctaagcatctc-ATGCTTCGTACTGTTGGCCGCAAA	*PtrpC* – *sur*
***Sur*-*TniaD*-R1**	tgatgctttggtttagggttaggccc-TTAACCGTGCAGGCCATTCGTCCT	*TniaD* – *sur*
***PgpdA*-pRS436-5F**	gccagggttttcccagtcacgaccgcg-GTACAGTGACCGGTGACTCTTTC	pRS426-5F – *PgpdA*
***TniaD*-*Tgluc*-F**	caacccccttccccccaacaaga-ttaattaa-GCATTGGATTAATAATTGTTGCTAAG	*Tgluc* – *PacI* – *TniaD*
***PoliC*-pRS426-3R**	gataacaatttcacacaggaaacagc-CGAATCGGGAATGCGGCTCCACAG	pRS426-3F – *PoliC*
***Tgluc*-R1**	GATCTTGTTGGGGGGAAGGGGTTGTCAAATC	*Tgluc*
**pRS426-5F-REV**	CGTCGTGACTGGGAAAACCCTGGCGTTAC	pRS426
**pRS426-3R-FOR**	GCTGTTTCCTGTGTGAAATTGTTATCCGC	pRS426
**pRS426-s3R2**	CATTAATGCAGCTGGCACGACAGG	pRS426
**pRS426-s5F2**	GTAGCGGTCACGCTGCGCGTAACC	pRS426
**pFC334-F1**	GGTCATAGCTGTTTCCGCTGA	pFC334

Primer name	Sequence (5’➔ 3’)	Features (5’➔ 3’)
**pFC334-R1**	TGATTCTGCTGTCTCGGCTG	pFC334
***PgpdA*-sF1**	GTTGACAAGGTCGTTGCGTCAG	*PgpdA*
***PafU3*-sF1**	GCTTGAGGTTAGCGCACTCGCTAG	*PafU3*
***Pact1*-sF1**	GCGGCCCACATTCAAACTTCGTG	*Pact1*
***PoliC*-sF2**	GGGAGACGTATTTAGGTGCTAGGG	*PoliC*
***Ptef1*-sR1**	CGTTCGAGAGCATGATCAGCAC	*Ptef1*
***PtrpC*-hiR**	GTGCTCACCGCCTGGACGACTAAACC	*PtrpC*
***TniiA*-hiF**	GTCATGCGTAGGGCACCGGTAGG	*TniiA*
***TniaD*-hiF**	GGTGCCAGATGTATCAGTGAGTCTG	*TniaD*
***Tgluc*-hiF**	CATACGTACATCTGATTTGACAACC	*Tgluc*
***Ttub1*-hiF**	CCACTTCCATACATGCAACACTGC	*Ttub1*
***TtrpC*-hiF**	ACCCAGAATGCACAGGTACACTTG	*TtrpC*
***Hph*-hiF**	GTCTGGACCGATGGCTGTGTAGAAG	*hph*
***Hph*-hiR**	GACAGACGTCGCGGTGAGTTCAG	*hph*
***Nat1*-hiF**	CGGCGAGCAGGCGCTCTACATGAGC	*nat1*
***Nat1*-hiR**	GTACCGGTAAGCCGTGTCGTCGAG	*nat1*
***NptII*-hiR**	GCCGAATAGCCTCTCCACCCAAG	*nptII*
***NptII*-hiF**	GCCTTCTATCGCCTTCTTGACGAG	*nptII*
***Bar*-hiR**	GAAGTTGACCGTGCTTGTCTCGATG	*bar*
***Bar*-hiF**	GAACTGGCATGACGTGGGTTTCTG	*bar*
***Sur*-hiR**	GCTTGAGAGTCGAGATGGTTCGTG	*sur*
***Sur*-hiF**	GCACGAGTTCATCACGTTTGATGC	*sur*

Sequence (5’➔ 3’): lowercase letters – 5’ overhangs for cloning (NEBuilder HiFi assembly or recombination in yeast) and for mediating homologous recombination in *K. petricola*; uppercase letters – 3’ part of oligonucleotides binding to the template. Parts of the oligonucleotides are separated by hyphen and are further described as features. Names of two-part oligonucleotides start with the binding site, followed by the 5’ overhang.

Supplementary Table 3. Plasmids cloned in this study.

**Plasmid name** (size) [ID]	**Entry plasmid**	**Amplicon 1**	**Amplicon 2**	**Amplicon 3**	**Method**
**pAMA/tRNA-*kppks1*** ^{PS2}<br>(16.449 kb) [pEC0122]	**pFC332**/<br>*Pac*I	***kppks1*** ^{PS2} **tRNA-A** (0.589 kb)<br>primers: pFC334-F1/*kppks1*-tRNA-PS2R<br>template: pFC902	***kppks1*** ^{PS2} **tRNA-B** (0.427 kb)<br>primers: *kppks1*-tRNA-PS2F/pFC334-R1<br>template: pFC902	n/a	HiFi
**pAMA/tRNA-*kpphs1*** ^{PS1}<br>(16.449 kb) [pEC0123]	**pFC332**/<br>*Pac*I	***kpphs1*** ^{PS1} **tRNA-A** (0.589 kb)<br>primers: pFC334-F1/*kpphs1*-tRNA-PS2R<br>template: pFC902	***kpphs1*** ^{PS1} **tRNA-B** (0.427 kb)<br>primers: *kpphs1*-tRNA-PS2F/pFC334-R1<br>template: pFC902	n/a	HiFi
**pAMA/tRNA-*kppks1*** ^{PS2}<br>**-*kpphs1*** ^{PS1}<br>(16.670 kb) [pEC0124]	**pFC332**/<br>*Pac*I	***kppks1*** ^{PS2} **tRNA-A** (0.589 kb)<br>primers: pFC334-F1/*kppks1*-tRNA-PS2R<br>template: pFC902	***kppks1*** ^{PS2}**/*phs1*** ^{PS1} **tRNA-C** (0.191 kb)<br>primers: *pks1*-tRNA-PS2F/*phs1*-tRNA-PS1R<br>template: pFC902	***kpphs1*** ^{PS1} **tRNA-B** (0.427 kb)<br>primers: *kpphs1*-tRNA-PS2F/pFC334-R1<br>template: pFC902	HiFi
**pNAH-GGG**<br>(11.022 kb) [pEC0046]	**pNAH-OGG**/<br>*Nco*I + *Spe*I	**P*gpdA*** (0.840 kb)<br>primers: P*gpdA*-P*trpC*-F/*mCh*-P*gpdA*-R<br>template: pHR-*gfp*-natR	n/a	n/a	YRC
**pNAN-GGG**<br>(10.555 kb) [pEC0120]	**pNAN-OGG**/<br>*Nco*I + *Spe*I	**P*gpdA*** (0.871 kb)<br>primers: P*gpdA*-P*trpC*-F/*gfp*-P*gpdA*-F<br>template: pHR-*gfp*-natR	n/a	n/a	YRC
**pNAH-GCT**<br>(11.217 kb) [pEC0047]	**pNAH-OCT**/<br>*Nco*I + *Spe*I	**P*gpdA*** (0.840 kb)<br>primers: P*gpdA*-P*trpC*-F/*gfp*-P*gpdA*-R<br>template: pHR-*gfp*-natR	n/a	n/a	YRC
**pNAN-GCT**<br>(17.327 kb) [pEC0121]	**pNAN-OCT**/<br>*Nco*I + *Spe*I	**P*gpdA*** (0.871 kb)<br>primers: P*gpdA*-P*trpC*-F/*mCh*-P*gpdA*-F<br>template: pHR-*gfp*-natR	n/a	n/a	YRC
**pNDG-OGG**<br>(10.851 kb) [pEC0119]	**pNDN-OGG**/<br>*Spe*I + *Kpn*I	***nptII*** (0.847 kb)<br>primers: *nptII*-P*trpC*-F1/-T*niaD*-R1<br>template: pKS-Gen	n/a	n/a	YRC
**pNDP-OGG**<br>(10.608 kb) [pEC0127]	**pNDN-OGG**/<br>*Spe*I + *Kpn*I	***bar*** (0.594 kb)<br>primers: *bar*-P*trpC*-F1/-T*niaD*-R1<br>template: pCB1524	n/a	n/a	YRC
**pNDS-OGG**<br>(12.530 kb) [pEC0128]	**pNDN-OGG**/<br>*Spe*I + *Kpn*I	***sur*** (2.475 kb)<br>primers: *sur*-P*trpC*-F1/-T*niaD*-R1<br>template: pCB1532	n/a	n/a	YRC
**pH-GGG**<br>(9.483 kb) [pEC0188]	**pRS426** $^{\Delta NcoI}$/<br>*Eco*RI + *Xho*I	**[(P*gpdA*::*gfp*::T*gluc*)]** (2.182 kb)<br>primers: P*gpdA*-pRS426-5F/T*gluc*-R1<br>template: pNAH-GGG	**[(P*trpC*::*hph*::T*niaD*)]** (1.845 kb)<br>primers: T*niaD*-T*gluc*-F/P*oliC*-pRS426-3R<br>template: pNDH-OGG	n/a	YRC
**pN-GGG**<br>(9.015 kb) [pEC0189]	**pRS426** $^{\Delta NcoI}$/<br>*Eco*RI + *Xho*I	**[(P*gpdA*::*gfp*::T*gluc*)]** (2.182 kb)<br>primers: P*gpdA*-pRS426-5F/T*gluc*-R1<br>template: pNAH-GGG	**[(P*trpC*::*nat1*::T*niaD*)]** (1.345 kb)<br>primers: T*niaD*-T*gluc*-F/P*oliC*-pRS426-3R<br>template: pNDN-OGG	n/a	YRC

**Plasmid name** (size) [ID]	**Entry plasmid**	**Amplicon 1**	**Amplicon 2**	**Amplicon 3**	**Method**
**pG-GGG**<br>(9.240 kb) [pEC0190]	**pRS426**$^{\Delta NcoI}$/<br>*Eco*RI + *Xho*I	**[(P*gpdA::gfp::*T*gluc*)]** (2.182 kb)<br>primers: P*gpdA*-pRS426-5F/T*gluc*-R1<br>template: pNAH-GGG	**[(P*trpC::nptII::*T*niaD*)]** (1.570 kb)<br>primers: T*niaD*-T*gluc*-F/P*oliC*-pRS426-3R<br>template: pNDG-OGG	n/a	YRC
**pP-GGG**<br>(8.997 kb) [pEC0191]	**pRS426**$^{\Delta NcoI}$/<br>*Eco*RI + *Xho*I	**[(P*gpdA::gfp::*T*gluc*)]** (2.182 kb)<br>primers: P*gpdA*-pRS426-5F/T*gluc*-R1<br>template: pNAH-GGG	**[(P*trpC::bar::*T*niaD*)]** (1.327 kb)<br>primers: T*niaD*-T*gluc*-F/P*oliC*-pRS426-3R<br>template: pNDP-OGG	n/a	YRC
**pS-GGG**<br>(10.932 kb) [pEC0192]	**pRS426**$^{\Delta NcoI}$/<br>*Eco*RI + *Xho*I	**[(P*gpdA::gfp::*T*gluc*)]** (2.182 kb)<br>primers: P*gpdA*-pRS426-5F/T*gluc*-R1<br>template: pNAH-GGG	**[(P*trpC::sur::*T*niaD*)]** (3.262 kb)<br>primers: T*niaD*-T*gluc*-F/P*oliC*-pRS426-3R<br>template: pNDS-OGG	n/a	YRC
**pAMA/ribo-*kppks1*** ^{PS4}<br>(16.506 kb) [pEC0115]	**pFC332**/<br>*Pac*I	***kppks1*** ^{PS4} **ribo-A** (0.570 kb)<br>primers: pFC334-F1/*kppks1*-AMA-PS4R<br>template: pFC334	***kppks1*** ^{PS4} **ribo-B** (0.463 kb)<br>primers: *kppks1*-AMA-PS4F/pFC334-R1<br>template: pFC334	n/a	HiFi
**pNAH-QGT**<br>(11.161 kb) [pEC0104]	**pNAH-AGT**/ *Nco*I<br>+ *Spe*I	**P*ffgln1*** (0.889 kb)<br>primers: P*ffgln1*-P*trpC*-F/P*ffgln1*-*gfp*-R<br>template: *F. fujikuroi* DNA	n/a	n/a	YRC
**pNAH_**^{G}***kpwcl1-gfp***^{G}<br>(14.281 kb) [pEC0043]	**pNAH-OGG**/ *Nco*I<br>+ *Spe*I	**P*gpdA*** (0.840 kb)<br>primers: P*gpdA*-P*trpC*-F/P*gpdA*-ATG-R<br>template: pHR-*gfp*-natR	***kpwcl1*** (3.266 kb)<br>primers: *kpwcl1*-P*gpdA*-F/*kpwcl1*-*gfp*-R<br>template: *K. petricola* DNA	n/a	YRC
**pNAH_**^{G}***kpwcl1-mch***^{T}<br>(14.478 kb) [pEC0044]	**pNAH-OCT**/ *Nco*I<br>+ *Spe*I	**P*gpdA*** (0.840 kb)<br>primers: P*gpdA*-P*trpC*-F/P*gpdA*-ATG-R<br>template: pHR-*gfp*-natR	***kpwcl1*** (3.266 kb)<br>primers: *kpwcl1*-P*gpdA*-F/*kpwcl1*-*gfp*-R<br>template: *K. petricola* DNA	n/a	YRC
**pNAH_**^{G}***kpwcl1-gfpC***^{G}<br>(13.809 kb) [pEC0045]	**pNAH-OGCGc**/<br>*Nco*I + *Spe*I	**P*gpdA*** (0.840 kb)<br>primers: P*gpdA*-P*trpC*-F/P*gpdA*-ATG-R<br>template: pHR-*gfp*-natR	***kpwcl1*** (3.266 kb)<br>primers: *kpwcl1*-P*gpdA*-F/-OGCGc-R<br>template: *K. petricola* DNA	n/a	YRC
**pNDN_**^{O}***gfp-kpwcl2***^{G}<br>(12.168 kb) [pEC0129]	**pNDN-OGG**/<br>*Not*I	***kpwcl2*** (1.554 kb)<br>primers: *kpwcl2*-*gfp*-F/*kpwcl2*-T*gluc*-R<br>template: *K. petricola* DNA	n/a	n/a	YRC
**pNDN_**^{A}***gfpN-kpwcl2***^{T}<br>(12.090 kb) [pEC0038]	**pNDN-AGNTn**/<br>*Not*I	***kpwcl2*** (1.544 kb)<br>primers: *kpwcl2*-AGNTn-F/*kpwcl2*-T*trpC*-R<br>template: *K. petricola* DNA	n/a	n/a	YRC
**pNDN_**^{G}***gfpN-kpwcl2*T**<br>(12.111 kb) [pEC0130]	**pNDN_**^{A}***gfpN-kpwcl2***^{T}/ *Spe*I	**P*gpdA*** (0.840 kb)<br>primers: P*gpdA*- *Spe*I-P*trpC*-F/P*gpdA*-ATG-R<br>template: pHR-*gfp*-natR	n/a	n/a	YRC
**pNDN-GGNTn**<br>(10.569 kb) [pEC0131]	**pNDN-AGNTn**/<br>*Nco*I + *Spe*I	**P*gpdA*** (0.840 kb)<br>primers: P*gpdA*-*Spe*I-P*trpC*-F/P*gpdA*-ATG-R<br>template: pHR-*gfp*-natR	n/a	n/a	YRC
**pNDN-GGNTc**<br>(10.548 kb) [pEC0200]	**pNDN-AGNTc**/<br>*Nco*I + *Spe*I	**P*gpdA*** (0.927 kb)<br>primers: P*gpdA*-*Spe*I-P*trpC*-F/-11aalinker-R<br>template: pHR-*gfp*-natR	n/a	n/a	YRC

**Plasmid name** (size) [ID]	**Entry plasmid**	**Amplicon 1**	**Amplicon 2**	**Amplicon 3**	**Method**
**pIGR1H-AGT**<br>(11.704 kb) [pEC0077]	**pRS426$^{\Delta NcoI}$**/<br>*Eco*RI + *Xho*I	**[(hygR)–(P*act1*::*gfp*::T*trpC*)]** (4.039 kb)<br>primers: T*niaD*-5FAMR/T*trpC*-3RAMR<br>template: pNDH-AGT	***kpigr1* 5' flank** (1.185 kb)<br>primers: *kpigr1*-5F/*kpigr1*-5R<br>template: *K. petricola* DNA	***kpigr1* 3' flank** (0.848 kb)<br>primers: *kpigr1*-3F/*kpigr1*-3R<br>template: *K. petricola* DNA	YRC
**pIGR2H-AGT**<br>(12.179 kb) [pEC0064]	**pRS426$^{\Delta NcoI}$**/<br>*Eco*RI + *Xho*I	**[(hygR)–(P*act1*::*gfp*::T*trpC*)]** (4.039 kb)<br>primers: T*niaD*-5FAMR/T*trpC*-3RAMR<br>template: pNDH-AGT	***kpigr2* 5' flank** (0.917 kb)<br>primers: *kpigr2*-5F/*kpigr2*-5R<br>template: *K. petricola* DNA	***kpigr2* 3' flank** (1.592 kb)<br>primers: *kpigr2*-3F/*igr2*-3R<br>template: *K. petricola* DNA	YRC
**pIGR2H-LGT**<br>(11.986 kb) [pEC0067]	**pIGR2H-AGT**/<br>*Spe*I + *Nco*I	**P*kpgal1*** (0.710 kb)<br>primers: P*kpgal1*-P*trpC*-F1/P*kpgal1*-*ogfp*-R1<br>template: *K. petricola* DNA	n/a	n/a	YRC
**pIGR2H-NGT**<br>(12.588 kb) [pEC0068]	**pIGR2H-AGT**/<br>*Spe*I + *Nco*I	**P*anniaD*** (1.318 kb)<br>primers: P*anniaD*-P*trpC*-F1/-*ogfp*-R1<br>template: *A. nidulans* DNA	n/a	n/a	YRC
**pIGR1N-OCT**<br>(11.526 kb) [pEC0089]	**pRS426$^{\Delta NcoI}$**/<br>*Eco*RI + *Xho*I	**[(natR)–(P*oliC*::*mch*::T*tub1*)]** (3.859 kb)<br>primers: T*niiA*-5FAMR/T*tub1*-3RAMR<br>template: pNAN-OCT	***kpigr1* 5' flank** (1.185 kb)<br>primers: *kpigr1*-5F/*kpigr1*-5R<br>template: *K. petricola* DNA	***kpigr1* 3' flank** (0.848 kb)<br>primers: *kpigr1*-3F/*kpigr1*-3R<br>template: *K. petricola* DNA	YRC
**pIGR2N-OCT**<br>(12.001 kb) [pEC0090]	**pRS426$^{\Delta NcoI}$**/<br>*Eco*RI + *Xho*I	**[(natR)–(P*oliC*::*mch*::T*tub1*)]** (3.859 kb)<br>primers: T*niiA*-5FAMR/T*tub1*-3RAMR<br>template: pNAN-OCT	***kpigr2* 5' flank** (0.917 kb)<br>primers: *kpigr2*-5F/*kpigr2*-5R<br>template: *K. petricola* DNA	***kpigr2* 3' flank** (1.592 kb)<br>primers: *kpigr2*-3F/*kpigr2*-3R<br>template: *K. petricola* DNA	YRC
**pAMA/ribo-*kpigr1* ^{PS1}**<br>(16.506 kb) [pEC0058]	**pFC332**/<br>*Pac*I	***kpigr1* ^{PS1} ribo-A** (0.570 kb)<br>primers: pFC334-F1/*kpigr1*-AMA-PSR<br>template: pFC334	***kpigr1* ^{PS1} ribo-B** (0.463 kb)<br>primers: *kpigr1*-AMA-PSF/pFC334-R1<br>template: pFC334	n/a	HiFi
**pAMA/ribo-*kpigr2* ^{PS1}**<br>(16.506 kb) [pEC0059]	**pFC332**/<br>*Pac*I	***kpigr2* ^{PS1} ribo-A** (0.570 kb)<br>primers: pFC334-F1/*kpigr2*-AMA-PSR<br>template: pFC334	***kpigr2* ^{PS1} ribo-B** (0.463 kb)<br>primers: *kpigr2*-AMA-PSF/pFC334-R1<br>template: pFC334	n/a	HiFi
**pAMA/tRNA-*kpigr1* ^{PS1} -*kpigr2* ^{PS1}**<br>(16.670 kb) [pEC0194]	**pFC332**/<br>*Pac*I	***kpigr1* ^{PS1} tRNA-A** (0.589 kb)<br>primers: pFC334-F1/*kpigr1*-tRNA-PS1R<br>template: pFC902	***kpigr1* ^{PS1}/*kpigr2* ^{PS1} tRNA-C** (0.191 kb)<br>primers: *kpigr1*-tRNA-PS1F/*igr2*-tRNA-PS1R<br>template: pFC902	***kpigr2* ^{PS2} tRNA-B** (0.427 kb)<br>primers: *kpigr2*-tRNA-PS1F/pFC334-R1<br>template: pFC902	HiFi
**pIGR1H**<br>(9.444 kb) [pEC0087]	**pRS426$^{\Delta NcoI}$**/<br>*Eco*RI + *Xho*I	**[(P*trpC*::*hph*::T*niaD*)]** (1.777 kb)<br>primers: P*trpC*-3RAMR2/T*niaD*-5FAMR<br>template: pNDH-AGT	***kpigr1* 5' flank** (1.185 kb)<br>primers: *kpigr1*-5F/*kpigr1*-5R<br>template: *K. petricola* DNA	***kpigr1* 3' flank** (0.848 kb)<br>primers: *kpigr1*-3F/*kpigr1*-3R<br>template: *K. petricola* DNA	YRC
**pIGR2H**<br>(9.919 kb) [pEC0088]	**pRS426$^{\Delta NcoI}$**/<br>*Eco*RI + *Xho*I	**[(P*trpC*::*hph*::T*niaD*)]** (1.777 kb)<br>primers: P*trpC*-3RAMR2/T*niaD*-5FAMR<br>template: pNDH-AGT	***kpigr2* 5' flank** (0.917 kb)<br>primers: *kpigr2*-5F/*kpigr2*-5R<br>template: *K. petricola* DNA	***kpigr2* 3' flank** (1.592 kb)<br>primers: *kpigr2*-3F/*kpigr2*-3R<br>template: *K. petricola* DNA	YRC
**pIGR1N**<br>(8.976 kb) [pEC0208]	**pIGR1H**/<br>*Pac*I + *Asc*I	**[(P*trpC*::*nat1*::T*niaD*)]** (1.385 kb)<br>primers: P*trpC*-3RAMR2/T*niaD*-5FAMR<br>template: pNDN-OGG	n/a	n/a	YRC
**pIGR2N**<br>(9.451 kb) [pEC0209]	**pIGR2H**/<br>*Pac*I + *Asc*I	**[(P*trpC*::*nat1*::T*niaD*)]** (1.385 kb)<br>primers: P*trpC*-3RAMR2/T*niaD*-5FAMR<br>template: pNDN-OGG	n/a	n/a	YRC

Plasmid name (size) [ID]	Entry plasmid	Amplicon 1	Amplicon 2	Amplicon 3	Method
**pIGR1G** (9.201 kb) [pEC0175]	**pIGR1H**/ *Pac*I + *Asc*I	**[(P*trpC*::*nptII*::T*niaD*)]** (1.610 kb) primers: P*trpC*-3RAMR2/T*niaD*-5FAMR template: pNDG-OGG	n/a	n/a	YRC
**pIGR2G** (9.676 kb) [pEC0176]	**pIGR2H**/ *Pac*I + *Asc*I	**[(P*trpC*::*nptII*::T*niaD*)]** (1.610 kb) primers: P*trpC*-3RAMR2/T*niaD*-5FAMR template: pNDG-OGG	n/a	n/a	YRC
**pIGR1P** (8.958 kb) [pEC0177]	**pIGR1H**/ *Pac*I + *Asc*I	**[(P*trpC*::*bar*::T*niaD*)]** (1.367 kb) primers: P*trpC*-3RAMR2/T*niaD*-5FAMR template: pNDP-OGG	n/a	n/a	YRC
**pIGR2P** (9.433 kb) [pEC0178]	**pIGR2H**/ *Pac*I + *Asc*I	**[(P*trpC*::*bar*::T*niaD*)]** (1.367 kb) primers: P*trpC*-3RAMR2/T*niaD*-5FAMR template: pNDP-OGG	n/a	n/a	YRC
**pIGR1S** (10.893 kb) [pEC0179]	**pIGR1H**/ *Pac*I + *Asc*I	**[(P*trpC*::*sur*::T*niaD*)]** (3.302 kb) primers: P*trpC*-3RAMR2/T*niaD*-5FAMR template: pNDS-OGG	n/a	n/a	YRC
**pIGR2S** (11.368 kb) pEC0180]	**pIGR2H**/ *Pac*I + *Asc*I	**[(P*trpC*::*sur*::T*niaD*)]** (3.302 kb) primers: P*trpC*-3RAMR2/T*niaD*-5FAMR template: pNDS-OGG	n/a	n/a	YRC
**pAMA/ribo-*kpura3*^{PS3}** (16.506 kb) [pEC0099]	**pFC332**/ *Pac*I	***kpura3*^{PS3} ribo-A** (0.570 kb) primers: pFC334-F1/*kpura3*-AMA-PS3R template: pFC334	***kpura3*^{PS3} ribo-B** (0.463 kb) primers: *kpura3*-AMA-PS3F/pFC334-R1 template: pFC334	n/a	HiFi

Plasmids were generated via yeast recombination cloning (YRC) or NEBuilder HiFi DNA assembly (HiFi).

Plasmids used as entry plasmids in assembly or as templates for amplification of DNA fragments were cloned in this study (Supplementary Table 3) or were published previously: pFC332, pFC334 (Nødvig et al., 2015); pFC902 (Nødvig et al., 2018); pRS426$^{\Delta NcoI}$, pNAN-OCT, pNAH-OGG, pNAN-OGG, pNAH-OCT, pNDH-AGT, pNDN-AGT, pNAH-AGT pNAH-OGG, pNAH-OGCGc, pNDN-AGNTn, pNDN-AGNTc, pNDH-OGG, pNDN-OGG (Schumacher, 2012); pHR-*gfp*-natR (Voigt et al., 2020); pKS-Gen [*nptII*] (Bluhm et al., 2008); pCB1524 [*bar*], pCB1532 [*sur*] (Sweigard et al., 1997; McCluskey et al., 2010).

Supplementary Table 4. Transformations of *K. petricola* protoplasts carried out in this study.

Strain name	GMO ID	Entry	Donor DNA with SH/LH flanks	Donor DNA with SH/LH flanks	CRISPR/Cas9 plasmid
pks1–<br>**(1A)**	KP-0199	WT:A95	n/a	n/a	**pAMA/tRNA-*kppks1*** PS2<br>(circular)
pks1–<br>**(1B)**	KP-0200	WT:A95	***pks1*-oligo-2.7** (0.080 kb)<br>(single-stranded oligonucleotide with 3-bp-long deletion)	n/a	**pAMA/tRNA-*kppks1*** PS2<br>(circular)
**Δ*pks1* [N]**<br>**(1C)**	KP-0181	WT:A95	**[(T*niaD*::*nat1*::P*trpC*)]** Δ*pks1* (1.473 kb)<br>primers: *kppks1*-RT5F/-RT3R<br>template: pNDN-OGG	n/a	**pAMA/tRNA-*kppks1*** PS2<br>(circular)
**Δ*pks1* [N]**<br>**(1D)** negative control	KP-0070	WT:A95	**[(T*niaD*::*nat1*::P*trpC*)]** Δ*pks1* (1.473 kb)<br>primers: *kppks1*-RT5F/-RT3R<br>template: pNDN-OGG	n/a	n/a
**Δ*pks1* [N]**<br>**(RC1)** positive control	KP-0072	WT:A95	**[(T*niaD*::*nat1*::P*trpC*)]** Δ*pks1* (1.473 kb)<br>primers: *kppks1*-RT5F/-RT3R<br>template: pNDN-OGG	n/a	**pAMA/ribo-*kppks1*** PS2<br>(circular)
pks1–/phs1–<br>**(2A)**	KP-0201	*pks1–*	n/a	n/a	**pAMA/tRNA-*kpphs1*** PS1<br>(circular)
pks1–/phs1–<br>**(2B)**	KP-0202	*pks1–*	***kpphs1*-oligo-1** (0.080 kb)<br>(single-stranded oligonucleotide with inserted stop codon)	n/a	**pAMA/tRNA-*kpphs1*** PS1<br>(circular)
***pks1–*/Δ*phs1* [H]**<br>**(2C)**	KP-0188	*pks1–*	**[(T*niaD*::*hph*::P*trpC*)]** Δ*phs1* (1.946 kb)<br>primers: *kpphs1*-RT5F/-RT3R<br>template: pNDH-OGG	n/a	**pAMA/tRNA-*kpphs1*** PS1<br>(circular)
***pks1–*/Δ*phs1* [H]**<br>**(2D)** negative control	KP-0203	*pks1–*	**[(T*niaD*::*hph*::P*trpC*)]** Δ*phs1* (1.946 kb)<br>primers: *kpphs1*-RT5F/-RT3R<br>template: pNDH-OGG	n/a	n/a
***pks1–*/Δ*phs1* [H]**<br>**(RC2)** positive control	KP-0187	*pks1–*	**[(T*niaD*::*hph*::P*trpC*)]** Δ*phs1* (1.946 kb)<br>primers: *kpphs1*-RT5F/-RT3R<br>template: pNDH-OGG	n/a	**pAMA/ribo-*kpphs1*** PS1<br>(circular)
pks1–/phs1–<br>**(3A)**	KP-0204	WT:A95	n/a	n/a	**pAMA/tRNA-*kppks1*** PS2-<br>***kpphs1*** PS1 (circular)

Strain name	GMO ID	Entry	Donor DNA with SH/LH flanks	Donor DNA with SH/LH flanks	CRISPR/Cas9 plasmid
pks1–/phs1–<br>**(3B)**	KP-0205	WT:A95	***kppks1*-oligo-2.7** (0.080 kb)<br>(single-stranded oligonucleotide with<br>3-bp-long deletion)	***kpphs1*-oligo-1** (0.080 kb)<br>(single-stranded oligonucleotide with<br>inserted stop codon)	**pAMA/tRNA-*kppks1*** ^{PS2}**-*kpphs1*** ^{PS1} (circular)
**Δ*pks1*/Δ*phs1* [N/H]**<br>**(3C)**	KP-0193	WT:A95	**[(T*niaD*::*nat1*::P*trpC*)]** $^{\Delta pks1}$ (1.473 kb)<br>primers: *kppks1*-RT5F/-RT3R<br>template: pNDN-OGG	**[(T*niaD*::*hph*::P*trpC*)]** $^{\Delta phs1}$ (1.946 kb)<br>primers: *kpphs1*-RT5F/-RT3R<br>template: pNDH-OGG	**pAMA/tRNA-*kppks1*** ^{PS2}**-*kpphs1*** ^{PS1} (circular)
**Δ*pks1*/Δ*phs1* [N/H]**<br>**(3D)** negative control	KP-0082	WT:A95	**[(T*niaD*::*nat1*::P*trpC*)]** $^{\Delta pks1}$ (1.473 kb)<br>primers: *kppks1*-RT5F/-RT3R<br>template: pNDN-OGG	**[(T*niaD*::*hph*::P*trpC*)]** $^{\Delta phs1}$ (1.946 kb)<br>primers: *kpphs1*-RT5F/-RT3R<br>template: pNDH-OGG	n/a
**Δ*pks1*/Δ*phs1* [N/H]**<br>**(RC3)** positive control	KP-0083	WT:A95	**[(T*niaD*::*nat1*::P*trpC*)]** $^{\Delta pks1}$ (1.473 kb)<br>primers: *kppks1*-RT5F/-RT3R<br>template: pNDN-OGG	**[(T*niaD*::*hph*::P*trpC*)]** $^{\Delta phs1}$ (1.946 kb)<br>primers: *kpphs1*-RT5F/-RT3R<br>template: pNDH-OGG	**pAMA/ribo-*kppks1*** ^{PS2}<br>**pAMA/ribo-*kpphs1*** ^{PS1}<br>(circular)
**Δ*pks1* [H-AGT]**	KP-0115	WT:A95	**[(hygR)–(P*act1*::*gfp*::T*trpC*)]** $^{\Delta pks1}$ (4.183 kb)<br>primers: T*niaD*-*pks1*-SH5F/T*trpC*-*pks1*-SH3R<br>template: pIGR2H-AGT	n/a	**pAMA/ribo-*kppks1*** ^{PS2}<br>(circular)
**Δ*pks1* [H-GGG]**	KP-0116	WT:A95	**[(hygR)–(P*gpdA*::*gfp*::T*gluc*)]** $^{\Delta pks1}$ (4.316 kb)<br>primers: T*niiA*-*pks1*-SH5F/T*gluc*-*pks1*-SH3R<br>template: pNAH-GGG	n/a	**pAMA/ribo-*kppks1*** ^{PS2}<br>(circular)
**Δ*pks1* [H-OGG]**	KP-0117	WT:A95	**[(hygR)–(P*oliC*::*gfp*::T*gluc*)]** $^{\Delta pks1}$ (4.053 kb)<br>primers: T*niaD*-*pks1*-SH5F/T*gluc*-*pks1*-SH3R<br>template: pNDH-OGG	n/a	**pAMA/ribo-*kppks1*** ^{PS2}<br>(circular)
**Δ*pks1* [H-QGT]**	KP-0119	WT:A95	**[(hygR)–(P*gln1*::*gfp*::T*trpC*)]** $^{\Delta pks1}$ (4.456 kb)<br>primers: T*niiA*-*pks1*-SH5F/T*trpC*-*pks1*-SH3R<br>template: pNAH-QGT	n/a	**pAMA/ribo-*kppks1*** ^{PS2}<br>(circular)
**Δ*pks1* [H-LGT]**	KP-0120	WT:A95	**[(hygR)–(P*gal1*::*gfp*::T*trpC*)]** $^{\Delta pks1}$ (3.984 kb)<br>primers: T*niaD*-*pks1*-SH5F/T*trpC*-*pks1*-SH3R<br>template: pIGR2H-LGT	n/a	**pAMA/ribo-*kppks1*** ^{PS2}<br>(circular)
**Δ*pks1* [H-NGT]**	KP-0121	WT:A95	**[(hygR)–(P*niaD*::*gfp*::T*trpC*)]** $^{\Delta pks1}$ (4.592 kb)<br>primers: T*niaD*-*pks1*-SH5F/T*trpC*-*pks1*-SH3R<br>template: pIGR2H-NGT	n/a	**pAMA/ribo-*kppks1*** ^{PS2}<br>(circular)
***phs1–/Pact1::pks1* [H]**	KP-0141	*phs1–*	**[(hygR)–(P*act1*)]** $^{\Delta Ppks1}$ (2.769 kb)<br>primers: T*niaD*-*pks1*-SH5F/*pks1*-P*act1*-SH3R<br>template: pIGR2H-AGT	n/a	**pAMA/ribo-*kppks1*** ^{PS4}<br>(circular)
***phs1–/PgpdA::pks1* [H]**	KP-0142	*phs1–*	**[(hygR)–(P*gpdA*)]** $^{\Delta Ppks1}$ (3.035 kb)<br>primers: T*niiA*-*pks1*-SH5F/*pks1*-P*gpdA*-SH3R<br>template: pNAH-GGG	n/a	**pAMA/ribo-*kppks1*** ^{PS4}<br>(circular)

Strain name	GMO ID	Entry	Donor DNA with SH/LH flanks	Donor DNA with SH/LH flanks	CRISPR/Cas9 plasmid
***phs1–*/P*oliC*::*pks1* [H]**	KP-0143	*phs1–*	**[(hygR)–(P*oliC*)]** $^{\Delta Ppks1}$ (2.772 kb)<br>primers: T*niaD*-*pks1*-SH5F/*pks1*-P*oliC*-SH3R<br>template: pNDH-OGG	n/a	**pAMA/ribo-*kppks1*** ^{PS4}<br>(circular)
***phs1–*/P*gln1*::*pks1* [H]**	KP-0144	*phs1–*	**[(hygR)–(P*gln1*)]** $^{\Delta Ppks1}$ (3.052 kb)<br>primers: T*niiA*-*pks1*-SH5F/*pks1*-P*gln1*-SH3R<br>template: pNAH-QGT	n/a	**pAMA/ribo-*kppks1*** ^{PS4}<br>(circular)
***phs1–*/P*gal1*::*pks1* [H]**	KP-0145	*phs1–*	**[(hygR)–(P*gal1*)]** $^{\Delta Ppks1}$ (2.570 kb)<br>primers: T*niaD*-*pks1*-SH5F/*pks1*-P*gal1*-SH3R<br>template: pIGR2H-LGT	n/a	**pAMA/ribo-*kppks1*** ^{PS4}<br>(circular)
***phs1–*/P*niaD*::*pks1* [H]**	KP-0146	*phs1–*	**[(hygR)– (P*niaD*)]** $^{\Delta Ppks1}$ (3.183 kb)<br>primers: T*niaD*-*pks1*-SH5F/*pks1*-P*niaD*-SH3R<br>template: pIGR2H-NGT	n/a	**pAMA/ribo-*kppks1*** ^{PS4}<br>(circular)
***phs1–*/ΔP*pks1* [H]**	KP-0147	*phs1–*	**[(T*niaD*::*nat1*::P*trpC*)]** $^{\Delta Ppks1}$ (1.912 kb)<br>primers: T*niaD*-*pks1*-SH5F/*pks1*-P*trpC*-SH3R<br>template: pIGR2H-AGT	n/a	**pAMA/ribo-*kppks1*** ^{PS4}<br>(circular)
**P*gal1*::*pks1* [H]**	KP-0265	WT:A95	**[(hygR)–(P*gal1*)]** $^{\Delta Ppks1}$ (2.570 kb)<br>primers: T*niaD*-*pks1*-SH5F/*pks1*-P*gal1*-SH3R<br>template: pIGR2H-LGT	n/a	**pAMA/ribo-*kppks1*** ^{PS4}<br>(circular)
**ΔP*pks1*::*pks1* [H]**	KP-0260	WT:A95	**[(T*niaD*::*nat1*::P*trpC*)]** $^{\Delta Ppks1}$ (1.912 kb)<br>primers: T*niaD*-*pks1*-SH5F/*pks1*-P*trpC*-SH3R<br>template: pIGR2H-AGT	n/a	**pAMA/ribo-*kppks1*** ^{PS4}<br>(circular)
***igr1* [H-AGT]**	KP-0106	WT:A95	**[(hygR)–(P*act1*::*gfp*::T*trpC*)]** ^{igr1} (6.188 kb)<br>f isolated by digestion with *Apa*I<br>from pIGR1H-AGT	n/a	**pAMA/ribo-*kpigr1*** ^{PS1}<br>(circular)
***igr1* [N-OCT]**	KP-0101	WT:A95	**[(natR)–(P*oliC*::*mch*::T*tub1*)]** ^{igr1} (6.004 kb)<br>isolated by digestion with *Swa*I<br>from pIGR1N-OCT	n/a	**pAMA/ribo-*kpigr1*** ^{PS1}<br>(circular)
***igr1* [H-GGG]**	KP-0172	WT:A95	**[(hygR)–(P*gpdA*::*gfp*::T*gluc*)]** ^{igr1} (4.309 kb)<br>primers: T*niiA*-*igr1*-SH5F/T*gluc*-*igr1*-SH3R<br>template: pNAH-GGG	n/a	**pAMA/ribo-*kpigr1*** ^{PS1}<br>(circular)
***igr1* [H-OGG]**	KP-0173	WT:A95	**[(hygR)–(P*oliC*::*gfp*::T*gluc*)]** ^{igr1} (4.055 kb)<br>primers: T*niaD*-*igr1*-SH5F/T*gluc*-*igr1*-SH3R<br>template: pNDH-OGG	n/a	**pAMA/ribo-*kpigr1*** ^{PS1}<br>(circular)
***igr2* [H-AGT]**	KP-0094	WT:A95	**[(hygR)–(P*act1*::*gfp*::T*trpC*)]** ^{igr2} (6.637 kb)<br>isolated by digestion with *Swa*I<br>from pIGR2H-AGT	n/a	**pAMA/ribo-*kpigr2*** ^{PS1}<br>(circular)

Strain name	GMO ID	Entry	Donor DNA with SH/LH flanks	Donor DNA with SH/LH flanks	CRISPR/Cas9 plasmid
***igr2* [N-OCT]**	KP-0108	WT:A95	**[(natR)–(P*oliC*::*mch*::T*tub1*)]** ^{igr2} (6.489 kb) isolated by digestion with *Sac*II from pIGR2N-OCT	n/a	**pAMA/ribo-*kpigr2*** ^{PS1} (circular)
***igr2* [H-GGG]**	KP-0176	WT:A95	**[(hygR)–(P*gpdA*::*gfp*::T*gluc*)]** ^{igr2} (4.310 kb) primers: T*niiA*-*igr2*-SH5F/ T*gluc*-*igr2*-SH3R template: pNAH-GGG	n/a	**pAMA/ribo-*kpigr2*** ^{PS1} (circular)
***igr2* [H-OGG]**	KP-0177	WT:A95	**[(hygR)–(P*oliC*::*gfp*::T*gluc*)]** ^{igr2} (4.055 kb) primer: T*niaD*-*igr2*-SH5F/ T*gluc*-*igr2*-SH3R template: pNDH-OGG	n/a	**pAMA/ribo-*kpigr2*** ^{PS1} (circular)
**Δ*ura3* [H-AGT]**	KP-0148	WT:A95	**[(hygR)–(P*act1*::*gfp*::T*trpC*)]** $^{\Delta ura3}$ (4.185 kb) primer: T*niaD*-*ura3*-SH5F/T*trpC*-*ura3*-SH3R template: pIGR2H-AGT	n/a	**pAMA/ribo-*kpura3*** ^{PS3} (circular)
**Δ*ura3* [H-OGG]**	KP-0149	WT:A95	**[(hygR)–(P*oliC*::*gfp*::T*gluc*)]** $^{\Delta ura3}$ (4.055 kb) primer: T*niaD*-*ura3*-SH5F/T*gluc*-*ura3*-SH3R template: pNDH-OGG	n/a	**pAMA/ribo-*kpura3*** ^{PS3} (circular)
**Δ*ura3* [H-GGG]**	KP-0171	WT:A95	**[(hygR)–(P*gpdA*::*gfp*::T*gluc*)]** $^{\Delta ura3}$ (4.318 kb) primer: T*niiA*-*ura3*-SH5F/T*gluc*-*ura3*-SH3R template: pNAH-GGG	n/a	**pAMA/ribo-*kpura3*** ^{PS3} (circular)
**Δ*pks1* [G]**	KP-0192	WT:A95	**[(T*niaD*::*nptII*::P*trpC*)]** $^{\Delta pks1}$ (1.698 kb) primers: *kppks1*-RT5F/-RT3R template: pNDG-OGG	n/a	**pAMA/ribo-*kppks1*** ^{PS2} (circular)
**Δ*pks1* [P]**	KP-0242	WT:A95	**[(T*niaD*::*bar*::P*trpC*)]** $^{\Delta pks1}$ (1.455 kb) primers: *kppks1*-RT5F/-RT3R template: pNDP-OGG	n/a	**pAMA/ribo-*kppks1*** ^{PS2} (circular)
**Δ*pks1* [S]**	KP-0243	WT:A95	**[(T*niaD*::*sur*::P*trpC*)]** $^{\Delta pks1}$ (3.390 kb) primers: *kppks1*-RT5F/-RT3R template: pNDS-OGG	n/a	**pAMA/ribo-*kppks1*** ^{PS2} (circular)
***igr2* [G-OGG]**	KP-0246	WT:A95	**[(genR)–[P*oliC*::*gfp*::T*gluc*)]** ^{igr2} (3.816 kb) primers: T*niaD*-*igr2*-SH5F/T*gluc*-*igr2*-SH3R template: pNDG-OGG	n/a	**pAMA/ribo-*kpigr2*** ^{PS1} (circular)
***igr2* [P-OGG]**	KP-0247	WT:A95	**[(baR)–[P*oliC*::*gfp*::T*gluc*)]** ^{igr2} (3.578 kb) primers: T*niaD*-*igr2*-SH5F/T*gluc*-*igr2*-SH3R template: pNDP-OGG	n/a	**pAMA/ribo-*kpigr2*** ^{PS1} (circular)
***igr2* [S-OGG]**	KP-0248	WT:A95	**[(suR)–(P*oliC*::*gfp*::T*gluc*)]** ^{igr2} (5.508 kb) primers: T*niaD*-*igr2*-SH5F/T*gluc*-*igr2*-SH3R template: pNDS-OGG	n/a	**pAMA/ribo-*kpigr2*** ^{PS1} (circular)

Strain name	GMO ID	Entry	Donor DNA with SH/LH flanks	Donor DNA with SH/LH flanks	CRISPR/Cas9 plasmid
**Δ*pks1* [H-W1G]**	KP-0182	WT:A95	**[(hygR)–(P*gpdA*::*wcl1-gfp*::T*gluc*)]** $^{\Delta pks1}$ (7.423 kb)<br>primers: T*niiA*-*pks1*-SH5F/T*gluc*-*pks1*-SH3R<br>template: pNAH_^{G}*kpwcl1-gfp*^{G}	n/a	**pAMA/ribo-*kppks1*** ^{PS2}<br>(circular)
**Δ*pks1* [N-GW2]**	KP-0186	WT:A95	**[(natR)–(P*oliC*::*gfp-wcl2*::T*gluc*)]** $^{\Delta pks1}$ (5.127 kb)<br>primers: T*niaD*-*pks1*-SH5F/T*gluc*-*pks1*-SH3R<br>template: pNDN_^{O}*gfp-kpwcl2*^{G}	n/a	**pAMA/ribo-*kppks1*** ^{PS2}<br>(circular)
**Δ*pks1* [H-W1M] +<br>Δ*phs1* [N-GW2]** (B1)	KP-0214	WT:A95	**[(hygR)–(P*gpdA*::*wcl1-mch*::T*tub1*)]** $^{\Delta pks1}$ (6.949 kb)<br>primers: T*niiA*-*pks1*-SH5F/T*tub1*-*pks1*-SH3R<br>template: pNAH_^{G}*kpwcl1-mch*^{T}	**[(natR)–(P*oliC*::*gfp-wcl2*::T*gluc*)]** $^{\Delta phs1}$ (5.127 kb)<br>primers: T*niaD*-*pks1*-SH5F/T*gluc*-*pks1*-SH3R<br>template: pNDN_^{O}*gfp-kpwcl2*^{G}	**pAMA/tRNA-*kppks1*** ^{PS2}**-<br>*kpphs1*** ^{PS1} (circular)
**Δ*pks1* [H-W1GC] +<br>Δ*phs1* [N-GNW2]** (B2)	KP-0215	WT:A95	**[(hygR)–(P*gpdA*::*wcl1-gfpC*::T*gluc*)]** $^{\Delta pks1}$ (7.620 kb)<br>primers: T*niiA*-*pks1*-SH5F/T*gluc*-*pks1*-SH3R<br>template: pNAH_^{G}*kpwcl1-gfpC*^{G}	**[(natR)–(P*gpdA*::*gfpN-wcl2*::T*trpC*)]** $^{\Delta phs1}$ (5.071 kb)<br>primers: T*niaD*-*phs1*-SH5F/T*gluc*-*phs1*-SH3R<br>template: pNDN_^{G}*gfpN-kpwcl2*^{T}	**pAMA/tRNA-*kppks1*** ^{PS2}**-<br>*kpphs1*** ^{PS1} (circular)
**Δ*pks1* [H-W1GC] +<br>Δ*phs1* [N-GN]** (B3)	KP-0216	WT:A95	**[(hygR)–(P*gpdA*::*wcl1-gfpC*::T*gluc*)]** $^{\Delta pks1}$ (7.620 kb)<br>primers: T*niiA*-*pks1*-SH5F/T*gluc*-*pks1*-SH3R<br>template: pNAH_^{G}*kpwcl1-gfpC*^{G}	**[(natR)–(P*gpdA*::*gfpN*::T*trpC*)]** $^{\Delta phs1}$ (3.529 kb)<br>primers: T*niaD*-*phs1*-SH5F/T*trpC*-*phs1*-SH3R<br>template: pNDN-GGNTn	**pAMA/tRNA-*kppks1*** ^{PS2}**-<br>*kpphs1*** ^{PS1} (circular)
**Δ*pks1* [H-GC] +<br>Δ*phs1* [N-GNW2]** (B4)	KP-0217	WT:A95	**[(hygR)–(P*oliC*::*gfpC*::T*gluc*)]** $^{\Delta pks1}$ (3.678 kb)<br>primers: T*niiA*-*pks1*-SH5F/T*gluc*-*pks1*-SH3R<br>template: pNAH-OGCGc	**[(natR)–(P*gpdA*::*gfpN-wcl2*::T*trpC*)]** $^{\Delta phs1}$ (5.071 kb)<br>primers: T*niaD*-*phs1*-SH5F/T*gluc*-*phs1*-SH3R<br>template: pNDN_^{G}*gfpN-kpwcl2*^{T}	**pAMA/tRNA-*kppks1*** ^{PS2}**-<br>*kpphs1*** ^{PS1} (circular)
**Δ*pks1* [H-GC] +<br>Δ*phs1* [N-GN]** (B5)	KP-0218	WT:A95	**[(hygR)–(P*oliC*::*gfpC*::T*gluc*)]** $^{\Delta pks1}$ (3.678 kb)<br>primers: T*niiA*-*pks1*-SH5F/T*gluc*-*pks1*-SH3R<br>template: pNAH-OGCGc	**[(natR)–(P*gpdA*::*gfpN*::T*trpC*)]** $^{\Delta phs1}$ (3.529 kb)<br>primers: T*niaD*-*phs1*-SH5F/T*trpC*-*phs1*-SH3R<br>template: pNDN-GGNTn	**pAMA/tRNA-*kppks1*** ^{PS2}**-<br>*kpphs1*** ^{PS1} (circular)

Plasmids used for transient CRISPR/Cas9 or as templates for the isolation of donor DNA were cloned in this study (Supplementary Table 3) or were published previously: pAMA/ribo-*kppks1* ^{PS2}, pAMA/ribo-*kpphs1* ^{PS1} (Voigt et al., 2020); pNDN-OGG, pNDH-OGG, pNAH-OGCGc (Schumacher, 2012).

3 Supplementary Sequences

Supplementary Sequence 1. *K. petricola* galactokinase 1 (GenBank: OM802156).

***Kpgal1* locus** (ORF plus 1.000 kb of 5' and 3' noncoding regions); **P*gal1* (0.658 kb) is shaded blue**

...CTCTGAACGGCATTGGGGACACGTTATTTTGTCAGGAGTCGGACCTGATCGTCCACCGCCTGAACGAGATCGTGGGGATTCTTGGTC
GGCTACCCAGTCTAGTAAACATGCTTCATGGGCTTGGAGGCTGCAGGAGCAGGGCGAGCGCCAAGCGGTAGGAGGGTTCGGAGGCGAGGG
AGGATCTTCATCTTTGGTTGCGAGGCATATCCAGCACTTCTCGAGAGCAGAGGTAGATTGCCTAGTCAATGTTGGAGGTCGTTCAGGCTG
ATCTGCGATGTCTGCCGTTGAGTGATTGACGAGCTCAGGCTCAGATTCCGACCCTGTCGGAGAGGAGGAGGCCATgtttggcggtagtgt
ggcggggatctaaggacaggaggctgcttggccgctgcctgctattggaacacaggtagatggttgagcgttgcctgttaacacaacgtt
cgccgtgtcgtgacgagccagcgattgctagtgcttgacagcaagcatgcgacgggcagcacgcagcgcaccaaagccaatggagtcaac
aatgacgaatgcccgagccccaagcctgaggccgcagatcggcccccttacaagcaaacaaaacaaatcgtcgaaacggtatcattggc
tcatccctactccgacctcgtacgttacaatcccgcaacaaagtggatcatggttgctgagttgcccgccttgagcccgcttcaagttga
tgagttttatgagaatcccaagtgtcaccgtatagtacttgtagtagacggtaagctgcgtgacctggacctgacttagccgttggaaag
ctcaagcgtgagacatcttagacgcgactgccagagagcgcaacccagcttagaaggcccgaatcctcagcctgaatttgtcctcaacac
caggctcgacaagctacattgatccaacagaaactttgactttccgtcttctacacacggctcgtaattgcagatcccaatatcaggcta
ggactcgtacaac**ATG**ACGAACGGGGTGTCAAGGGTTACGTCCCTAGACGACATATATTCAAATGCGACACCTGGAGTCTTACCAGAACA
GAAAAGCCGAATCAAGAATCTTGTCGACTCATTCAGAAATGCACACGGTAGCAATCCGGATTTCATAGCCAGAAGCCCCGGTAGAGTCAA
TATCATCGGAGAGCACATCGACTATTCTTTGTACAACGTGCTACCTACAGCAGTGCTCAATGACGTGCTAGTAGCCGTCAAGGTCAACGA
GTCTTCGTCAGAAACGACCATCAAGGTCGCGAACACAAATGATACAAAGTATCCTACAGCACAATTCCAGGTACCTCGAGAAGGGGAGGT
GCACATGGGCGAGCATGATTGGTCGAATTATTTCAAGGCCGGACTCAGAGGCTCCATTGAGGTTTTGAGAAAAGACCGTGGACACAGCTT
CCATCCAAAGAGTATGGACGTTATGGTAGATGGCAATGTCCCTGCCGGTGGGGGGTTGTCGAGCAGCGCAGCCTTCGTGTGCGCTTCAGC
CCTAGCCGTAATGGCAGCCAACGGCCACGAGGTGTCGAAGCAAGACTTACTTGACCTCTGCATCGTGTCTGAACGGTCTGTCGGTGTTTA
CTCGGGCGGCATGGATCAAGCCGCGAGCATATTCAGTGAGAAAGGTTATCTCTTATACTGCCGCTTCTTTCCCAGCTTCTCCGCCGAGCA
TGTGCCGGTACCAGCGGCCGATCCAGAGATTACCTTCCTGATTGCGCAATCTTTCATCACTTCCGACAAAGCTGTCACAGCCGCCAGACA
CTACAATTTGCGAGTGGTCGAGGTAACACTCGCGGCTGTTACATTAGCCAAGTTCTTCGACATTACACTCGACCCTGATTCGTCATCGCT
GGGCTTCAGCATCCGCAACTTCCAGGAAGAGCTTATGAAGAAGCATGGTAAAAGAGATCTCGCCGAGGATAAACAGCTTGACATGATGAT
CAATGTGGTTCGGGAAAAGCTCACACAACAGTCATACAGCAGAGCAGACGTAGCTCGCATACTCGAGATCGACGTATCGGCTCTCGAGAA
GGTCTACTTCTCCAAGTTCGATGTCGAGGGTGACACGTTCAAGCTTCAACAGCGCGCGCTGCACGTTGTGGCGGAGGCTCGGCGTGTGGT
TGCTTTCAAAGATGCGTTGTCGACAAATGCGGGCAACAAGCTAAACCAAGGTCAACTCAGTTACCTTGGCGACCTCATGAACAAGACACA
AGACTCGTGCAGAGATGTGTACGAATGCTCGTGTCCCGAAATTGATGATATCTGTGCGATTGCCAGGAAGAACGGTGCAATGGGATCCCG
GCTCACAGGCGCAGGATGGGGCGGTTGCACAGTACATCTAGTGCCTCAAAACAAGGTCGAAAATGTCACCAAAGCGTTGAAGGAGGAGTA
CTACAACAAGAGGTTCCCGGACCTTTCTGCTGATAAGATGAGAGACGCGATTGTCATCAGCAAGCCTGGCCAGGGAAGCTCGCTCATCGT
GGGGGAAGCACTGAATGTG**TAG**atgataggacatgcatcgtcttatgctcgtgtcctgaccacagtccactttgcaccgggtccctggtt
gtggcacttatcccctggctcgacatgtttgcctattatcgtcatgcataaatattggtgaaagctacgcgctcttgccacctcgctaaa
gaaccgactagtcgtcgatctacctgttcctaaggcacgatgcaaatcatccgttagtcggaagtcgaaattctgccagtgcaatcgaga
ggcggccagtctacgtaatgtcggtggtttctacttccaaccgccgatgcttggcattttgttggccgcccgcgcctggcatttccaaat
tttgtcccatcaggcctctcttgtccctccatacaatgtttggagaggcgtccagtcttacatgcattggtcttgtgcttttctcgatga
agcatagctacgcctgacttgcaccaggctaagcctggttacagtgcttattggtttcattcggtgtccgatgcgcctactcctacttgt
agccaggccccgtccccttcatcgcgtcaaaccaatcctcttccatcatgacagacccctcatgcaccctcccatgtggcctagggtctc
gaaaggcccgccctcgctccacatgtcggcgctctaaacagggacgccgacatcagcagccagtcatgtcttcggccaccatgttcactt
ggtctaaaatgaggtgcaggccgttccgtgacatacgtgctacgcggtgaggagtataaccaagccatctggaaagtttttcaaggcaat
cagttatcacacactgtctcgtgctagtccatactacaccctgactgtactgcgattgtagtaatctcgcacagtcccatacagcagctc
tcacatatctccaccggcgcagcATGGCAGCTCCCGCTCCATCCATCGTTGTCCACCAGGCTGGCACCGCCAGCAGAACGGTCCCGACCG
CAGTCAATAGCAACGCGGGTCGACCACTTACA...

KpGAL1 (512 aa)

MTNGVSRVTSLDDIYSNATPGVLPEQKSRIKNLVDSFRNAHGSNPDFIARSPGRVNIIGEHIDYSLYNVLPTAVLNDVLVA
VKVNESSSETTIKVANTNDTKYPTAQFQVPREGEVHMGEHDWSNYFKAGLRGSIEVLRKDRGHSFHPKSMDVMVDGNVPAG
GGLSSSAAFVCASALAVMAANGHEVSKQDLLDLCIVSERSVGVYSGGMDQAASIFSEKGYLLYCRFFPSFSAEHVPVPAAD
PEITFLIAQSFITSDKAVTAARHYNLRVVEVTLAAVTLAKFFDITLDPDSSSLGFSIRNFQEELMKKHGKRDLAEDKQLDM
MINVVREKLTQQSYSRADVARILEIDVSALEKVYFSKFDVEGDTFKLQQRALHVVAEARRVVAFKDALSTNAGNKLNQGQL
SYLGDLMNKTQDSCRDVYECSCPEIDDICAIARKNGAMGSRLTGAGWGGCTVHLVPQNKVENVTKALKEEYYNKRFPDLSA
DKMRDAIVISKPGQGSSLIVGEALNV*

Supplementary Sequence 2. *K. petricola* white collar-like 1 (GenBank: OM802157).

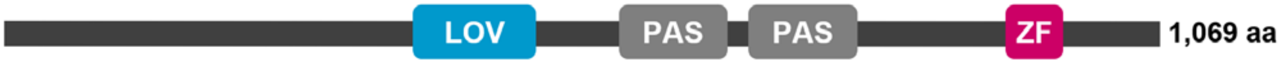

***Kpwcl1* locus** (ORF – shaded blue – plus 1.000 kb of 5’ and 3’ noncoding regions)

...acttgacttgcacttgacatgcaagtcatactgtagagtataatgattgctgtaaggcagcacaatcgacatgttttctgttgaatc
gtttctgctgttgccgtcgccaccatcatgttggttgacctctgtttgttctcggcttcctggttgatcacaagtggttttggtgattga
gctgcagcagaagcatagatgcatgctccgacacaactatgtacaggaagacgggtaggggcttctacgagttgggtgcgatcaacatcg
acttcatgtgcgagatcgtcatggcattctcgagagtcatgggtttggcatgacggcccaaggatgatacctacctaggtactgcaggcc
caggcggcccggccttggagctgtccacttttctcgcctggcctgtcgggacagtgcggtcgcggcacatagtaacaacgaaagccgtga
agctgcgatttattcttactgggtcagcacgtcgagacgagatgcattaacttcacgatgcaatctgaccagtgtcaaccagtggacttg
cttcacctaaagtgtttggatagctcagtctatgacgaggattgcataatggcaggagtccacctaaccacattgaccaacgatagtgct
tcccaggcacgcgtcacccatgaatcccgacggctgaagttgctgctttggcggccatgttttagcctctcaggcctttccgcagtggtc
gtgggctttctcggcctgctgaaccctgacaaggccgactgcgctttactcgagcaccatctgctctgcgcctcgtccaactagaccacg
ctgacgccaacattctgacaccagcggcacaggtcgtggagaccgccgaggctcgacgtgccaaagacagcactcacggtcgatgagcat
ctgagagatgtacgacaagttttcacctctgccgaccgagaccttgaatcctgttcctggagtgcgcaattaactcgacggctgcgaccg
tccgctgttcgcc**ATG**AACGGCATGTCGAATATGTACGATCAAGCCTATTCTGGCCTGGAGTCACAAGCACCCATGGACGGCTACCCGCA
GGACATGCCTCTGGAGGGGCTGGACACCAGCATGATGGCACATGGTGCTTCGCAGACTCTTGATCAGATCATCAACCAGAACAACCAAGA
GTTGATGAGAAGGCGGCAGACAGCCTACCAGCCTCAATTTCGTCCCACCCCTGATGGCGCCCAGGATCAGGCCCAGGAACAGGCTCAGAA
TCACGCTCGTCGAGCGTCCATGATTGAGTTCGGCTCTTATAACAATGGCGACCTGACCGACTTCGAATTCGACCCAGCTCCCTCGCATCC
CGCCATGTCAACTCAATTCACGAATCCGGCCTTGATGCCTCAAAAACCTCTCGATCCCCGACGAGTTCGCTCACGTGAAGATCTATCACT
TGATACCAGTTTCCCCCAGATGCCTGACTTTCAAGCTGGTTCCACGAGTGCCTACCCAGCTTCCATGTTACCTGCTACAGCCATGGGCAT
GGACGGTTCGAATGGCTACATGCCGCACAATGTGAGCATAGAGGGCGATTTTGATAATATGCCGGCGAACATGAGTGCTACGACCATGCC
TTCGAGCCAGGCGCAGCAGGGCATGTACACCATATCGCCTGTCGCTGCGAACTTTCCAATGCAGTATCCAAACACGGGCCACGACATTGG
TGGAGGGACTCCAGACAACAAAAACCTGATGCGTATGCATCAGGGCACGGCGCCTAATGCCTCTTCACCCCAGAGATTCTTTGGCAGGTC
GCAAAGCCAGATACGGAGGAACCCCATGATGCCGAGCCCGTTGTCGGTGTCTGCACAAAACTCAAGCTCGAGCACGATGGCCTCGCCAGC
ACAGATGCCCTCGACACCTACCATTGCACAGAATCAACAGTCCCAGTCCCGCCGCGGTTCCCTAGATATGCAGTCAAACAGCTATCCGAA
TCCAGCGTCCGATAATACGCAGATGGATCAGGGCCAATCGGCATCTCACTTTAACGCCTCAAGATGGACAAATGCATATTCAGCCACTGG
CTTTGATATGCTAGCCGTCTTGATGCGCGTCGCGACCAGGCGCAACCCACAGATCAGCATTGGGCCGGTCGACCTTTCTTGCGCGTTTGT
GGTTTGCGACTGCCAGAAACATGATTTGCCAATCATATACTGCTCAGATATGTTCGAACGTCTGACGGGCTATTCACGTCACGAAATACT
TGGTCGCAATTGCCGCTTCCTACAAGCGCCAGACGGAAAGGTACAGTCGGGCATCCAGCGCAAATATGTGGACGACAAATCGGTGTTGTA
CCTGAAAAACCAGATCAACAAAAGAGCTGAAGCACAGCTATCCCTAATCAATTATCGAAAAGGTGGACAACCTTTCATGAATCTACTGAC
AATGATCCCCATAACGTGGGATACTGGGGAGTACAAGTACTTTGTTGGTTTCCAGGTCGATCTGGTGGAGCAGCCAAATTCAGTGTCGGC
GAAGAATCCAGATGGCACATACGAAATCAATTACAACAGAAGTGCTCTACCAGCATACCGACTACCAGCACCCGATCCTAGTAGTGGCCT
CGAGAATATGGGCGGACAAACCATACCTAGAGAGGAAGTTTCCCAGGTTCTAGCCACCATCGGAAACGGCGAGACAGACCTCTCAAAAAG
GATATGGGACAAAATACTGCTAGAGAACACGGATGATGTGGTACATGTGCTCTCGCTGAAGGGCCTATTTCTCTACTTGTCGCCGGCTTG
CAAGTCGGTGCTGGAATATGAGCCATCAGAGCTCGTAGGCACAGCATTGTCAGCGGTATGTCACCCTTCGGACATCGTACCTGTTACGCG
AGAGCTCAAGGATACCACAAACGGGTCCCCAGTGAATGTCATCTACCGGATACGCCGGAAAAATGCTGGTTACACCTGGTTCGAAGCACA
CGGTTCTTTGCACACCGAACAAGGTAAAGGGAGGAAGTGTATTATATTGGTTGGAAGACAACGGCCGGTGTTTGCATTAGCTCGTAACGA
TGTTGTGTTAACCGATGGAGCTGGCGATGCTGAACTCTGGTCGAAGATGTCCACGACTGGGATGTTTCTCTACGTGTCGTCAACGTCGAG
GTCGATGCTTGATAGGACACCTGACGACCTTGTCGGGACGAGTATGCAGACGTTAATGCGACCTGAATCACGCAGGAAATTTGGCCGCAT
GCTTGAACTTGCAAGAATGGGCGAGAGGTCAACTTTCAAGCATGATCTCCAGAATCGCAGGGGTCAAGTACTACAAGCGCAGACCACGAT
CTATCCTGGCGATGCTAAGAAAGGGTTCAAGCCCACTTTCCTGCTTGCTCAGATGCGTCTTCTGAAGATGACCCGCTCGATGCTTCTCCA
ACAGAAGAACAACACGTCGATACCTCGTTCTGATGCTTTGTCAGACATGGGGCCAATTGCGTCCACTCCACAACCAATCGCTCCGAGCGA
GCCTCAGGAAAAAAGCTCCCCGCAAGTGGGCGGGCAGTCGCCCTCGGCTCAGGATGCTTTCATTGGAGGATCGGGTATTCTCACAATGGC
AGGCTCAGGCGGTGTTCCTATCGGTTCACAGGACGAAGCTCTTGCTAGTGAAGACAACATATTCGATGAGCTTAAGACTACTCGAAGCAC
AAGCTGGCAGTTCGAGCTAAGGCAAATGGAAAGACAGAACAGGCTACTCGCTGAAGATTTGCAAACCTTGCTTCAACGAAGGAAGAAGCG
GAAAAGGAGGAAGGGAATCACGAATCTTGAAAAAGACTGTGCTAATTGTCATACGAGAGTTACGCCGGAGTGGCGGAGGGGTCCAAGCGG
ACAGAGAGATTTGTGTAACAGCTGTGGGCTAAGATGGGCTAAGCAGgtaagtcggtttcctgaaatatgtaccacgtttcggcagcactg
actgatgtgtagAATGGCCGCGTCTCCCCACGCAAGTCTAGTATGTCAGACAAGTCCGCAGCGTCGCCCGGCCATACCGGCAATGTTCAA
CAGCGAGCAACAGGCAACCCTTCTGGTGGCAGCAAGGAAAACAGCGCTGACGTGAAGCCTGGTCCTGCGTCGATGGCGGAGGAGAGCAGT
AAAACTCCGACACGGCTTACGCCTTCGCGGATCAAGAACGAAAGAGACGACGACGCAGAGGCAAGCAACATGCCTACCATAATGGAAGAG
GGACCAGAACCTCCTCAATCTGACGTGTTACCTACA**TGA**cacctctctgtcttgacctccaacagaaaaatgactctcctcgctctgcca
aacgcatacggtgtggcgcccgacacagaaagagttgtggttacgcaggcagagctcccacgactttatggctcccctttgttatcttga
cgacgtagagaagaacggagatacccttttgcgcattggttgtacattcagcacgcacggcgttgggtaggagcaagcacaagcaatgtt
agcaggcacctcacagcaacaaccaattttaatcatactacctcacggcatcaaccatgcgcagttctcgcctctgctgcccaacagttc
tgcgctgagtttccacccctagccatatctgcctcgagtcacaacctctttgacagtaatcgtatctcgatgtacaacacgcgacacaaa

actctgcagcgtacccttcgttagagtacgtacatacacggccaaagagatccggaccgtctcgcatgcagccatcgccagcagtccaat
ggccctatacccatggcgtcgcagatacgttgtcccttgcggttcgtgaacagcgccgagttcagctatcagcgcgtttctcatgctgtc
cttcatcaccttgcgctcgatgccacagacagagcactacccggcgacaagcgtgtctcaggacacctcctggcatacctcgtgtctcca
acgatagtatggtgggtattttatgttccaagagaggaccaatattcggcgtagccgaatatgcttcagaaaagtgtgttaacagtacct
tgtttctcccccagtagtcgtatctagattactgagcaaataagcaagcaagcgaagttgtccgtgctcgcgagcacggccatgaatatc
agaccagcattttcaacctctatttgttctgcgcgtccctagtcaatgttgactgtagtctgtacattcagacctgggagttggttgctg
ttcgcttcactagatgtacttttgctgtctagaacatacttgcacgatc...

KpWCL1 (1,069 aa)

MNGMSNMYDQAYSGLESQAPMDGYPQDMPLEGLDTSMMAHGASQTLDQIINQNNQELMRRRQTAYQPQFRPTPDGAQDQAQ
EQAQNHARRASMIEFGSYNNGDLTDFEFDPAPSHPAMSTQFTNPALMPQKPLDPRRVRSREDLSLDTSFPQMPDFQAGSTS
AYPASMLPATAMGMDGSNGYMPHNVSIEGDFDNMPANMSATTMPSSQAQQGMYTISPVAANFPMQYPNTGHDIGGGTPDNK
NLMRMHQGTAPNASSPQRFFGRSQSQIRRNPMMPSPLSVSAQNSSSSTMASPAQMPSTPTIAQNQQSQSRRGSLDMQSNSY
PNPASDNTQMDQGQSASHFNASRWTNAYSATGFDMLAVLMRVATRRNPQISIGPVDLSCAFVVCDCQKHDLPIIYCSDMFE
RLTGYSRHEILGRNCRFLQAPDGKVQSGIQRKYVDDKSVLYLKNQINKRAEAQLSLINYRKGGQPFMNLLTMIPITWDTGE
YKYFVGFQVDLVEQPNSVSAKNPDGTYEINYNRSALPAYRLPAPDPSSGLENMGGQTIPREEVSQVLATIGNGETDLSKRI
WDKILLENTDDVVHVLSLKGLFLYLSPACKSVLEYEPSELVGTALSAVCHPSDIVPVTRELKDTTNGSPVNVIYRIRRKNA
GYTWFEAHGSLHTEQGKGRKCIILVGRQRPVFALARNDVVLTDGAGDAELWSKMSTTGMFLYVSSTSRSMLDRTPDDLVGT
SMQTLMRPESRRKFGRMLELARMGERSTFKHDLQNRRGQVLQAQTTIYPGDAKKGFKPTFLLAQMRLLKMTRSMLLQQKNN
TSIPRSDALSDMGPIASTPQPIAPSEPQEKSSPQVGGQSPSAQDAFIGGSGILTMAGSGGVPIGSQDEALASEDNIFDELK
TTRSTSWQFELRQMERQNRLLAEDLQTLLQRRKKRKRRKGITNLEKDCANCHTRVTPEWRRGPSGQRDLCNSCGLRWAKQN
GRVSPRKSSMSDKSAASPGHTGNVQQRATGNPSGGSKENSADVKPGPASMAEESSKTPTRLTPSRIKNERDDDAEASNMPT
IMEEGPEPPQSDVLPT*

Supplementary Sequence 3. *K. petricola* white collar-like 2 (GenBank: OM802158).

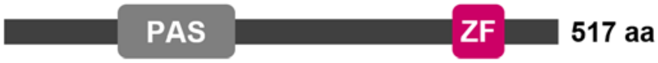

***Kpwcl2* locus** (ORF – shaded blue – plus 1.000 kb of 5’ and 3’ noncoding regions)

```
...TTTCACCCTGCAAGCCTGGGTCGTGGACCCATGCTCCTTTCAACCCCGCACCTGGAGGTAGGCCGCCTGTTGTGACAAATTTGAGAG
CCTCGATGATGGTGGTTTTTCCAGAGCCGTTGATGCCGGCCACGAGGGTTAGTGGCTTATCGAACCTGATACTCTCTCCACGCTCAGTAG
GAAAGGCACGCACACCCAATATCATCATCTTCTCGATGGAAGACATagcgatggacggcctgcaaagcagggcttggatgaatcaatatc
tgacgattattgctgtgagagacgaagcgaaggactaccatctgtcggagggacagattagtgattgggtagggtgcagttctagtagac
tatcataacgtctgcttctacctgcttctcgtctggtgattcaagcgacttgcactgagcaccaacgtacgacaaagtatcgaggtgacg
ctcaaatgacaggcaggcagacagacaggtaggaacgcggctgccgttagaacgcgtcgcgctctccatcacgtgcaccacgtaaagtgc
ttgcggaaacctggagtggcgcgagcagagcaaacagcacaaaattctggccccctaccgtagtcagcatctttggtcgccaactcgatg
ggcggcatctcccgatccaagacttttcccgtattttcgatcaccgattagcctcgccaaagtgacggattcataagaaacaccacagac
aatccacttcctgtattcatcgtcgatctttacttgcgttctgcagctggcaaggccggaaaaactatccaggctcttgtgctactgtcg
gtacctcaatatcccatttgtgcgcttctggctattccacttccgccgactacgcagcgctcttactggtcccatgcatgctcccgcaaa
gcagaggagctgctgcatggccattgacgagcggatagaccaagacgttcagctcagtggagtcttgtgctgccctgcccagtactgaat
caggcaaggggtcATGGATCCTTACAATCAAAACCCTGGCATGTTGAACATGTCGGGTTTCAATGCTGACATGTTCAATGCGGGCATGGT
GGATACTGGCGATTCTGATATGAAGTCGTATGGCGATGACATACAGATGGATATGGGCGACTTGTCCGCTGTGCCCTTCAACAGCGTCGA
GCCAATGGCGGGCTCGATCCCAGAATCAGAACACCAAATGCAAGGTGAAAACAGCATGAACATGACGGGTGTGGGAGCCGGTCCGCTCCC
TACGGGCTTCGGTGTCCACACCGGAATGGGAAGTACGTTGACAGAATTCACCAAACGGCGCAACTGGTCTCAGCGTGTTATCGAAGAGAT
CAAAGACCTCCTCTATGTCCTGACGCCTGATGCTCGCTTTCTCTATTTGAGCCCGTCGACCAAGCTGTTGACAGGTTACGACACGACAGA
ATTGACTGGCAAGTTCATCCACGACTTCATCCATGGGGACGACAACTCCCTCTTCCTGCGTGAATTCAACGAAAGCATAGCAACGGGCCA
CGTCCTCCGCATGTTCTACCGTTTCCGCTGCAAATCGGGCGTCTACCTAATCTTCGAATGCCACGGTCACCCCCACCTAACCAACGACGT
ATCGCAATTGGAAGTATCAGGCGGGCCTCGCAATAACGCTGGGTTCTGCGCCGGCTACTTCATGATGGTTCGCCCCTACCCCACAAAGAA
TGCCGTGCTCCTTGATTCCTTCCTCGAGCACAAACTCGAGAATGAACGTCTCCAAGCGCGCATCAATGCCCTACGCCGAGAAGAAGCCGA
GGAGGCAGAAACCCAAGTCCGCCACTACCACAAAAAAGCAGGAGGCGGTAGCTCTGTAACTCCTTCCGGCCAGGGCATGGACATGAACGG
CACACCCGGCGGCGATTCACCCAGCATAACATCTGATCGGCGCAGCAGCGAGCTCGGTTCCATGCCGCCACCCGCCAAGCCAACCATGAG
CAACATGGCATTGACCCGTGAAGCGCTTGACGAAGCCAACGCCTTCGCCCGACCAGATAGCATCACCGACAAGATGGCCCGCTACGAAGG
CACAAGCCACGTCGACAGTATCGAGATGCTGACTGGTCTGCGCTACCGCGAGGGCGAGCGCGCCCAAGGAATCAGCACGGGTGGCACATC
TCCCGCTTTAATCCGTGGCGACGCAGGAATCGACATACCCGCCGACAAGAGCGACCCACGGTTCGGGGGAGGCTACATGGACAAGAAGCC
GAAGAAGGTCAAGTCGGCCGACGAATATGTGTGCACGGATTGCGGGACGCTGGACTCGCCTGAATGGCGGAAGGGCCCGAACGGGCCAAA
GACGCTATGTAATGCATGTGGCCTCAGGTGGGCCAAGAAAGAGAAGAAGCGAGCTGGTTCTATGAGTGGTGGCACTACGGGGCTTACAGC
TGGCTCTATAGGTAACACCGTTAACAGTGGGGGTTCAGGTGGGAACACTAGCAATGCCACAGTTGTACCACAAGCAGGAATGGCTCCCAG
CTTGCCAACAGGACACTCAACTGGTAGTAGCCATTGAaggaagacgacttaagtaactcaaccgcgcagcacgtccatcgccacgacgca
gacttcttcccgggcaagcacgatcagatgacgagaaagacgaacggactggagagacagacttatccaggacgtgagaacagtacagtt
ccagtgggctcttccgtactccagaaaacacgatgcagcgacagcaagtgagcgaaaaggcaatgcgtgatcagcactggccccagctct
accaataatgttacgacagcgcaagcgcaagcacaagcacaagcgatcaaggaagaggtcgcaaatggtaaagtgccctcaacatccggg
ggcaagccaaatccagccgaactagtaagagcgctcagatcacaagcatgtttgcgttgagacgacgatccgcaagtccacagacagaga
tctgtacctcccccgaacccgacaaagacgcgcgctgtgcccagcgctggacaacaccgctagagatgatatacaccccccgcgtgaggga
tcgagtagggcgcagcacggctatccctgccttctgcatccttcagcgcgtgcaggggagagctcaagctgcggcaaaggacgatgtcgc
gggcaacgaagcaagccagggttcaggcgactgtgcgctgcccggataccggcgatagaagtagtgcgtgtggcaccgggatcgaggggt
tggatctggttgatattttgagtgtgcgaggatctagatagtgcccggtaaggcgtttggagcaagcaggcagtttgggcatatatatag
cattggattcaatcctatcttgatggcgggatacttttctattctgacgtggtgattgatttgatgatgtttctttctcttcagcatgct
agcacaggagcaagtagtttgtgaaaaaaagtcgggatagaatcgggatgctcgaagcttgtctttgtcgccagccaagccataactcat
ctaagatgtacctatgtagcatgggtccaggttcaacgccaggtgcc...
```

KpWCL2 (517 aa)

```
MDPYNQNPGMLNMSGFNADMFNAGMVDTGDSDMKSYGDDIQMDMGDLSAVPFNSVEPMAGSIPESEHQMQGENSMNMTGVG
AGPLPTGFGVHTGMGSTLTEFTKRRNWSQRVIEEIKDLLYVLTPDARFLYLSPSTKLLTGYDTTELTGKFIHDFIHGDDNS
LFLREFNESIATGHVLRMFYRFRCKSGVYLIFECHGHPHLTNDVSQLEVSGGPRNNAGFCAGYFMMVRPYPTKNAVLLDSF
LEHKLENERLQARINALRREEAEEAETQVRHYHKKAGGGSSVTPSGQGMDMNGTPGGDSPSITSDRRSSELGSMPPPAKPT
MSNMALTREALDEANAFARPDSITDKMARYEGTSHVDSIEMLTGLRYREGERAQGISTGGTSPALIRGDAGIDIPADKSDP
RFGGGYMDKKPKKVKSADEYVCTDCGTLDSPEWRKGPNGPKTLCNACGLRWAKKEKKRAGSMSGGTTGLTAGSIGNTVNSG
GSGGNTSNATVVPQAGMAPSLPTGHSTGSSH*
```

Supplementary Sequence 4. *K. petricola* intergenic region 1 (*igr1*) (GenBank: OM802159).

Kp genome v1.0: Contig 1: 474,324 – 484,324 bp

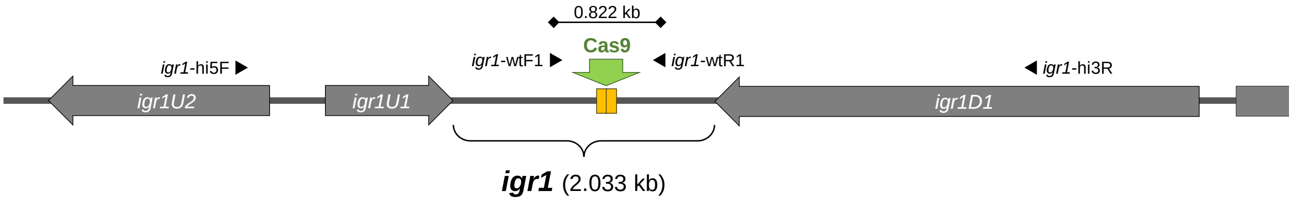


```
...tcatgctgagacggcgtcgttcggtcgaccaagctggctgttcagggcacaccgtgatagcgcgagcaccgtaatctcttgagagca
cagtcggctagatacacaataagtccgctgccgagatcgacctacggatatgtaagaattctacgagatgcttcgtttgggcgctccaaa
gcgcctcagtcctcagatacacaattgactgccactttgatgttttagaggatcgatacattactttatagtagtctatgccataccagg
cgactagtgtctatgacaaatacaggttcctcggggttattaggttctacatgatctgcaggcggtaacctgttccaTTAGCTGTTGGAT
AGTATTTCCTGTGGCCTCCAAGTGAACGCAGTTTTAGATTTCGCCTCGAACAAATGATCGTTTAGAAATTCTGTCGTTCGCCGGCTCCGT
GCAACCCTGCAACGAATTGAAAATGTCGGATTTGGCTCGAACTGGTACGCCAGAGTCGGAAATGCCACAATCCTGCCCTGGCAGGTCATC
ACTGGTAATGTAAACCTGGCATCACCAGGTGCGCTGTTATCCAGTCGTTTTTTGAACTTGTCTCCGTTCGGAGACGTTTCCAACATAGCC
TTCACAGCAGGCACATCTTCGGGTTGATACGGTCTGAAAATGATGTGCTCAAGGAAGTTTTGTTGCGATGATTGAACACCGATCCAAAAC
CGGCGATCCCAAAGAAACCAGCTGTACCGATCTTTCCCGGTCTCGCCAGACGTAGGCTTCAATTTAAATATGTGAGTGGCTGCGGCCATC
TCTGACCGTCTGAAATTTCCCCGACTAATCATGAATGTCACCGTTTTGTTGGTCATGTCCTCATTGATGAGCCTAAATACGGCACCTCTC
ACTGTAATGGCGGCAGCCGTCTGGGTTCCTGATGAATCTCCTTGAGGCTCTGATAGGTATCGCGCAAGCTCCATCGTATGGTCGCTGTTT
AACAAGAGTGGCCAAGAGTCCGAGTGAGCAGGACTTGCCAGCTCAAATAGCCTGGCCATCCCATAGGAAAAACCACGAAGCTCAGCAGGG
GTACGCAGATGCTTGAACTGCTTTAGTGTGATTTTGACCACTCCGGTATGACTATCCAGACGAAACTTCAGAATTTGATCCCAAACATGT
TGGGAACGATCTTGGGAGTCTTGCGATAATGTGTAGGCACTCCACTGTAAGGCGCGAAGGCTCTGTTCTGTCAAAGCCTTGGGTAAAGAA
TAATTCGCCCTAAGGTAGCGGACGGCATTACGGGTGGTGAAGGTCGGATCAAGGTTGGTTTTGTCATCCACATAAGGGATTGAAAACCTG
CTGCATGTAGAAAGGATGGCCTGCTTGGGAAAGATAAGCAGTGGGCGTAACAAGCGCTGACCTTGGTATGGCTGCTTCTCCCCAATCAGG
ATTTCGTTCGTAGATCCGGTAGCAACGCGTTTCGCAGGTACTAGCCCATTCGGTAGTAGGTTGGACTCACCACGGACAGCAAGAATGCTC
TCACCACAAGGCATGGCGGATATTTGGGCCATGCCTAGGAACCCTAATACGTTGGGATTACGATTCCTCAGTAACCGTAGTAATAGTGTC
TCCGTTTGATCATCCAAATGATGTCCTGCGAAGAGATTGGAGATCTCGTGATCCCTAGCAGCTCGCGCGATGGCTTGGTATCGGCGTGTT
CGGGCGACTTCCTCGAACCCACCTTTGGCCAGATTTTCGAGCTCACCCGGAGACCACGTCAGTTTGAGGATTTTAGAACCTATACCTAAG
CTCCGAAGATTCCTCTGTACTTGAAGGGCCTCAGCAGCGCTTTCGGGACGGGCACCATGGTCTACAGTGAAGGCTTTTGGTGCGAACAGG
CGTTGATTTGGGATATATGGATGTTTGAGCGCCTGCAACAGAAAGGCCATTACCATTGAGTCTGCCCCACCGCTAACAAGCACGCCTACT
TTGTGTGGCCTGAAAGGTAGGTTCCTGTGCCTGACGAGATAGGGGGCAAGCTTGGATAGGTGACGTGCGAAGCCCTCGAGCTTGATATAA
TCCGCCATactgtatgttcctgctcaaataacgtgctttgaaatagctatcttggatttggaggagggtgcgtgaatataaagagcagtc
aggggtcaagcatgagaccggcacataatggagagcttgtcgcgattacgtcgcagaatcctgtgttcgagcagtgtatacgttgagacc
agccttggtcggatgtaccaagcacgtactgataacgttcgaagtgacaagcaacaaaatgtacgcggcatgtagaatcaagaacctggt
ggagctagacacccaaagcgatctcttttgcaatgtttgtaacagtgctacggcaagcgttccataccaaagccgcatcaaggcaaatcg
agcactctgcatccaggagagacacttcaagcaaatatcgtctttgctttgttagctatacagcctttcgtgcgaacctgtccggATGAT
CAGGATTGCTCTTTGGTCATGCCGTCAGTCGAAGTCCCCTCGTCTATGTTCGACGCGGACAACACGCCACGTTCAGAATGTCGTTCTCCA
GTCTAGGTCGGTGACGACCGTGCCGACATTGAGCTCTTGGACGATCAATTCTTTCCGAGAGTGTTCGTTTGCGCCGCAGACTCCAATTGC
ACTCGTTGTATCAGACAAGGAATTGCCTCCGGCGCTCACCAAATGGTTCGTCCACGAAGAACAAGATAGTTCCAGTTCCCGTTCGCACTC
CCTCGGACACTCAGAGCTCAATTCCTCTTTCTGGGAGGCTCACGGGGATAGCATTGTACCCCTAGAGGTCACCACAATGGCGTCTGGCGA
TGCCTCTTCAACCACATTCGATCGTGTAGATGCACCCCTAAACCTCTTGCTAGCCCACATAGAGCGAACGTCTGGCCGAAAGCAGCTTTC
TCAGTCCGTCTATCTCGCTCAGCATGACCTTCGAGACCTGCCGCAACCTCTACAAGACGATCTACCAACACCACTGCTGGTAAAGCACGC
AGGGAAAGGTGATCTTTACAGCTCCTCTTTGTGGCTCGGCAGACCACCCACCTACACTCCACTCCATCGTGATCCCAATCCAAACCTCTT
CATGCAGCTGGCCGGCACCAAGGTGATTCGCTTGTTCCGTCCAGAAATCGGTGATGCCATCTTCGACCACACCGCGAAAATGCTCAATCA
AATGCGTTCTGGGCATCATGTGTCGTCTGATGGTCATCCCAGATCTGCTGCTTTTCGAGGAGAGGAGATGATGGTAGGCCGTGAGCGCTC
GATCTTGCACGATTTGGTCTGGGAACAGCCGTCAGACAAGAATAATAATAGCGCGGTCTTACAGCATGTGCAAGAAGCCACGCTCGAGAT
GGGCCAGGCGTTGTTCATTCCCAAGGGTTGGTGGCATAGCGTCAAGGGAATCGGGAACGGCGTGACTGCTAGCGCAAATTGGTGGTTCCG
ATAAccgccaggttgaactcatgcttacagtggcgatgtctcgtcttgtggttgttaaaagtgtgtgtgcaccctagtgtggaagtcccg
aattgttcagctgcaggtccagcgccttccaaaacgattcatggcatgggctgcatgcgcctacaatgtacaggtcgtctaacccaatag
ctaatcgttgagcggcgagcccctatcctacaaaggcggtctgagagatgctgcgggatctggcctcatccaggaaggcatgggtaagag
cctgctcgttttcatcgttcccctgtcctctggaggcctcattgcagcgatggcttgaccgctcgctgcaactcgccaaacactcgatga
ttggatcatcgcaagagacgggactccaagagcgtcatgagaagataacaccctgctcttgacattcagtgcgcgctagaggagcctctg
caggctcggtgttcatgatttcacaagcccacggatatctctcacgtttagtagcgtggtcttctcggagctatggtacgtcgactagca
agtagcgaatgaggcgtcggtactagtggtattgtcatctgctgtcacgatcacggctcaaaagcgcgatgaagctcgcaaccgtcagag
gaagcagcgtgcttctcatcaggcaatgaatcatacaacacctacgctactcttgaatgtccgacctagcagtggcctatcagcggttac
cgaaggatctttgcaagggccaacatcaagcagaaccagtttgtcggatttccctggctccctgcaagacccacctccgcctctcaaatt
```

cctccgcgcagccccacgtcctgccgcattcggccatgtatcattagcgacaccccagccatgcacatgccgatgggctttcgtccacat
cgacccatccccgcatcaatcgcttagcgatatgcacgccggctttagttgaacgacatctattcttgaaaacacgaggacgtggcgggt
catcacgccacatctcccgcctcctcttagcccgttctacctccccatcttcctaccagtagctcagaccacccggacatagaatacgag
gtgccatgcaggacttgaaccagttattccttcgttctgctgccgctcggaacgttccatccggtcctgtctgatccatccactcccaac
ggaccgtactctccgcgta|gactggtcgtcgagctagtcctgttttgccagatcgatcgcgatcaccgttgagcctcactcaccgccat
gccctaccctggcacttactaggccaaaggtcgcttatactgttactatgctcgtcaagtcacctaccattccccgtgacctgtcaatct
gacacccccctgtaaatcgatcagctgaacccgatcttacgtcaaagcagtagtatccaaacgggcgtgcattgaggccaagccacccctta
cctgactcgctactgtgaaaagcccccgcctagtactgatcgctgcatgtactacaaatacatgtaccgtctagcacgctacctgttctt
tctcgcaaatgccgcggtctccacccggtgcgagcagtactttcgtaccctgcgagatactaccctggtgtgggctgcttcttcacgagt
ctaaacggacttcatcgtcgatctcatcttgagtttgtgtccgttgtacgagcgaagattacagaaccgcgggtcgatgcaacagttcat
gtgtattcgtccgaacacatttgtctctttcgaagttgtcacgaacacagtgctgtgactctgcaaacttcgtctatttactccacgcgg
cgccttgcatgatcctttgtgtgtgtgtgtgtattgaaggaaacagggcaactggtagcacgttctgtgagatgagctaatagacttggt
ttccagacacccctaagggtttgccgattgctacgtgcacaaacgatgagtagacaatccaagtcactagtacaaagtatcgagatctgc
ctgcgccatcaatctggaaaaatgcgccgttccgttgcgccactttgagctggacaccTTAGTTCCTGCTCACCTTGCCTTTCCCTTTGC
CTGTGCCTGATCGCGTCCCAGCTCCGTCGAACAAGAACGAGATAATCTCGTCCAGGATAGTGTACAACAAGTGCTCATTCAGCCGCTTGT
TATTCATAACAGCACTAATTCGTCTAGCAGCTGCCTGTGCGTTCGCTCTGCCTACCACGCTTCCCGCTAACTCGGGAATGAGGGTCCCCA
GTACTGCTGCGGCCTCCCGTCGACTTGCCTCCTTCTGCACCTTAGTCCTTGGTGGGCCGGACTCTCGCATCTTTCTTTCACCCGTCTCCT
TCTCGGCACCTGTTTCGGGGTCAGTAGTATTGGCCGGTGGCCACATCGTGTTCTTCGCTGTCTCGATGTACTTGAGCAGATTCTCCTCTG
AGAACACGGAGGAAAAACTATCACGAACTTTTCGTTCAACGGTGCCGCCAAGTAATTGGTGTATGACGACCACGACTGCGCGACCTCGAA
GCCAATTGTTGCCTCGATTGAGCTCGAATGTTTCCAGGAAAATGTCGCATATGGGCTTGACGAAGGGCGTTAGCTCCTTGTTCTCAAACG
CGCGAAGTTCGGCTTCGGCTTCAGCTATCGAGCCTGGTTCGATCATCACGTCGTCTGCCGTGTGTGGTTTGTTGCCTGCTACGGTCGTAG
TCGCGCCAGTATTGATTTGAGTGGTGGCTGCCGAGATCAGGTTCTGACCTGCAGCAGAAAGTTGGTCGATGACCGCTACGTTGCCAATAA
ATTCGTCCATACCATCTGTGACCGAGTTGTATATTCGCGTAACCATGTCTTGCGCGTTAGCCGTATGGTTGCCTGCCTTGTTTGGTTGTG
TGATGGCTTGCTGCGACAAGAACGAGCGAAGCTCGCGGCTTCGGCAAACGTCAGGCATGCGAAGGAGTCGCTGAAGATATGCTTCGAGGG
CCACCCGGCGATTGTGCAAGAATTGCTTTTGTAGCTTGAGCACCATTCTCCTCCGTGGGAATTCAAGGTTGCGTGTCGATGGGTAGCGTC
GACGAAGTTTCGTATGGAGCTCATGAAACTCGCTGTAGCGGTGAGCTATCGCCCATGAAGCCGCTACCATTTGGTCGCCTGCACGTCGTT
GTACTTTGATTACATAGACTGCATGTTCTTGACCATCATCTTCACGACCCACCATAACCGAATCAATGGCAACCGACGCACGGCCATACA
AGCTGTTATCACTTTCTTGAACGATGTACTGTTGTCGTTGAAGCTCTTTGCGGCGTATCTCACGTTGCAGACTAGCTTTCGATTTTCCAA
GTATGCGTAGTTCTGCAACATTGTTCGTTAGTTCGGCTTTTCGGGTCAAGGTGTCAACCACCGCGTCTTGCGATACCAGACGCTCAATCT
CATCTGAAAGAGTTGTGATGGCTTCCGCCAGGCCCAGGTCGCCAGGAGCAGCCTGATGAATTTCTTCGTCCTTGTCTTGCTTTCCCTCGT
CTTCGTGGTCGAGGTATTCGTCTTCGACAAATTTATCCTCGTCTCCGAAGAGATCGTTGTCTTGAAAGACTCCGATTCTGCTAGCACTGC
TGACCAGTCCTAGGGTGGCCAAGCTTGGCTTGGGCTTGTCGCCTGTGGAGCTGTCAGTGCGAGTAGTCTCGAGAGCGGGACGAGGCGAGT
GTTGCTCTGACATGGGTGAGCGGAAAAGAGTATCTGGCTCGACATCAGACATGCCCGTGTTCGGACTCTGTGCCATGATGTCCGTTAGAG
CCGCTTCCATGTTCTCGATCACCTCCGGCTCTTGCACGTCGCCGTTCACGGACTCGTTGCCAAGACTCTGTACAGAAAGAGTGAGCGGAT
CGTTATAGTCATCATCGTCAAATAGAGGTCGATCCACATCGAGCGATCGGCGCACAGGAACCAGGGTTTCAGTATTTTTGGTCGGAGACA
GGCTTTTGATGTCTGAGGAGGAGAGAGCCGCCCTTCGAAGGTCTTTAGCTCTGTCCATAGGTGCTTGCGAGTTTGTTCTGCTGACTGTAG
GCGTGCTACCTTTTGGTCGCACTGGCGCAGGCCTCACAGCAGCGTTGCCATTAACATGTACGCTTTGTTGTCGCTGACGCTGTGCGTCCT
GTCCTTCATCTGACGCAAGGAACTTGTAATAGAGGTCTGATTCACGAAACTTCGGCAGGTATTTCTGTTCGAGCTCTTCGAGGGCGGCAG
ATTGGGAAGCTAGAATGGCAACTCTTGCTCCCCGGTACTGCTCCGCTGTAGCTTTACGACCAGCTTGCAGAAAATCCCGAACTGCGTCTT
GAAACTCCTTAGGAACTTTCAATTCGTCCTTCATCACATACGTTTCAGCAATCTGCGCGATGTCGAGTCGGTCAGTGACGGTCCATGTTA
TCGTTGCTGCTGATCGTTCGTCACCAAAGTCGTCCTCGAGGGGGTTGCGGAAGCCATCAACGACCACCCAGAACTGCACAAGAGTCATCT
TGTTTTGCCTATCCATGAATTCCATGAAGTAGGACAGACCAGAAGCTGTGCGCATGATATCCACTAGCTTCCAGTTTAGGACCTTCGCCG
AGGCGTCTGGCTGTCGCTCGGGAATCGAACTAGCAGTCGTCTCGTACTCGGAATTTGTCATTTTTGCGATCTTCTGGTCGAGCACTCTCT
TGCCCATCTCTAGCCGGCGGAGATACACCGGGTCCTGACCTTCTACCTCGCGCTCACGTGCAAGCTGACTAGCGATCTGGCTTCTGAAGC
GGCGCGCATCGGCGAGAGTTTTAGTCTTGCGAATGACTCGAACGAATTTTTCGAAAGCTCTTTCGGTATCGTGGGCGTGGAGACGTGGTA
TATCAGCCGGTAATTGCTTCCTTGCATCAGGCGACGTATGTTCATCGAGCGCGGCACGCATTCTCCTGACGGTTTTGCGGTCTTGCAATG
TCGTTTTGCCATATGCTTCAACGATCTGATTCCAGGTATCAGGATCGGCAAGCAGCTGGAAGACTGGGAAGAGGACAGCACACGCCACAA
TCTCCTTCAATAGCACAGCGACCACGCGACTTTGGATCATGCTTTCGTCTAGTAACATGGGTAACATGCGGACTAGCACTTTTCGGAGGT
GCTCTTGTTCTAAGGGCTTGGTATCGGAGTAAGCGAGTGAGGCCGCCGGATGGAGATGGCCGTCCTTGTACTTTGCCGCAATCGCTAGGT
CCAGCTCTTCCGACTCTGGCAGATTACGGTTCAGAGCACGTCCTCGTACCACCCGCTCAGCGTTGTCAAATTCTCGCAAGTGACTCGTTA
TGATGGGTACTACGCGCGCGACCACCATGTTTGTGACATCTTCCTGTAATATCCTGTCTCGAAGCTCTACCGCTGCCAGGCGTATGTTCT
TGTCAACCTCGTTCACGAACGTAGGACTGTGCGAAATGGTTTTGTACCACGACGAGATGAAGCTTCGAAGTGCAAGATGAACCAATTGTC
CAACGCCCTCGGAGACCAGTATAGAAGTGGGATGAAGTTGGAGAGGGTTGTACAGGAGGCTGGCCTGGTAACGCGCTTGCTCAGCTGGCC
ATATTGACGGTTTCGAGAAGGCAGGAATGGGCCTGCGCAAGGGTTTCGGAACAGTCTGAATACTCCTCGAAGTCGTCAATACAAGCATAA
GCACAAGACAGGTAGCTGCAAGGCAACCGAAGAAGAATGCGTTTGCAAGGTGCCATAGGAACGGTGCCAGCCGTACAGCAATCCCCCATG
CTACAAGAGCAACGAAGCTAGAGAGGAGGACATGCGAGTGGTCGAGGGTCATcgtcggcagtcatgacgattgtgtgaatgcgccatcag
ttcatattgacgacgcgatgggttagttgacggcgccaatcgtggtgaccggccaccacagtggaagattgactgcgatcacggtaggcg
ggtatgagcatactcgaacacctacctaggtaggtacttgcacccaaacaaagcgtaggtgtgggacagatcgtcattgccacggatccc
gtgatctaatgccttgtggcctgacttggtccatagcttcaggctgagcactaagcaacgaatcctcattATGCTCATCCCTGCTATCAT
CGGTAAGCGACCGTTTAGGCGCCCGGCAAGTTCAATGTCCATCATCAATTTAGATTTgtacgtcgatcgtaagttgtattcggctatcca
acgtcccaggtcagtctggcgtcgacaataccctagatattcgtcgacgatggatcccgctactggtttctcctacaaccgcactttcta
agcatcatcgtcctgctaaccgaggccgtcagCGTCACTATCCTTGCAAACTATCTAACCGTTAGAAGCGGCTTCAATCATCGTGAAAGC
GAAGGCCGGCGCTGCATCGAGCTACCTCATTCGCTGGCAAGAGTGAAACAATTGAGAACAATTTATGCTCGTGCGAACGCGGGCTCTACG
GAACTTCTGGCTTT...

Supplementary Sequence 5. *K. petricola* intergenic region 2 (*igr2*) (GenBank: OM802160).

Kp genome v1.0: Contig 1: 3,412,107 – 3,422,106 bp

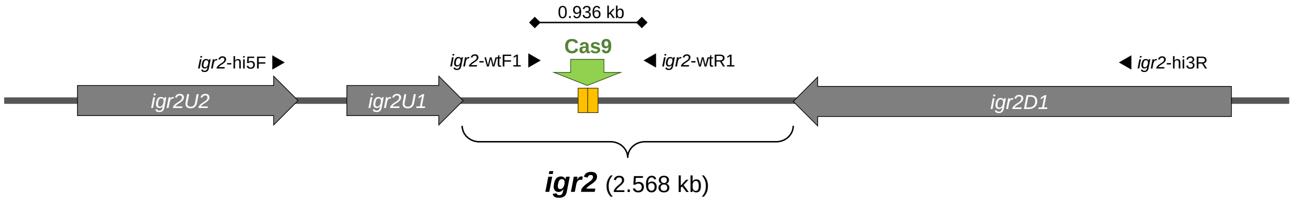


```
...actgatgttcactaaatactagacccctgccagcacaccgatacatcgttcaatcctactgtagacgtgatatcccttacaagctca
tggagctccatagcggcagtatgcggtgtaagtgctggtagttgaccgctgcagtcctgcacagctgttcgtgaacagctcctcgcacgc
agtattgcctacttagctttaaacctaccgagccccttggattgagcacgtgcgtcaatgcagctgtgctggtcggcatttcactgctcc
aaaccgacaagcatcaaggcgtattgggccgtcacctcacacgacatcaggcacacgatccatctactgcctccgacggaacccactgat
catcaaatcgcttttcgtgttttccttgattggtctccaactattcgtgtagtcccttccgattgtcgcagaactcatcatctacgtggg
atgttcggtagtctgccttctcggaactgaaatcgttatcataactcagtacggcgggagcctgcggggcccttctcgaaacctacctca
ttcagaaagcggcttgtctgtacgtgcagtcccgttgccagcttagctcaaatcATGGCTACTGGCGTTTCCATATTGAAGCCTCTTGgt
gagtcactctccgttcgcttgtgcccatggcacgacatgcacgtaattcgaccgaccgaataccaaccacgacactagaggtgtgaggtc
actctaccttacgaagccatctagctgaattggccccgtggtgtcgcctgccagagaaagcatcatgtcgtgtcgatgagccctgggctt
gcgattgagaagtgatcttacgaagatgactcgctatgcatatcagggtcgattccgtgctcgctgtcgcatctcgtgatcatgcctagc
gccaatggaggttagctaacagcaatcacagGCTATCAAAAAAGCAAACACTGCGGATACTGCAAGCAGAAAGATGGCAGCTGCTCGTAT
TATATGTCCTGCGATAGTATGCGCGTTGACCACTATCAAGAGCTCATGGACCGAGGCTGGAGAAGGTCTGGCACTACGTACTACAAACCA
GATCTGCCACGGTCGTGTTGTCCACATTACACAATCAGGTTGAGAGCTTCTGACTTCAAAGCCAAGAAGGAACATCGCTCAGCAATTAAC
AAGCTCAATGACTACGTTGTCGGACCTGAGTACAAAAGAAAGGCTGCCATGCTTTGCCCCCAACCACGTGACATCAAGAGACAGGAACGT
GGTCGCTTCGACGTGGTTGATGCTGTACACAAAGCAGAATACGAACGTCTTCCGAAGCCTATGGGCAAAAAGACGGGGGAGGCTATCGTG
CCGGCTCATAAGCTCGAAGTCAATCTCGAAGCCGACAGCTGCACCCAAGAAAAGCATGATGTAATGCTGAGATATCAAACCCAGATACAC
AAGGACCCGGAAGGGCGATGGTCTGAAGCCTCCTTCAAGAGGTTTCTCTGCAACGGACTCGATAGAAAGGTTCTCAAGGTGAAGGGCAAG
ACCATGAAGTTGGGATCTCATCACATGTGCTACCGACTTGACGGAAAACTAGTCGCCGTCGGTGTGCTTGACCTTCTGCCGCACGCTGTA
AGTTCCGTCTATCTCTTCTACGACCCCGATTACCAGGAGTGGGACTGGGGCAAGATCAGCGCCTTACACGAAATTGCACTGGCACTAGAG
AAGGGCTATGAGTACTACTACATGGGCTACTACATTCATTCGTGCACAAAGATGCGTTACAAGGGTCGATTTGAGCCTAGCTATATCCTG
GATCCTGAGAGTCTCGAATGGAATCCGCTCGACGATGACTTCCGCAAAATGCTCGATGAGCGCAAATACGTTTCACTATCTGGTGATCGA
AGGGTCGAAAACAGGCCACGGAAGGATGATGAGGACGCCGGACTATCAACGGCTGGCATACTGACTGAGTCTATCGGAGTCGGAGAAGAG
AAGAGCCCGAAAGGTGAAGATCTTGAGGTCGACTCTGATGTCTCCGAGCCTGATGACACAGAGATACCGGAAGGATCGTTGTTCGATTAC
AATATACCAGGTGTCCTCAACAAGGGCGAGGTCGAAAACTTGAACCTTGGTCACATGAAATTGCTCGTTCGGACATCCTTAGTCGACTTA
GAAGACCTTCGTGGCTGGGAAGAATGGCAGATTGACGATCCAGGAACGATCAAAGGCATTGCTGCAGAGCTAATCGCTACGACCGGGCCA
AAACTGCTCAAGGACACAGCACTCTCTCTGTTTTAGgcacttgggcggtcagaaacaaaggagacgtaagatcatcctcaagcttacgat
ctacacgccgtatatgtacgcttcgctgtaaaaagattcgaaacccggaatgcactgcacacgtatcaaccgaaagctctacgaggctgg
acctgagtgaaagagtttggcagctataccagcactggcacttctggaagcggcactgccttgacgatggccgaacaatcgcgatgtgta
gcatccgtacaggcgttggactatcaccttacacgcatggtttcaagttaggctacacgaacatacatgccacatctttcacctgtacgc
gtacttgtattgacactatgatctaacgctgtcacagatgcgtttgcattcaccagtctatcctgccataaaagctcATGGGACCCGGTA
TGGTCTTCTACACTTACTTCCTTCTTTCGTACAGAAACTCAGCCTACAAGCCCAATTCATCCTCTCTCAGATCGCTGACAGGTGCACAAT
ACACTGCGTGCAGAACCTCTTCTAGCACCTCATCCCTCCTTTCGTCGATACTCGGTCAGAGTCGGGTCTTGCCTTCTTCTCGATCCATCG
CGGACCTCAATATGTTTGTCAGACCCTTCAGTGCAATGGCTCTTGCCTCGCTGATTGCCTTCGCCAGCACTGCAGAGTCGGCAAGACACA
ACATCACATCTAGTGAAACGACCCCTTCTTCCCCGAACTGCATTCTTGGTCTGAAATCTACAACAGTCTATTGTCCTCTGAATATCGATG
ACCTAGATCCTTTGACCGACCTGACAGCCTTGCCCCTTCTCGAGGAACTCAATGAAGAATGGCGAGAAGTGACCGCTTTGGACCTGACTC
GTCATCTCGTACGAAGCGATTCAGACTCCCAACCAATTTCGCCTGTTCTGCTGGATCGTGATTCACAGGTCGGCGGAACGAACAACTTCG
CGCTCGTCTGCCTGTATGACCAAGGTCTTACGAACGATTGCCAAAAGAACTGGGGATATTATTGCGATACCAATGGTGGCTTGCGTACGA
CCAAGAAAGATAGGGAAGAAGGTTGTGACCTTTGTGAATGTGTCAACCTCAATCCAAAACCTGCCTGCATCCTCGGAATGACAGGAGTTC
TCTACTGTCAACGAAACATCCAGGACAAGTCGAAGCTTCTACCGTACGCATCTGAGATCATAGCGGTTGGGAAACTGCAGGACGACGACA
CGGGGGAGTCCGAACCGGACCCTATGAAAGGAGCGATGTCGTCTCGGAAGATTACCAACTTACCTGAGGTCCGCGACTAGcaacagcgtg
aaacacagtacacatacagcgaatacgccatggtctgcgccaacgacaaagacgctacattcacatgccagcgtaggggcggttactact
gtaacaaaacaggtaaattgatacgagacaaaatcaaaggcgaatttcgaatacacacatgcgaagacgcttgtctatgctataacatgt
ctccaaagcagaattgcattcttggtttgacgggtatgtcatactgtctacgcgatgggctggctgaagaagaccagtcaccttcaactg
aaaagataggtcatcaagacactgtgtcgctcaccaagcaatggaaagaatcagaaacgagggagtctcgccgcattgacgagatatcag
tcatatctaccgaaacgaaccacctcgcaagccacaaacgcgatgtaggaacgactttagcagagaacaaagttgatggctgtagtgtgc
atggatgaccaagatgtaactagagcctgccagagttcggattaccagtacttttgtacatccacggggcaactcagacgcaatggagcc
ttcactgatcatgtgtgtgaggaagagtgccgatgcatcgaccttcgtccgaagccgaaatgcatcttgtggctgaacggcatgtcgtct
tgtctgcgaaagcgtgatgaaggaatggccctagcggagaacaacatgcccgtggtgtgcatggacgatatagagatcaccaaagcgtgc
```

cagagctcggacttccaatactattgcacgccccggggacaacttcagcgtaatggtgctttcgttcaccaggtctgtcaggatgagtgt
tggtgcgtggatctaagtcccaagcccaactgtgtgttttgggtcaacggcatgtcgtcgtgcctgcgaacactaggcgatgccgacaac
acaacagagtcaccgtcgactctcactatagctgctagtccataac|tcacggtgatgactgtctgacgcaccagctcggccaacaggat
gtatttctagttctttccgtaggtgcacaatcaacctgacagcagcttctggtgtgactacattacttagcagagcagatagaagagtgt
gtatacttgatgcatatgtatctgagctgtctttgctagaccacccagactagaaggcgtgctactcacctttcgtctatcagttgatta
cgatctgcgaatgacattgcttcctaaggcctttatcgtagatgacaaaaagctgtagaaatagtggatcgcgtaaatgacaggttctag
taagaggtaacttcatgttagcttcagcctctactcactgaaggccccccgggtggtttgtctcaggttagcatcgcccttcaccaatag
atgcttttactgttttgcagctttcgaaataatacacctgtgatgttgagcccactaaacattctacagctcttgttgtttacgatcgag
gcctgtagctcaggtcactgtccaaaaaggatgggagatgtttccttgttcggcactccacacagtcctcgtcctccatgtttccattta
tcatgcctggcatctggcgggatagtactgacatggccgattgtcatgacttgcggggcattccggaccatctaccacggcgtagtcggg
ttggaccagtacctctcagcacccgaactcgtcttcttctggcaaagactggtcttgaacttcagatgctgcattctcagccgcgagttt
gtggtctacgaccgaaagctccgcactcgatggcagtgatgtctgtcgtcgttctcggagtgtaacgcttgaagcactgagaacgcgatc
gacggtcatcaaagaccttgaatgacatcgcatgccgatgaagttgactgcagcttgactggcatgtcttgattcacgtccaagacttcg
ggccgaggccagacggaatccggatttcagggggctccagcaaacggatgctgctcccctaggacatacccaggccaacaccaccttcg
atcacacaggaacttgacaaacattaatggacatagacgcaaaaccacctaccgctacatcatgctgtcgctgtttgctcgccgcttcat
gagccaaacgactctctctgaacgggggcacatgaatttccgccacggccatgctcgatcccgcagaaccgcgattctctccacatggg
cagaaagaactttcctagctagctggagcatagtcccctctcgaagggcccacaaggacttcgcgtgcctcggagaagctgcggacctc
ggcgccaaatttgcaaggataatactttttactcgggatttctgctctcaagttcgaacacaacacgagttcgaggataacctcgaatgc
aacccaacgatttcgaacctagagttgtttagcaagaccatctcacacagatatgcatggtaacacaagcagatgcgtagttactctaga
acttgtatgattgtccatctagctaggtcggatcctcgagcgatcgagatggccacttgcacacgggatcttccctcacctaaaaggcag
acaacaccaccctcagtgtcaccaacccaaccaactccaTCATTCATCAGATGAGTCCTCCCGGATCGTGAAACCCTCAGGCTTAGGATA
GGTGGCGGGCTCCTCAACCTTAGCTGCTCCCACCTTTCGCGCCTGTTCTTTGATCTGCTTTCCAGGTAGCATAACCTCCAACTCGGGAAG
AGCGTCGATTACCACCTGGTTGACCCTCTTGTCTGTCCATTTGCGAACCCACTTCACGGTCGCATCAACTACCGACTTATCAGCGCTTTT
CTCCAAGCTTTGGACCAGGGCTTGTCCGTCTTCGAAAGCGTACAGGAGCGGCTGTGTTTCCATGAGGTGGGTATGTTGCTCTCGCAGTTG
GTTTGCTGCCTGCGCTCTGTTTCGGTACATCTCGAGCAGCTGGTCCATCTCCGATCTTTCCTGCTCGAACTGTTGTCGGATGCTGTCCAT
CTTTTCAACGTGGTTTGTTTGTTTCTTGCCACGGATTCCCATGTTTTGCACGGCTAGGTTGACCATGAGCCCTGCTTGCTCATTGCTCAA
AGAGGGATTTGGGGTGATGGTGGCGGTGTCCAAGTCGTAATCTCGTAGGAAATCTAGGGCAGGGGAGGCGATCTTTCCATCTGCGTGCGT
ATGCATTGTCATCTTACGAAGCGAAGCCTCCTCGGATATCTCAACGAGAAAAGCCTCATCCACAGTTTTAGCCATCCTGGGTTTCTTGGT
TCCTTCTTGCTGGTTGTCATCAGACGATCTCTTTTGTCCCTGCTTCGTGTCTCGCTTCTCCTTGGTGCCGCGCACCTCAACCGTTTTTCC
CGCGTCCTGGATTGAGGTCTGCCGTTCGCTCAGTCTTCCGTGTTCGTGCTCATTGTCACTCGAATCTCCCGTAGGTTTCTTCACCTTGCG
GGTGCTCGGCGATTGCCACTTTGTCATTGGCATCTCGCTGTTGAACCATGCGTCCACGACGCACACAGGCTGCTTACCGTCGCTAAGCAT
TCGATCGAACCAGTATGCACTTGTATTCAGTTGGATAGCGCTGTCCCAGAGGTTCTGGTAGGTCTTGCTCTTGTCGCGGCCATCCACACC
GTACCAGAAGACGATCCGCTCTCCGGTAACTTTCTCGTTCGGGTGGTATGGAAGCTCGTCGGCGCCGAAAGTCCATTCTTGCTTTGGTCC
ATCTGTGACGGCTTGGCCACGTCGAACTTCCGTGGTACCCTGCCCCCTCACCTCGTTGTTGGGGCCTGCTACACCTCGTAGGGTAGACTC
TCTTCGGAAGGTTAGGAAGGATGCTTTAGAGTATCTGAACTTCCTACCTTCAAACTTGCCACCGAGGGCTACAGGTGCGTGTCCAAACAC
CTCTCGGTCGGTGCGTGGAACCCCATTTTGTGTGAAAGTAAAAGGCCACCTCTTCGATCTTTCGGAGGCACTAAGCAAGACGGTGGCAAC
CAGAATAGCACCTTCATCGAGCAACACAAAGGGGGGAGGTGTCCAATCCTTGTCAGCCAGAATATCATCGTTAATATTCCAGTTGTAGCT
GTATACCACCCAATCTTCCGGAGCTTCCTTTCCCTCCTTTGCTTTCTCGTAGATGGCAGAAGCCGGTATGTCTACAACATTGAGGCTGTC
ACTTGGAGACCACCACACGCGGCTTAGACCGTGTTTGGCAACTGGTGGGCCATCGAGTGCATGTTCTGGAACTTGGACCCCCCATGACTT
GGGGGTCTTCAGTTTGAGCTTGTGGTTTGCGCTTTTTGCCATCTCGGTGGCTTCCGCTTGAGTAGGTTGGTCTCGATCGGTAGTTGACGA
ATTGATCTGTAGACGTTGAGAGCGGTTATCGATCGTCGATTTAAGGGTGGGAGACTGGGTTGGGTCTTTCGCAGTTGTGCCTTTTAGGCG
ATCGATTGTTTTCACTGCACGTGACACCTGAGACTGTGTCGGTTGCAGTGAAGGGCTTTTTAGTGGCACCGTTTCCAGGGTGGTAGGCAC
CTCAAGTGTTCCCCCTTGCTGGAAGATCCTGCTGCCTTTCCTTCTATGATGTTGCATAAGTGTAGTGTCGTCTGAATCAGATGACAATTC
TACTATTTCACCTATAGAAATGGTGGCCATTTCTCGTTGTCGTTGAAAACGTGTCTTCGATTGATTTCTGGTTGCTGGCGGGATATTAGA
CACACCTTCTTCGCTTGATAGCTGGTCTCCTCCTGTATGCTCACGCGTAGCAGTACCATAAAAAGCCCTCTGGGTGGCAAGAGCCGTCTG
GCCTGGTAGGAACCTTTCTCGGCTTGACGCCTGTATGTCGGGTAGAATTCCTAATCCACGATATTGTGCTTCTGCCCACTGTCGCGGTTG
GGCCCGTCCTGCTAGCAGTGCTCCTAGCAACCGATCGTAGAACAATCTTCCGAACAGATTCGCACCGAGTGGCTCCTCTGTTTGACCGGC
CATGCCTGGGATGAGTTTATGGTGGATGGCCATAAGACCTTGAATACGGGGATCATCCAGTCGGTATTGGTACGTCAACGCGTCCATCGC
GTTGAATTGAGTACGCCAGTCGAACTCGTTTGGAAAGGCGCACTCTGACTGATTAAACTGGTCGACTTGGTAATAGTGGTTCCCGTCTGG
CAGCGGTAGCAGCTGGTCTAGGTCGTTAGGATCATCTTCTCCCGTCGCTTGAGGAGTACTCAACCACTCTTGCACGCGTTTTTCAGATGG
TATACGTGACTCGGCGACAACTCCATCGTCTTCCATCGTCTGGGCCGTTGGTCTCGAACCTCGAATAGGTTTGAAGTAGGAGTTCTGGTC
GTGGTTATCGGCTTCAAACCTCTTCACTGGCCACTTGATACCGCGGTCCTCCCAGAGAAACCGCTGTGTATCGGTTTTCCAGACGATTCC
GGATTTGACCAGACCTGCCTTGATGTCTTCCACTTCACAAGTACCACGCCTGATAGCTTTCAGCAGTTGCATAAAGAACCATTCCCCCCA
CCGTAACCGCTTTGGACCTCGCTGCTCTAGACTCACTAGATCCGATGGTCGTTTAGGGGCATAGCTCTGGTAGAAGGTGAGGAGATTGAC
GTAGCCCGTATCTGTGAAGGAGCAAAACTGCTTTTGTAGCTTACGATCCTTTTTATAGAGTGCCATCCAGTCGAAGTCTCGCGGCCACGG
GTTCGGTGGTTGATGTGTATAGAAAAAATATGTGCTCGTGCGTTCCTCATCGGATCTCCAGGTGTTGTGGGTAGCAGATGAGTGAGCATC
ATCTGCGTCTTCTGGATCTGGTTGCGTCCCCGTAGTGTCTGGTGACTGTGGCTCCGGCTCTTCGTCGTCTTCGGCTTCTTCTTCTTCTTC
GTCGCCCTCTTCCGAGGTAGAAGCACGCTCGATGTTGGTGAGAGAACGTGCCACTAGCTGGTTACCCGAAATCGTCCGTCGGTTTCCTTC
CTCAGCGCCTCGCTTGGAACTCATcctggcagatttctcgctattcaccaggcagcaactaccttcaacactaatattccagcttgcgac
tgctttctacaccgtgtaaagatgatcctcagctgtcactcccgactcgactgtgggtgcgagcccagtcgccaaaaggtcaggtgacgt
ctcaggccaacggtgcgattccatagcaaccatgattgggtgcaatcactacaacgcccgtgtggttcgccaagctccagcttattcatg
caaaccatcaggtgtcctaccatacaagccagtgaaataacttctccccatgcttttctattccgtaccagcaaatctcattcttcgtac
agactgtcttcctcttcaccgcgtttcttggtaggtcggcctggtggacgtcgtcgctcgaccttctcaaccctgctggggccgttggtc
gatcgcatttacaa...

4 Supplementary References

Bluhm, B., Kim, H., Butchko, R., and Woloshuk, C. (2008). Involvement of ZFR1 of *Fusarium verticillioides* in kernel colonization and the regulation of FST1, a putative sugar transporter gene required for fumonisin biosynthesis on maize kernels. *Mol Plant Pathol* 9(2)**,** 203-211. doi: 10.1111/j.1364-3703.2007.00458.x.

McCluskey, K., Wiest, A., and Plamann, M. (2010). The Fungal Genetics Stock Center: a repository for 50 years of fungal genetics research. *J Biosci* 35(1)**,** 119-126. doi: 10.1007/s12038-010-0014-6.

Nai, C., Wong, H.Y., Pannenbecker, A., Broughton, W.J., Benoit, I., de Vries, R.P., et al. (2013). Nutritional physiology of a rock-inhabiting, model microcolonial fungus from an ancestral lineage of the Chaetothyriales (Ascomycetes). *Fungal Genet Biol* 56**,** 54-66. doi: 10.1016/j.fgb.2013.04.001.

Nødvig, C.S., Hoof, J.B., Kogle, M.E., Jarczynska, Z.D., Lehmbeck, J., Klitgaard, D.K., et al. (2018). Efficient oligo nucleotide mediated CRISPR-Cas9 gene editing in *Aspergilli*. *Fungal Genet Biol* 115**,** 78-89. doi: 10.1016/j.fgb.2018.01.004.

Nødvig, C.S., Nielsen, J.B., Kogle, M.E., and Mortensen, U.H. (2015). A CRISPR-Cas9 system for genetic engineering of filamentous fungi. *Plos One* 10(7)**,** e0133085. doi: 10.1371/journal.pone.0133085.

Schumacher, J. (2012). Tools for *Botrytis cinerea*: new expression vectors make the gray mold fungus more accessible to cell biology approaches. *Fungal Genet Biol* 49(6)**,** 483-497. doi: 10.1016/j.fgb.2012.03.005.

Sweigard, J., Chumley, F., Carroll, A., Farrall, L., and Valent, B. (1997). A series of vectors for fungal transformation. *Fungal Genet Newsl***,** 52-53. doi: 10.4148/1941-4765.1287.

Voigt, O., Knabe, N., Nitsche, S., Erdmann, E.A., Schumacher, J., and Gorbushina, A.A. (2020). An advanced genetic toolkit for exploring the biology of the rock-inhabiting black fungus *Knufia petricola*. *Sci Rep* 10(1). doi: 10.1038/s41598-020-79120-5.
